# Dual CRISPR-Cas3 system for inducing multi-exon skipping in DMD patient-derived iPSCs

**DOI:** 10.1016/j.stemcr.2023.07.007

**Published:** 2023-08-24

**Authors:** Yuto Kita, Yuya Okuzaki, Youichi Naoe, Joseph Lee, Uikyu Bang, Natsumi Okawa, Akane Ichiki, Tatsuya Jonouchi, Hidetoshi Sakurai, Yusuke Kojima, Akitsu Hotta

**Affiliations:** 1 Center for iPS Cell Research and Application, Kyoto University, 53 Shogoin-Kawahara-cho, Sakyo-ku, Kyoto 606-8507, Japan; 2 Nagoya University Graduate School of Bioagricultural Sciences, Avian Bioscience Research Center, Furo-cho, Chikusa-ku, Nagoya, Aishi 464-8601, Japan; 3 Takeda-CiRA Joint Program (T-CiRA), Fujisawa, Kanagawa 251-8555, Japan

**Keywords:** DMD, CRISPR-Cas3, genome editing, gene therapy, exon skipping

## Abstract

To restore dystrophin protein in various mutation patterns of Duchenne muscular dystrophy (DMD), the multi-exon skipping (MES) approach has been investigated. However, only limited techniques are available to induce a large deletion to cover the target exons spread over several hundred kilobases. Here, we utilized the CRISPR-Cas3 system for MES induction and showed that dual crRNAs could induce a large deletion at the dystrophin exon 45–55 region (∼340 kb), which can be applied to various types of DMD patients. We developed a two-color SSA-based reporter system for Cas3 to enrich the genome-edited cell population and demonstrated that MES induction restored dystrophin protein in DMD-iPSCs with three distinct mutations. Whole-genome sequencing and distance analysis detected no significant off-target deletion near the putative crRNA binding sites. Altogether, dual CRISPR-Cas3 is a promising tool to induce a gigantic genomic deletion and restore dystrophin protein via MES induction.

## Highlights

• Dual CRISPR-Cas3 induces several hundred kilobases of genomic DNA deletion
• SSA-based two-color reporter system facilitates the isolation of genome-edited cells
• Multi-exon skipping recovered the dystrophin protein in multiple DMD-iPSC lines
• Whole-genome sequencing detected no off-target mutations associated with Cas3

## Graphical abstract

**Figure:**
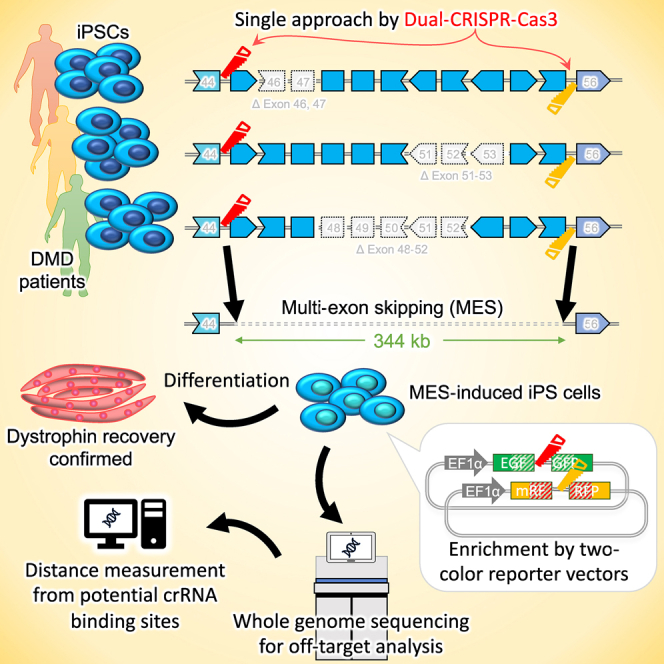

## Teaser

Kita et al. utilize the dual CRISPR-Cas3 system to induce a large deletion (∼340 kb) at the dystrophin exon 45–55 region for multi-exon skipping (MES) in various Duchenne muscular dystrophy (DMD) mutation patterns. Dual-Cas3-based MES restored dystrophin protein in DMD-iPSCs without significant off-target deletion. Dual CRISPR-Cas3 is a promising tool for MES induction to restore dystrophin protein in DMD.

## Introduction

Duchenne muscular dystrophy (DMD) is a severe muscle degeneration disorder caused by genomic mutations that result in a frameshift in the *dystrophin* gene (Koenig et al., 1988). DMD is known to be the most frequent and severe type of muscular dystrophy (Mercuri et al., 2019); however, there is no curative treatment so far. The *dystrophin* gene is one of the largest protein-coding genes (∼2 Mb) in the human genome, in which the skeletal muscle isoform of Dp427m consists of 79 exons translated to a 427-kDa dystrophin protein (Muntoni et al., 2003). The dystrophin protein stabilizes muscle cells or myofibers by binding to the actin cytoskeleton on the N terminus and the muscle cell membrane on the C terminus (Fairclough et al., 2013). So far, more than 7,000 kinds of mutations in the *dystrophin* gene have been identified in DMD patients (Bladen et al., 2015), and such mutations disrupt the open reading frame and lead to the loss of functional dystrophin protein.

To restore dystrophin protein, exon skipping is a promising approach to correct the open reading frame. There are already a couple of antisense oligonucleotide (ASO)-based exon skipping drugs clinically approved for DMD treatment (Roshmi and Yokota, 2021). However, the ASO strategy skips exons at the RNA level and doesn’t correct causal genomic mutations; hence, DMD patients require repeated weekly injections throughout their life.

Recently, the induction of exon skipping at the genomic DNA level has been expected to be an emerging approach that can have a longer-lasting effect than ASO. Especially the development of the CRISPR-Cas9 system has revolutionized the field of genome editing (Doudna, 2020). Many groups have utilized the CRISPR-Cas9 system to induce genomic mono-exon skipping *in vitro* (Kagita et al., 2021; Li et al., 2015; Ousterout et al., 2015) and *in vivo* (Amoasii et al., 2017; El Refaey et al., 2017; Gee et al., 2020; Kenjo et al., 2021; Koo et al., 2018; Long et al., 2016; Min et al., 2019; Nelson et al., 2016; Tabebordbar et al., 2016; Xu et al., 2019; Zhang et al., 2020).

However, due to the significant variations of the mutation patterns in the *dystrophin* gene, the mono-exon skipping approach can only be used for a limited number of DMD patients. For example, the most common mono-exon skipping of exons 51, 53, and 45 can be applied to 13%, 8%, and 8% of DMD patients, respectively (Saifullah et al., 2022). To broaden the patient applicability, the multi-exon skipping (MES) approach by targeting the exons from 45 to 55 has been proposed (Béroud et al., 2007). By targeting the mutation hotspots in the *dystrophin*, MES from exon 45 to 55 was estimated to restore the *dystrophin* open reading frame in more than 60% of DMD patients (Echigoya et al., 2018). Although this approach produces a shorter dystrophin protein, multiple clinical studies have reported that an in-frame deletion of exon 45–55 results in a very mild phenotype or sometimes asymptomatic even in their 60s (Ferreiro et al., 2009; Nakamura et al., 2008). To induce MES at the RNA level, a cocktail of 11 ASOs has been demonstrated to restore dystrophin protein, therefore improving muscle function in a mouse model (Aoki et al., 2012). To develop a long-lasting drug for inducing MES at the genomic DNA level, it is required to remove the exon 45–55 region, which spans at least 344 kb in the human chromosome (Young et al., 2016). However, the induction method to induce several hundred kilobases genomic deletions has not been thoroughly investigated.

Recently, we and others have shown that class 1 type I-E CRISPR-Cas3 can be utilized as a genome editing tool in mammalian cells (Cameron et al., 2019; Dolan et al., 2019; Morisaka et al., 2019). Unlike the class 2 type II CRISPR-Cas9 system, the type I-E CRISPR-Cas3 system consists of the multi-subunit complex, Cascade/crRNA, to bind the target sequence, and Cas3 enzyme with the DNA helicase and nickase activity to shred the target DNA processively toward PAM proximal side (Loeff et al., 2018). Thus, CRISPR-Cas3 induces a long range of deletions from a few hundred base pairs to a hundred kilobase pairs from the crRNA target site in a unidirectional manner. By utilizing this property, we previously demonstrated that a single Cas3-crRNA induced genomic mono-exon skipping in the *dystrophin* gene (Morisaka et al., 2019); however, targeted genomic deletion of several hundred kilobases and the feasibility of MES were unexplored.

In this study, we sought to induce genomic MES by multiplexing Cas3-crRNAs. We found that dual-Cas3 can cause a large deletion of up to 340 kb. We investigated the optimal crRNA combinations for MES induction in DMD-iPSCs and developed a reporter system to enrich genome-edited cells. Finally, to examine the risk of off-target mutagenesis, we performed whole-genome sequencing of the genome-edited subclones and investigated the deletions associated with putative crRNA binding sites. These results suggest the potential of the dual CRISPR-Cas3 system to induce large genomic deletions for MES induction in DMD patients with a wide variety of mutation patterns.

## Results

### Dual CRISPR-Cas3 to induce several hundred kilobases of deletions

**Figure 1:**
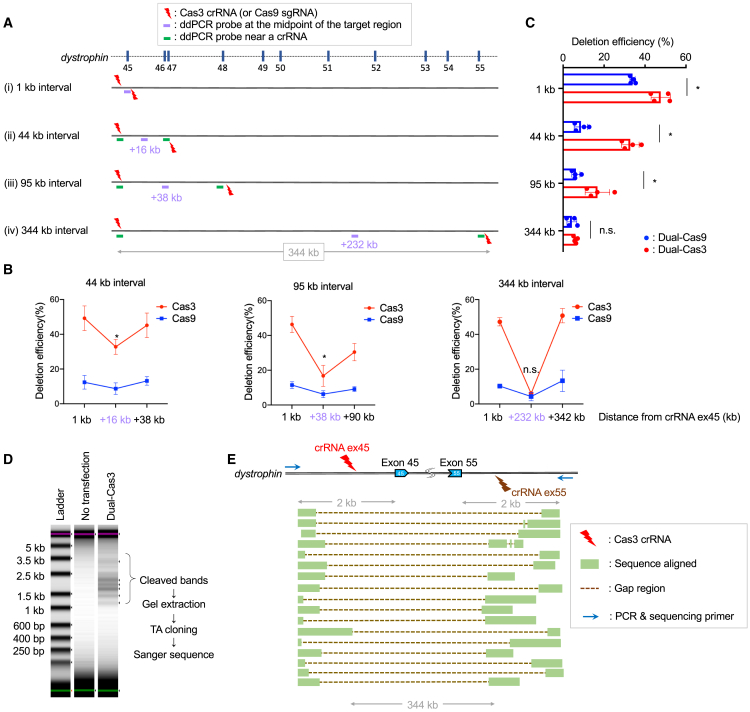
Induction of a large deletion by the dual-Cas3 system (A) Schematic overview of the experiments to compare the deletion efficiency between Cas3 and Cas9 at various distances. Multiple pairs of Cas3-crRNAs or Cas9-sgRNAs were designed upstream and downstream of the target region, as indicated by red thunder marks. The orientation of the edge of the thunder mark indicates the PAM sequence, and Cas3 induces deletion in this direction. Each target region is (i) 1 kb, (ii) 44 kb, (iii) 95 kb, and (iv) 344 kb distal between two crRNA (or sgRNA) targeting sites at the *dystrophin* gene locus. To quantify deletion efficiencies, ddPCR probes were designed at the approximate midpoint of the target region, shown as a purple horizontal bar, and near the crRNA binding site, shown as a green horizontal bar. (B) The deletion efficiency is calculated as DNA copy number loss by ddPCR at the indicated position in HEK293T cells. The activity of each crRNA was examined by the ddPCR probes indicated in (A) as green bars. Copy number loss at the approximate midpoint of the target region (+16 kb, +38 kb, and +232 kb probes for the 44-kb, 95-kb, and 344-kb intervals, respectively) is considered as the induction of large deletion. Data were represented as means ± SD from independent experiments (n = 4). Two-tailed unpaired t test was used to calculate p values. ^∗^p < 0.05. (C) The summary of the deletion induction efficiency at the midpoint from (B). Data were represented as means ± SD from independent experiments (n = 4). A two-tailed unpaired t test was used to calculate p values. ^∗^p < 0.05. (D) PCR analysis to investigate the deletion patterns in HEK293T. After transfection of a pair of Cas3-crRNA, the *dystrophin* exon 45–55 region was PCR amplified using indicated primers and visualized by TapeStation with high-sensitivity D5000 DNA tape. (E) Sanger sequencing results of PCR amplicons from (D). Ladder PCR amplicons were subjected to TA cloning, and each clone was analyzed by Sanger sequencing (17 clones). Matching regions to the *dystrophin* gene are shown in green boxed lines, and gap regions are shown as broken lines.

Since it was rare to observe a deletion of more than a hundred kilobases using a single Cas3-crRNA, we sought to use a pair of crRNAs inwardly sandwiching the target genomic region. To assess how this dual-Cas3 approach could feasibly induce large deletions, we designed one crRNA fixed near the exon 45 of the *dystrophin* gene and its counterpart crRNAs at 1 kb, 44 kb, 95 kb, and 344 kb positions from the fixed crRNA (Figures 1A and S1A and Table S1). As a comparison, we also designed Cas9-sgRNAs in similar positions. After transfection of plasmid DNA expression vectors of Cas3/Cascade/crRNAs or Cas9/sgRNAs, we measured genomic copy numbers at the approximately halfway points of the crRNA pairs using droplet digital PCR (ddPCR) to quantify deletion efficiency (Figure 1A, purple bars). The deletion efficiencies at proximal positions to designed crRNA (within 6 kb) were also monitored to validate the activities of crRNAs (Figure 1A, green bars). In HEK293T cells, the losses of DNA copy number at the exon 45 (+1 kb probe) and exon 55 (+342 kb probe) were more evident with dual-Cas3 than dual-Cas9 (Figure 1B). In addition, the copy number losses at the half-points were significantly higher with 44-kb and 95-kb intervals. The apparent copy number loss with the 344-kb interval was also clearly detectable using dual-Cas3, though there was no statistical difference between Cas3 and Cas9 (Figure 1C). In addition to the inward dual-Cas3 system, we also tested parallel and outward orientations for the dual-Cas3 (Figure S1B). Unexpectedly, we found that the parallel and outward dual-Cas3 could also induce large deletions at similar efficiency to the inward manner. To avoid unnecessary deletions outside of the crRNA target sites, we decided to use the inward dual-crRNA method in the following experiments.

Next, we examined the deletion patterns induced by the inward dual-Cas3 with the 344-kb interval in HEK293T cells. For this, the target genomic region was amplified using the PCR primers upstream of exon 45 and downstream of exon 55. Due to the long distance between the two primers, amplification occurs only when large deletions are induced. Indeed, PCR results showed multiple bands in the genome-edited cells but not in the untreated cells (Figure 1D). These bands were gel-extracted and sequenced after TA cloning. As a result, we observed various patterns of deletions around 344 kb in size (Figure 1E). Interestingly, in many cases, the deletion extended beyond the crRNA target sites; therefore, both crRNA binding sites were lost. This contrasts with the single Cas3-crRNA, as crRNA binding sites are usually maintained (Morisaka et al., 2019).

In addition to HEK293T cells, we also performed genome editing with the dual-Cas3 in the DMD patient-derived iPSC line FF12020, which lacks exons 46 and 47 (Δexon 46, 47). PCR analysis of the target region similarly showed ladder band patterns (Figure S1C), and Sanger sequencing of the multiple PCR products following TA cloning showed large deletions spanning the exons 45 and 55 at the *dystrophin* gene (Figure S1D). Altogether, we demonstrated that the inward dual-Cas3 strategy successfully induced up to 344-kb large deletions not only in HEK293T cells but also in DMD patient-derived iPSCs.

### Enrichment of the dual-Cas3-edited cells using single-strand annealing reporter

Although we successfully induced 344-kb large deletions by Cas3, the efficiency in generating such a gigantic deletion was not high enough for subcloning in DMD patient-derived iPSCs (Figure S3G). In previous studies, genome-edited cell populations by Cas9 could be enriched using single-strand annealing (SSA) reporter vector followed by cell sorting (Mashiko et al., 2013). Therefore, we sought to enrich the genome-edited cell population using a similar system adapted for our dual-Cas3.

**Figure 2:**
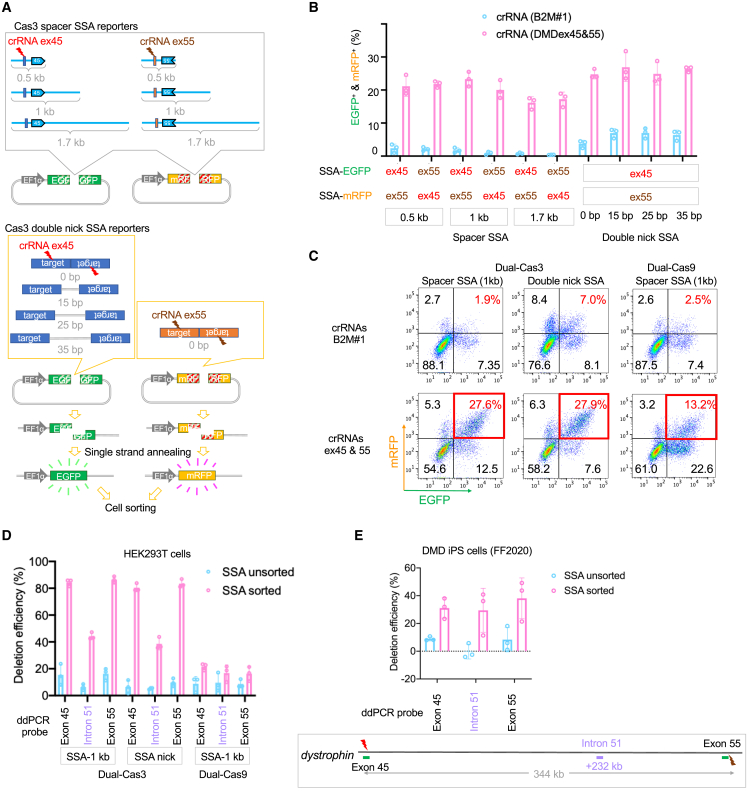
Enrichment of Cas3-active cells by single-strand annealing (SSA) reporter (A) Schematic of various SSA reporter vectors for monitoring dual-Cas3’s activity. Structures of SSA vectors with long inserts with various insert lengths (0.5, 1.0, and 1.7 kb) or double-nick inserts containing a spacer (0, 15, 25, and 35 bp) are constructed. When Cas3 or Cas9 induces a DNA break at the target sequence (blue boxed bar), the single-strand annealing (SSA) DNA repair pathway restores the functional cDNA sequence of EGFP or mRFP through the two homology sequences (indicated by the slashed box). (B) Evaluation of the SSA vectors by the dual-Cas3 system. Two-color SSA vectors (EGFP or mRFP) containing a crRNA target sequence (DMD ex45 or ex55) were transfected into HEK293T cells together with the expression vectors of Cas3, Cascade, and two crRNAs that target DMD ex45 and ex55, as “targeting crRNA.” As negative control to evaluate the background fluorescence activity of the SSA reporters, we used a crRNA that targets the B2M gene, as “non-targeting crRNA.” 3 days after transfection, the percentages of EGFP and mRFP double-positive cells were analyzed by flow cytometry. Data were represented as means ± SD from independent experiments (n = 3). (C) Representative FACS plots and gating for cell sorting. After transfection of the SSA vectors (1-kb-long spacer or double nick with 0-bp spacer) as described in (B), cell sorting of the EGFP and mRFP double-positive population (red rectangle area) was performed. As a control, we used Cas9 and two sgRNA-expression vectors targeting exons 45 and 55. (D) The deletion efficiency in the sorted cells with the two-color SSA vectors. Genomic DNAs of sorted HEK293T cells were subjected to ddPCR analysis to check the deletion efficiency with the ddPCR probes at exons 45 (+1 kb), 55 (+342 kb), and the midpoint (+232 kb). Data were represented as means ± SD from independent experiments (n = 3). (E) The deletion efficiency in DMD patient-derived iPSC FF12020 line sorted by the two-color SSA reporter system. Two SSA vectors (EGFP for exon 45 target, mRFP for exon 55 target) with 1-kb spacer were transfected into FF12020 iPSCs, and cell sorting was performed 3 days post-transfection. The sorted cells were similarly analyzed by ddPCR as described in (D). Data were represented as means ± SD from independent experiments (n = 3).

In the SSA reporter system, a spacer sequence containing a crRNA target site was inserted into a split fluorescent protein gene with homology sequences for SSA repair after DNA damage. First, we considered the optimal sequence length of the target insert for Cas3 because Cas3 induces large deletions rather than small deletions. The short target sequences typically used for Cas9 (e.g., just the crRNA recognition site) might not be suitable for Cas3. Therefore, we inserted relatively long spacer sequences (0.5–1.7 kb) containing the crRNA recognition site and extended the target sequence in the direction of Cas3 deletion (Figure 2A). In addition to the spacer SSA reporter, we also constructed double-nick-based SSA reporters, considering that Cas3 is a nickase. We hypothesized that a palindromic crRNA recognition site with a spacer might induce DNA double-strand break by induction of double nicking at each DNA strand (Figure 2A). Lastly, to monitor the activity of both sides of the crRNAs in the dual-Cas3 system, we constructed both EGFP (enhanced green fluorescence protein) and mRFP (monomeric red fluorescence protein) versions of the SSA vectors. We used these vectors and two Cas3-crRNAs to perform two-color SSA reporter assays in HEK293T cells. Our results showed significantly higher EGFP and mRFP signals only when using targeting crRNAs (DMD ex45 and ex55) but not with a non-targeting crRNA (B2M #1) (Figure 2B). Among the spacer SSA vectors, the background signals with the non-targeting crRNA were decreased when the insert spacer was elongated from 0.5 to 1.7 kb. Although the double-positive signals were detected with the three spacer SSA reporters, a slight decrease was observed with the 1.7 kb spacer. All double-nick SSA reporters showed higher on-target and background signals than long target SSA vectors, regardless of a spacer length. Therefore, we chose the target insert with 1 kb long for the spacer SSA reporters and no spacer sequence for double-nick SSA reporters because of their relatively high signal-to-noise ratio.

Next, we tested whether the genome-edited population can be enriched by cell sorting utilizing the two-color SSA reporter vectors in HEK293T cells. After transfection of the Cas3/Cascade/crRNA expression vectors and the two-color SSA vectors, EGFP and mRFP double-positive population was sorted by a cell sorter (Figure 2C). Genome editing efficiency of ∼340 kb deletion in HEK293T cells was estimated by ddPCR measuring copy number at the midpoint of 340 kb (intron 51, +232 kb). Compared with the unsorted population, the sorted population showed significantly higher deletion efficiency, up to 40% (Figure 2D). Similar enrichment was also observed in DMD patient-derived iPSC line FF12020 using 1-kb-long target SSA reporters, and around 30% of copy number loss was seen at the midpoint (Figure 2E). Taken together, we demonstrated that the two-color SSA reporter system can enrich the genome-edited population by dual-Cas3 to facilitate the isolation of cells with a large deletion.

### MES induction with multiplexed Cas3-crRNAs

In addition to the dual-Cas3 approach (2× crRNAs), we also tested whether using more than two crRNAs enhances the efficiency of ∼340-kb large deletion. For this, we designed four crRNAs (4× crRNAs) with about 110-kb intervals spanning the 340-kb region and 11 crRNAs (11× crRNAs) targeting all 11 exons between 45 and 55 exons (Figure S2A). In HEK293T cells, 4× and 11× crRNAs showed greater deletion efficiencies at the middle five probes when assessed by ddPCR (Figure S2B). However, when similar genome editing using 2×, 4×, and 11× crRNAs were performed in DMD-iPSC line FF12020, the recovery rate of dystrophin protein after muscle differentiation in 4× and 11× crRNA samples did not increase compared with the 2× crRNA sample by western blotting (Figures S2C and S2D). In addition, we performed immunostaining to detect dystrophin protein recovery. However, the trend of the dystrophin recovery rate was not consistent among 2×, 4×, and 11× crRNA samples (Figures S2E and S2F). We speculate that a variety of deletion patterns caused by multiple crRNAs would lead to suboptimal in-frame deletions; therefore dystrophin protein recovery did not match with the ddPCR results. Because of these results and the complexity of using multiple crRNAs, we decided to use the dual-Cas3 approach for downstream experiments.

### MES induction in various DMD patient-derived iPSCs

**Figure 3:**
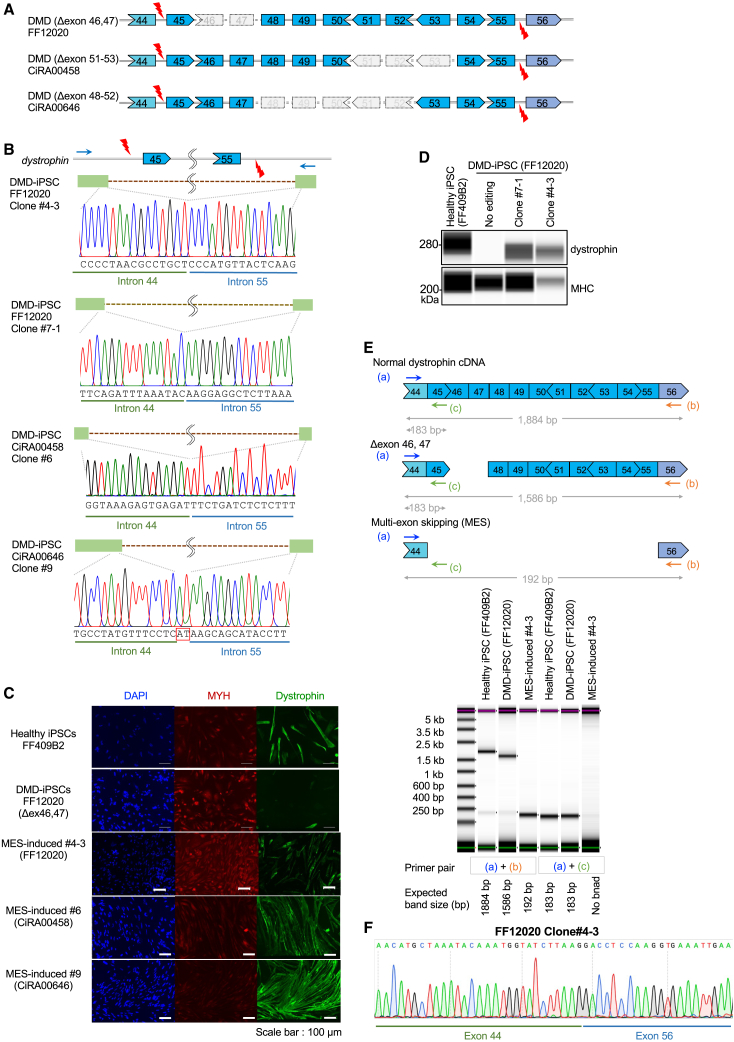
The dual-Cas3 genomic MES induction in multiple patient-derived iPSCs (A) Schematic illustrations of MES induction in three DMD patients. Three DMD-iPSC clones—FF12020 iPSC clone (Δexon 46, 47), CiRA00458 clone (Δexon 51–53), and CiRA00646 clone (Δexon 48–52)—were subject to dual-Cas3 genome editing using two crRNAs indicated by thunder marks. The shapes of exons illustrate codon frames. (B) Deletion patterns of genomic MES-induced DMD-iPSC subclones. Genomic MES-induced iPSC subclones (FF12020 #4–3, #7–1, CiRA00458 #6, and CiRA00646 #9) were established by the dual-Cas3 genome editing followed by the two-color SSA-based single-cell sorting. Genomic DNA samples were PCR amplified using indicated primers (arrows), and deletion patterns were analyzed by Sanger sequencing. A red square indicates the insertion sequence. (C) Confirmation of dystrophin protein recovery by immunocytochemical staining. Genomic MES-induced subclones in (B) were differentiated into skeletal muscle cells by MYOD1 expression, and immunocytochemical staining was performed for MYH and dystrophin. Nuclear staining was performed with DAPI. Healthy donor-derived iPSCs (FF409B2) and unedited FF12020 iPSC are shown as controls. Scale bars indicate 100 μm. (D) Confirmation of dystrophin protein recovery by Wes automated western blotting system. Healthy iPSC (FF409B2), DMD-iPSC (FF12020: Δexon 46, 47), and genomic MES-induced clones #7–1 and #4–3 were differentiated into skeletal muscle cells, and dystrophin and MHC proteins were detected by the Wes system. (E) Confirmation of multi-exon skipping at mRNA level by RT-PCR. Primers to distinguish MES-induced mRNA from uninduced ones are indicated (top). RNA samples from healthy donor iPSC (FF409B2), DMD-iPSC (FF12020: Δexon 46, 47), and MES-induced FF12020 subclone #4–3 were reverse-transcribed and PCR amplified using indicated primer pairs. TapeStation result is shown, and expected band sizes are shown below. (F) Direct sequencing of cDNA from genomic MES-induced clone #4–3. PCR product from #4–3 in (D) amplified by primer (a)+(b) was Sanger sequenced. In-frame junction of exon 44 and exon 56 is shown.

The advantage of the MES strategy is its broad applicability to various DMD mutation types located within exon 45–55. To demonstrate this, in addition to the FF12020 iPSC line (Δexon 46, 47), we established two additional iPSC lines, CiRA00458 (Δexon 51–53) and CiRA00646 (Δexon 48–52), from DMD patients (Figures S3A and S3B). With a total of three iPSC lines, we performed dual-Cas3-based genome editing (Figure 3A), followed by enrichment with the two-color SSA reporters and single-cell sorting. After subcloning, PCR genotyping (Figures S3C–S3F), and Sanger sequencing (Figures 3B and S3E), we successfully obtained subclones with exon 45–55 skipping from all the parental iPSC lines (Figure S3G and S3H: 21 clones out of 98 clones screened for FF12020, one clone out of three clones for CiRA00458, and one clone out of six clones for CiRA00646). Next, we confirmed the dystrophin protein recovery of the subclones (FF12020 #4–3, #7–1, CiRA00458 #6, CiRA00646 #9) by skeletal muscle differentiation. Immunocytochemical staining results showed the apparent restoration of dystrophin protein by the dual-Cas3-mediated deletion in the subclones (Figure 3C). Furthermore, we also confirmed the dystrophin protein recovery by the western blotting system using FF12020 #4–3 and #7–1 (Figure 3D). In this analysis, a band shift of the dystrophin protein due to the truncation of the exon 45–55 region was observed in these subclone samples. RT-PCR also confirmed the induction of MES at the mRNA level in the FF12020 #4-3 clone (Figure 3E). Sanger sequencing of the cDNA from #4-3 clone clearly showed the in-frame junction of exons 44 and 56 (Figure 3F).

Overall, these results demonstrated that the dual-Cas3 system was able to induce the genomic MES in DMD-iPSCs with various mutations and restored dystrophin protein, emphasizing the broad applicability of our dual-Cas3 strategy.

### Off-target analysis of the dual-Cas3 by whole-genome sequencing

**Figure 4:**
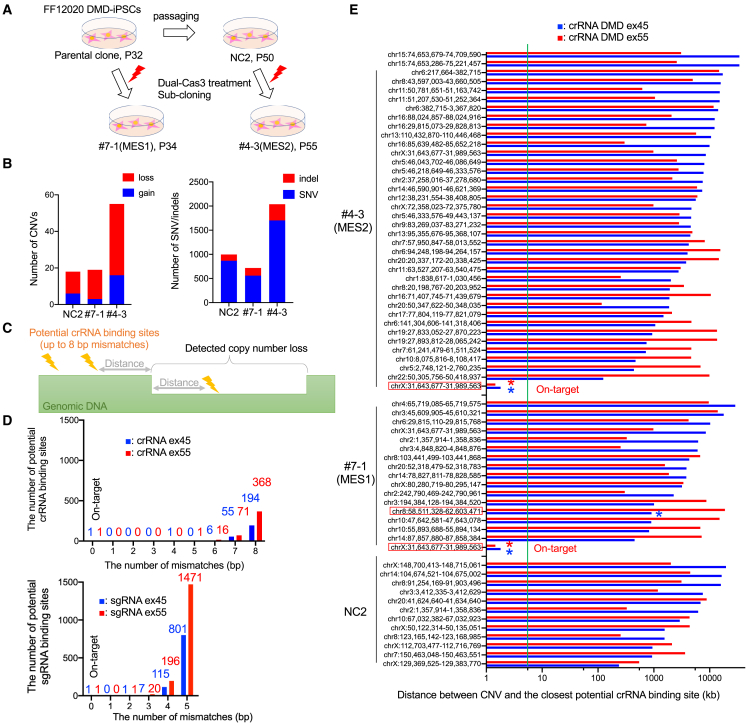
Whole-genome sequencing for detecting off-target cleavages by dual-Cas3 (A) Schematic diagram of the clones assessed by whole-genome sequencing. Two genomic MES-induced FF12020 subclones (#7–1 and #4–3) were established from their parental FF12020 cells at different passage numbers (NC1 at p32, NC2 at p50). These four samples were subject to whole-genome sequencing. (B) Summary of detected CNVs, indels, and SNVs. The number of *de novo* CNVs (left) and indel/SNVs (right) were counted using NC1 data as a reference. (C) Schematic of distance calculation between potential crRNA binding sites and the edge of detected CNVs. To estimate bona fide off-target CNVs induced by Cas3, the distance between the closest crRNA binding site and a detected CNV was calculated. (D) The number of potential Cas3 crRNA/Cas9-sgRNA binding sites. Potential crRNA binding sites of two crRNAs targeting exon 45 and exon 55 were searched by GGGenome software with an 8-base mismatches allowance. A total of 256 potential off-target sites for ex45 and 456 sites for crRNA ex55 were found in the human genome (top). Potential sgRNA binding sites of two sgRNAs targeting exon 45 and exon 55 were searched by GGGenome with a 5-base mismatches allowance. A total of 924 and 1,688 off-target sites for ex45 and ex55 were found, respectively (bottom). (E) Distances between the detected CNVs and the closest potential crRNA off-target sites. Blue bars indicate the distance from the potential binding sites for crRNA ex45, and red bars indicate the distance from the potential binding sites for crRNA ex55. As a threshold window, the 5-kb line (within which 99% of the Cas3 cleavage starts) is indicated as a green vertical line. The asterisk indicates the potential crRNA binding site detected within the CNV.

Finally, we assessed the potential off-target risks caused by the dual-Cas3 in the genomic MES-induced subclones. To distinguish off-target mutations from spontaneous mutations induced by cell culturing and subcloning, we prepared two sets of samples (Figure 4A). MES-induced subclone #7–1 was established from unedited parental DMD patient-derived iPSC line FF12020 at passage 32 (NC1), and another MES-induced subclone #4–3 was established from the FF12020 at passage 50 (NC2). Whole-genome sequencing was performed using these four samples with an average sequencing depth of 36.9. CNVs (copy number variations), indels (insertions and deletions), and SNVs (single nucleotide variations) were detected by using the NC1 sample as a reference (Tables S3 and S4). We confirmed that the on-target 344-kb deletion induced by dual-Cas3 was clearly detected as a CNV in the #7–1 and #4-3 clones (Table S3, CNV ID: 77).

As previously reported (Martins-Taylor et al., 2011), some *de novo* CNVs were detected in the NC2 sample, in which the only difference is the passage number of 18 (Figure 4B). Next, to distinguish Cas3-mediated CNVs from spontaneous ones, we hypothesized that Cas3-induced mutations should accompany a putative crRNA binding site nearby. For this, we evaluated the distance of each detected CNV from the nearest potential crRNA binding sites (Figure 4C). In our previous experiences, 99% of the Cas3-mediated deletion events start within 5 kb from a crRNA binding site (Morisaka et al., 2019). Therefore, if detected CNVs are outside of this region, those are most likely not due to the Cas3-crRNA activity.

Firstly, we extracted the list of potential crRNA binding sites for both crRNAs targeting *dystrophin* exon 45 and 55 using GGGenome software, allowing up to 8-base mismatches out of 27-base target sequence recognition, considering that every six base positions are not involved in target sequence recognition. As a result, we identified 257 sites for crRNA ex45 and 457 sites for crRNA ex55 in total (Figure 4D). Of note, the number of potential off-target sites is much less than that of Cas9-sgRNA as many more potential off-target sites were already predicted for Cas9-sgRNAs with up to five mismatches (Figure 4D). This is due to the longer target recognition ability of the Cas3-crRNA (27 nt) compared with Cas9-sgRNA (20 nt). Next, we calculated the distance between a CNV and the nearest potential crRNA binding site (Figure 4E). Among the total 67 CNV losses detected (13 CNVs from NC2, 16 from #7–1, and 38 from #4–3), only the on-target CNVs have the crRNA binding site within the 5-kb window, but all other CNV losses were outside, suggesting that those CNVs were unlikely to be generated by the Cas3/crRNA. Of note, among 85 CNVs identified, one 4,058-kb CNV was found to have a potential crRNA (ex45) binding site inside of the CNV at 908 kb and 3184 kb from upstream and downstream edges, respectively (Figure S4A). This is far beyond the 5-kb window from the potential crRNA binding site. Furthermore, this potential crRNA site has 8-bp mismatches, among which three mismatches are located within the crRNA seed sequence (within 8-bp region from PAM). Considering even one mismatch in the seed sequence almost abolished the activity of Cas3 (Morisaka et al., 2019), it is unlikely the CNV is due to the off-target cleavage.

Although the Cas3 tends to induce a CNV loss (large deletion), we also evaluated the association of Cas3-crRNA with the detected indels and SNVs. Like CNVs, a greater number of SNV/indels were detected in NC2, #7–1, and #4-3 samples compared with the parental NC1. A similar distance calculation of SNV/indels from the closest potential crRNA binding sites revealed that most (99.9%) of the detected SNV/indels were located more than 5 kb away from the potential crRNA binding sites (Figure S4B). Notably, we found one SNV out of 717 SNV/indels in the #7-1 clone, and six SNV/indels out of 2,038 SNV/indels in the #4-3 clone were located within 5 kb from a potential crRNA binding site (Figure S4C). However, all the nearest crRNAs have mismatches of 7 or 8 bp, and half of the crRNAs pointed to the opposite direction of those SNV/indels. Considering that we searched for relatively large 7.14-Mb regions (714 crRNAs, ±5 kb) out of 3.117-Gb genome, we asked whether the odds of finding a few SNV/indels were within the range of chance occurrence. As a result, there was no significant difference in the frequency of SNV/indels appearance between the NC2 and #7–1 or between NC2 and #4–3 (Figure S4D).

Taken together, these whole-genome sequencing analyses demonstrated no apparent off-target cleavage caused by dual-Cas3 system, suggesting the high specificity of the dual-Cas3 system.

## Discussion

MES induction is one of the promising approaches for DMD treatment because of its broad applicability to various mutation patterns. In this study, we took advantage of the unique feature of CRISPR-Cas3 to induce unidirectional deletion and developed a dual Cas3 system for genomic MES induction.

First, we assessed the potential of the dual-Cas3 approach to induce large deletions and showed that dual-Cas3 is available to generate up to ∼340-kb deletion not only in HEK293T but also in iPSCs (Figures 1 and S1).

Second, to facilitate the isolation of cells edited with dual-Cas3 system, we established an SSA reporter-based two-color cell enrichment technique. Using this, we successfully enriched ∼340-kb-deleted cell populations up to 40% in HEK293T cells and 30% in iPSC, respectively (Figures 2D and 2E). Of note, we also tested whether the multiplexed Cas3 system (4× and 11× crRNAs) could further improve the editing efficiency or not. However, it was difficult to draw a conclusion about the efficiency of 4× and 11× crRNA methods because of the discrepancy among ddPCR, western blotting, and immunofluorescence results and no obvious improvement in the recovery efficiency (Figure S2). Therefore, we decided to focus on the dual-Cas3 system for the downstream experiments.

Third, to show the broad applicability of dual-Cas3 system, we induced genomic MES in three different DMD patient-derived iPSCs (Figure 3A). By utilizing the SSA reporters, we successfully isolated genome-edited subclones from those iPSCs, and the restoration of dystrophin protein was confirmed after skeletal muscle differentiation.

Finally, we performed whole-genome sequencing and assessed the potential association of the detected CNVs/indels/SNVs with off-target activity. We searched for the nearest potential crRNA binding site from the breakpoint of the detected CNVs, and all the detected CNVs were distal more than 5 kb (Figure 4E). Consistent with our previous study (Morisaka et al., 2019), highly specific target recognition by 27-base-long crRNA may result in the high specificity of the Cas3 system.

For inducing extremely large deletions, many techniques have been developed previously. For example, the classical approach uses the Cre-loxP system. Although two exogenous *loxP* sequences must be inserted in advance, this method allows efficient and precise large genome editing. In DMD studies, for example, ∼2.4-Mb whole *dystrophin* gene was deleted in mice (Kudoh et al., 2005), and our group also successfully introduced a 342-kb deletion at the *dystrophin* gene (exon 45–55) in iPSCs by Cre-loxP recombination (Kagita et al., 2021).

After the discovery of Cas9, dual-Cas9 system has been reported to induce a large deletion in murine erythroleukemia cells (Canver et al., 2014). In fact, dual-Cas9 has been used in iPSCs for MES induction in the *dystrophin* gene, although the editing efficiency was not shown (Young et al., 2016). Alternatively, several groups reported that dual-prime editors introduced up to 10-kb deletions in HEK293T cells, although the efficiency of 10-kb deletion was still 1%–7% (Choi et al., 2022; Jiang et al., 2022). In addition, large knockin or gene substitution techniques can be potentially applied to induce a large deletion. The ssODN-mediated method was used for the gene substitution up to 58 kb by combining multiple Cas9-sgRNAs and ssODNs in rat embryos (Yoshimi et al., 2016). Furthermore, the two-step Universal Knock-in System was shown to induce more than 200-kb deletion in HCT116 cells (Ohno et al., 2022). These methods use homology-directed repair mechanisms and can potentially generate large deletions, although their availability for MES induction remains to be investigated.

Of note, there are potential limitations of the dual- or multiplexed-Cas3 systems. First, there is a variation in the deletion pattern, and the precise start and endpoints of the deletion cannot be fully controlled (Figure 1E). This could be a drawback when a large but precise deletion is required. However, in the case of DMD, the disparity of the deletion size should be acceptable because the target exons are separated by large intron sequences averaging 28 kb in length.

Another consideration is the MES induction efficiency. In this study, we used plasmid DNA vectors for transfecting the Cas3/Cascade/crRNA expression, together with the SSA reporter vectors. We anticipate investigating other methods to deliver Cas3 into cells that might facilitate the overall genome editing efficiency of the Cas3 system.

Finally, we could not show the functionality of the recovered dystrophin protein, such as interaction with the dystrophin-associated protein complex, in the study. We tried immunostaining with β-dystroglycan but failed to detect the signal (data not shown), presumably because of the immature nature of our MYOD1-overexpression-based skeletal muscle differentiation, compared with primary adult skeletal muscle. In the future, more advanced phenotypic assays will be necessary to show that our dual-Cas3-mediated MES approach is still efficient in myotubes after the differentiation of iPSCs.

Nevertheless, we believe the genomic MES-induced iPSCs would be a cell source for future autologous cell therapy applications by differentiating the iPSCs into muscle cells or muscle progenitors to supplement the damaged muscle tissues in DMD patients (Zhao et al., 2020).

In summary, this study demonstrated that the dual CRISPR-Cas3 system is a powerful tool to induce genomic MES by deleting several hundred kilobases in patient-derived iPSCs. This is the first study to show the feasibility of Cas3 as a potential tool for multi-exon skipping in DMD. We expect this will enlighten new ways to treat DMD patients and other genetic disorders that require extensive deletions.

## Experimental procedures

### Resource availability

#### Corresponding author

Further information and requests for resources and reagents should be directed to and will be fulfilled by the corresponding author, Akitsu Hotta (akitsu.hotta@cira.kyoto-u.ac.jp).

#### Materials availability

Plasmid DNA vectors are deposited in Addgene (https://www.addgene.org/): Cas3/Cascade expression vector pPV-Dual_promoter-EF1α-2xNLS-Cascade+Cas3-P (RD) (Addgene ID: 204619), crRNA expression vector pPV-C1-crRNA(DMD#20_DMD#23)-EF1a-BA (Addgene ID: 204620), SSA reporter vector pPV-EF1a-EGxxFP(DMDex45_10)-iP-A (Addgene ID: 204621), pPV-EF1α-mRxxFP1(DMDex55_10)-iP-A (Addgene ID: 204622), crRNA cloning vector pPV-C1-crRNA-cloning-EF1a-BA (Addgene ID: 204623), SSA cloning vectors pPV-EF1a-EGxxFP-iP-A (Addgene ID: 204624), and pPV-EF1a-mRxxFP1(AfeI)-iP-A (Addgene ID: 204625).

DMD patient derived iPSC clones CiRA00646 (HPS3945) and CiRA00458 (HPS4305) are deposited in RIKEN BRC. MES-induced iPSC clones (CiRA00458 Δ45-55 #6–10, CiRA00646 Δ45-55 #9) will be deposited in RIKEN BRC https://cell.brc.riken.jp/.

### Ethical approval

The establishment and use of patient-derived iPSCs were approved by the Ethics Committee of the Graduate School of Medicine, Kyoto University, and Kyoto University Hospital (approval numbers R0091 and G259). All patient information was kept anonymous, and written informed consent was obtained.

### Transduction of CRISPR-Cas3 and CRISPR-Cas9

For transduction of iPSCs, 3 × 10^5^ cells were seeded in a well of a 12-well plate coated with iMatrix-511 silk. After 24 h, the cells were transfected with 1,000 ng of CRISPR-Cas3 expression plasmid (pPV-Dual_promoter-EF1α-2xNLS-Cascade+Cas3-iP [RD]) and 500 ng of crRNA expression plasmids (pBSIIKS-U6 or pPV-U6 vectors) by 4 μL of Lipofectamine Stem (Thermo Fisher Scientific). When applying the SSA reporter selection, we added 500 ng of SSA plasmid vectors. 1 day after the transfection, the culture medium was replaced with a fresh medium containing 1 μg/mL of puromycin. After selection with puromycin for 2 days, the cells were harvested for genomic DNA extraction or FACS (fluorescence-activated cell sorting) analysis.

For transduction of HEK293T cells, 2 × 10^5^ cells were seeded in a well of a 12-well plate. The next day, the cells were transfected with plasmid vectors using Lipofectamine 2000 (Thermo Fisher Scientific).

### SSA reporter-mediated cell sorting

The iPSCs transfected with the SSA reporter vectors were detached from a culture plate by incubating with 0.5 × TrypLE Select for 10 min at 37°C and then dissociated with PBS containing 2% FBS and 10 μM Y-27632. Similarly, HEK293T cells were detached from a plate by 0.25% Trypsin for 1 min at 37°C and dissociated with PBS containing 2% FBS. The cell suspension was passed through a 45-μm-pore cell strainer (Becton, Dickinson and Company) to remove cell clumps. Then, the fluorescence signals of EGFP and mRFP were detected by a BD FACSAria II, and the data were analyzed by FlowJo software (Becton, Dickinson and Company).

To isolate subclones of iPSCs with successful genome editing, we performed single-cell sorting into a well of a 96-well plate (pre-coated with iMatrix-511 silk) by using BD FACSAria II (BD) cell sorter. The sorted cells were cultured in StemFit AK02N medium containing Y-27632 for approximately 2 weeks. Then, single iPSC colonies were transferred into a new 24-well plate coated with iMatrix-511 silk. After reaching semi-confluency, half of the cells were cryo-preserved, and the other half were harvested for genomic DNA extraction. The target region was PCR amplified for genotyping using primers in Table S3. Sanger sequencing was performed to analyze deletion patterns.

### Quantification of genomic deletion by ddPCR assays

To detect the copy number loss of the genomic DNA, we designed ddPCR probes and primer pairs using Primer3Plus (https://www.bioinformatics.nl/cgi-bin/primer3plus/primer3plus.cgi) software. To detect the target genomic region (i.e., *dystrophin* exon 44–55 locus), we used TaqMan hydrolysis probes labeled with FAM fluorophores. Seven target probes were designed to be approximately equally spaced (every 57 kb) in the exon 45–55 region. Reference probes labeled with HEX fluorophores and primers were designed at the *dystrophin* exon 7 region, which is 160 kb distal from the nearest Cas3/Cas9 target site. All the primer pairs and probes are shown in Table S2.

Isolated genomic DNA (1 μg) was first digested by the EcoRI restriction enzyme (TaKaRa) with H buffer at 37°C overnight. Next, ddPCR reaction mixes (total 22 μL) were prepared by mixing 11 μL of Supermix for the Probes (no dUTP) (Bio-Rad Laboratories), 1 μL of 20 μM target forward and reverse primer pair each, and 0.6 μL of 10 μM target probe, 1 μL of 20 μM reference forward and reverse primer pair each, and 0.6 μL of 10 μM reference probe and 132 ng of EcoRI-digested genomic DNA, and the total volume was adjusted to 22 μL by ultrapure water. Oil droplets were generated using QX200 Automated Droplet Generator (Bio-Rad Laboratories) and run on a C1000 Thermal Cycler with a deep-well block (Bio-Rad Laboratories). All ddPCR reactions were run under the following three-step thermal conditions: step 1, 95°C for 10 min; step 2, 94°C for 30 s; step 3, 60°C for 2 min; repeat steps 2 and 3 for 40 times; step 4, 98°C for 10 min; and step 5, store at 4°C. After PCR reactions, droplets were analyzed by the Bio-Rad QX200 Droplet Reader and QuantaSoft Pro Software.

### Statistical analysis

To compare between two groups, two-tailed, unpaired Student’s t tests were performed using Prism 8 software. To compare among three groups or more, multiple t tests and Tukey’s tests were performed by using Prism 8 software. Fisher’s exact test was performed by using Prism 8 software.

## Data Availability

Data can be requested from the corresponding author. The raw human sequencing data or patient data may be restricted due to the legal restrictions in Japan and the consent of the patients.

## Supplemental information

Document S1. Figures S1–S4, Tables S1–S4, and supplemental experimental procedures

Document S2. Article plus supplemental information
