# Supplementary material for: Dual CRISPR-Cas3 system for inducing multi-exon skipping in DMD patient-derived iPSCs
Source: Stem Cell Reports. 2023 Aug 24;18(9):1753–65. doi: 10.1016/j.stemcr.2023.07.007 (PMC10545483; doi:10.1016/j.stemcr.2023.07.007)
Supplement: Document S1. Figures S1–S4, Tables S1–S4, and supplemental experimental procedures [file mmc1.pdf]

Stem Cell Reports, Volume *18*

Supplemental Information

Dual CRISPR-Cas3 system for inducing multi-exon skipping in DMD patient-derived iPSCs

Yuto Kita, Yuya Okuzaki, Youichi Naoe, Joseph Lee, Uikyu Bang, Natsumi Okawa, Akane Ichiki, Tatsuya Jonouchi, Hidetoshi Sakurai, Yusuke Kojima, and Akitsu Hotta

Supplemental information

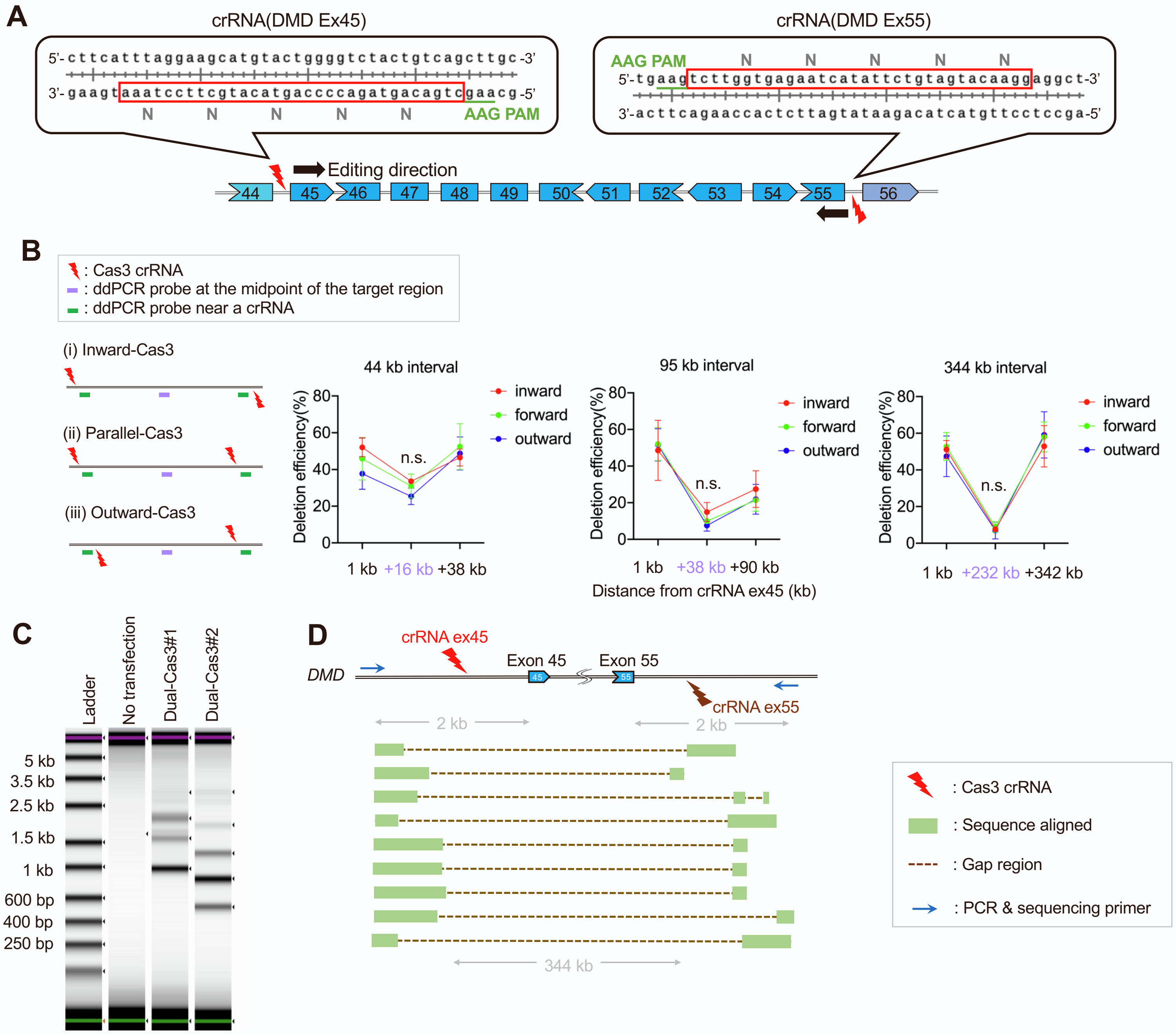

Supplemental Figure 1. Analyses and application of a large deletion by the dual-Cas3 system.
(A) The target sequences of crRNAs (DMD Ex45 and Ex55) used for the dual-Cas3 approach in Figure 1A (iv). CRISPR-Cas3 crRNA recognizes AAG (canonical PAM) + 32 nt target sequences, in which every 6 nt position is not involved in sequence recognition (indicated by "N").
(B) Comparison of the deletion efficiency among the inward, parallel, and outward orientation of dual crRNAs. Similar to Figure 1A (ii), (iii) and (iv), genome editing was performed in HEK293T cells using pairs of inward, parallel, and outward crRNAs. DNA copy number losses were measured by ddPCR at indicated positions. Data were represented as means ± SD from independent experiments (n = 3). ANOVA with the Tukey test was used to calculate *p*-values. n.s.: not significant.
(C) CRISPR-Cas3/Cascade expression vector with puromycin resistance cassette and two crRNA expression vectors targeting dystrophin exons 45 and 55 were transfected into DMD patient-derived iPSCs (FF12020). After one day of puromycin selection, the dystrophin exon 45-55 region was amplified by PCR and analyzed by TapeStation D5000 DNA tape.
(D) PCR products from (C) were subjected to Sanger sequencing after TA cloning. Sequencing results were aligned to the dystrophin gene locus shown as the green box. Identified deletion patterns with defined breakpoint(s) are indicated as the dotted line.

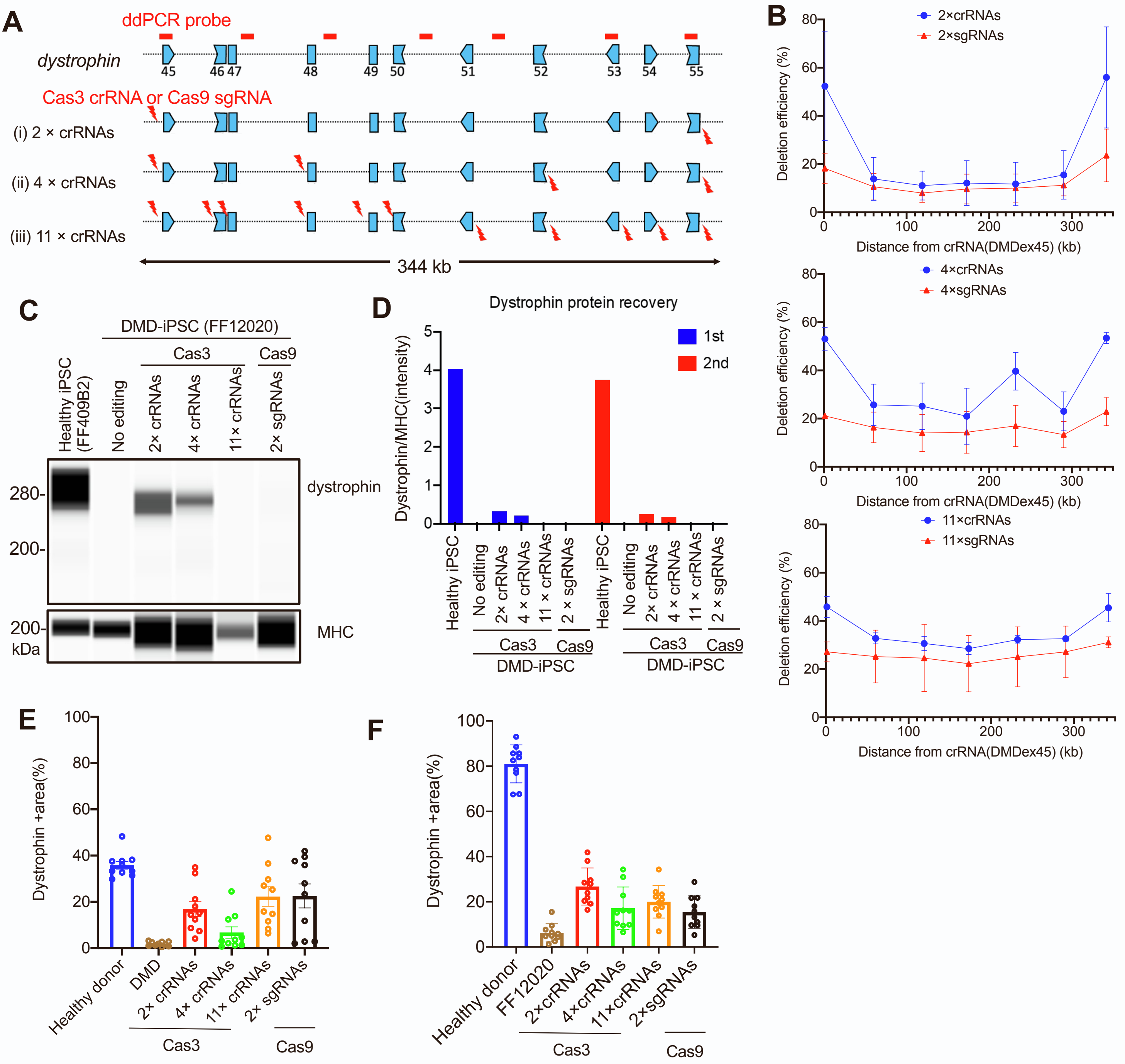

Supplemental Figure 2. Induction of multi-exon skipping by multiplexed crRNAs

(A) Schematic of designed Cas3 crRNAs/Cas9 sgRNAs and ddPCR probes for the DMD multi-exon skipping using multiplexed crRNAs. Positions of Cas3 crRNAs/Cas9 sgRNAs are shown as red thunder marks where the edge of the thunder mark indicates the PAM side. Positions of ddPCR probes are indicated as red horizontal bars.

(B) ddPCR analyses after multiplexed Cas3/Cas9 genome editing in HEK293T cells. DNA copy number losses were measured by ddPCR using probes indicated in (A). Data were represented as means ± S.D. from independent experiments (n = 3).

(C) Recovery of dystrophin protein after multiplexed Cas3 genome editing. Multiplexed Cas3 editing followed by two-color SSA vector enrichment for crRNA(Ex45) and crRNA(Ex55) was performed in FF12020 DMD-iPSC line. After skeletal muscle differentiation of the bulk cell samples, dystrophin and MHC (myosin heavy chain) proteins were detected by the Wes system. Data from two independent experiments are shown.

(D) Quantification of dystrophin protein amount normalized with MHC protein signal was calculated from the two independent Wes experiments.

(E, F) Immunocytochemical staining to assess recovery of dystrophin protein in FF12020 DMD-iPSC line. Genome-edited cells by multiplexed Cas3 were enriched with the two-color SSA vectors and subjected to skeletal muscle differentiation similar to (C). Immunocytochemical staining experiments for myosin heavy chain (MYH) and dystrophin proteins were performed and the results from two independent experiments are shown. For controls, healthy donor-derived iPSCs (FF409B2) and unedited parental FF12020 DMD-iPSC were used. Quantification of dystrophin-positive area within MHC-positive skeletal muscle cells. The area of the dystrophin-positive and the MHC-positive region were analyzed by ImageJ software. Data are represented as means ± S.D. from 10 images.

A

iPSC clone ID	Age at biopsy	Gender	DMD deletion analysis	Deletion pattern	Cell source
FF12020	8 y.o.	Male	MLPA	Exon 46, 47 deletion	Skin fibroblasts
CiRA00458	13 y.o.	Male	MLPA	Exon 51-53 deletion	Peripheral blood mononuclear cells
CiRA00646	20 y.o.	Male	MLPA	Exon 48-52 deletion	Skin fibroblasts

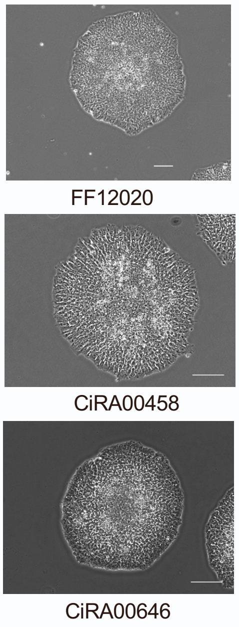

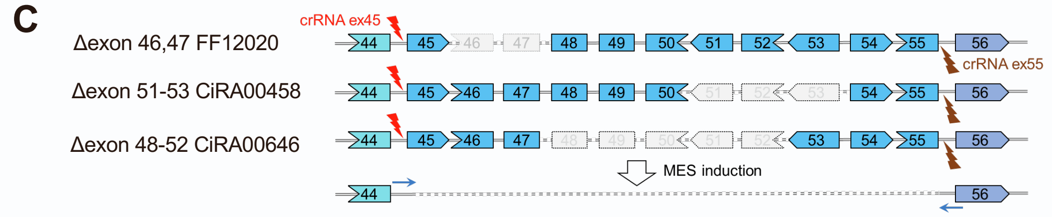

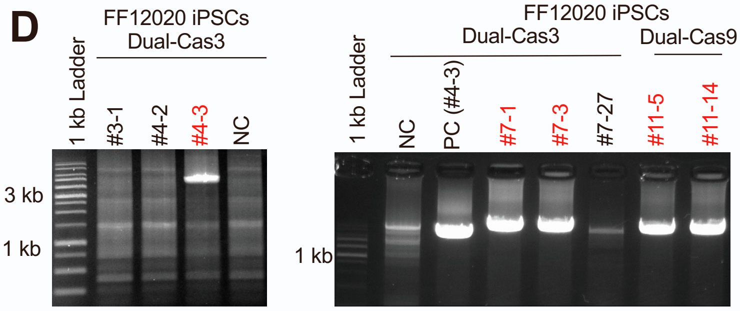

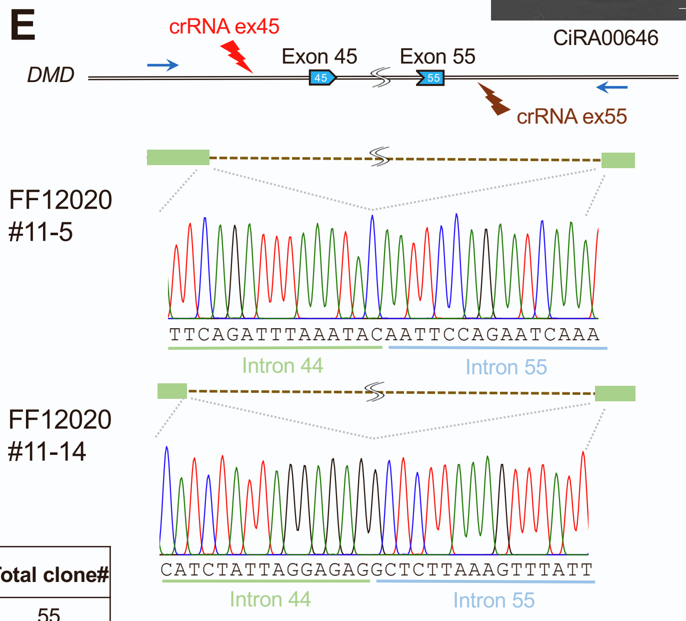

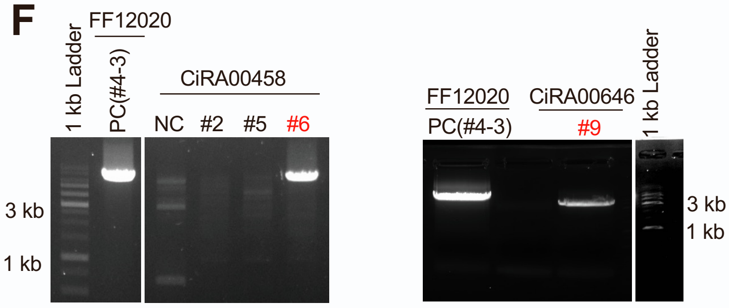

G

Summary of genotyping results

Cell line	SSA enrichment	Trial	Exon 45-55 large deletion	Total clone#
FF12020	No	1st trial	0 (0%)	55
		2nd trial	0 (0%)	65
	Yes	1st trial	1 (16.7%)	6
		2nd trial	2 (6.5%)	33
		3rd trial	18 (30.5%)	59
CiRA00458	Yes	1st trial	1 (33.3%)	3
CiRA00646	Yes	1st trial	1 (16.7%)	6

H

Summary of detailed genotyping for FF12020 3rd trial with SSA enrichment

Cell line	SSA enrichment	Trial	Exon 45-55 large deletion	Small deletions at Exon 45 & 55	Small deletion at only Exon 45	Small deletion at only Exon 55	Other	Unedited	Total clone#
FF12020	Yes	3rd trial	18 (30.5%)	4 (6.8%)	16 (27.1%)	1 (1.7%)	1 (1.7%)	19 (32.2%)	59

Supplemental Figure 3. Establishment of DMD patient-derived iPSCs and genotyping of subclones after MES induction by the dual-Cas3 system.
(A) DMD patients' information related to establishing the patient-derived iPSCs.
(B) Morphology of iPSC colony cultured on feeder-free and xeno-free culture condition. Scale bar : 100 μm.
(C) Exon deletion patterns of the three DMD patient iPSCs. For genotyping after dual-Cas3 treatment, we used the PCR primers that flank the 340 kb target region. PCR amplification happens only when a large deletion is induced.
(D) Genotyping was performed after dual-Cas3 treatment and subcloning in three DMD patient-derived iPS cell lines (FF12020, CiRA00458, and CiRA00646). As a result, we identified three subclones #4-3, #7-1, and #7-3 after dual-Cas3 treatment. and 2 subclones (#11-5, #11-14) after dual-Cas9 treatment.
(E) The deletion pattern of subclones #11-5, and #11-14 was analyzed by Sanger sequencing.
(F) Subcloning and genotyping were also performed in CiRA00458 and CiRA00646 DMD-iPSC lines and subclones #6 and #9 were established, respectively.
(G) Summary of single-cell cloning and genotyping. Dual-Cas3 genome editing was performed in FF12020, CiRA00458, and CiRA00646 iPSCs. Isolated subclones were genotyped by the amplification size of junction PCR to detect large Ex45-55 deletions indicated in (C) and (D).
(H) Detailed genotyping results in the 3rd subcloning trial of the FF12020 DMD-iPSCs with the SSA vector enrichment. PCR was performed on exon 45, exon 55, and intron 50 regions separately to various genome editing patterns.

A

CNV_ID	Position	crRNA binding site (PAM)	Mismatch (nt)	Strand	Distance (kb)
32	chr8:58,511,328-62,603,471	GTGCTGgCCtgAGAAgaCAaGACcaGCTTCCTCAA	8	-	908 & 3184

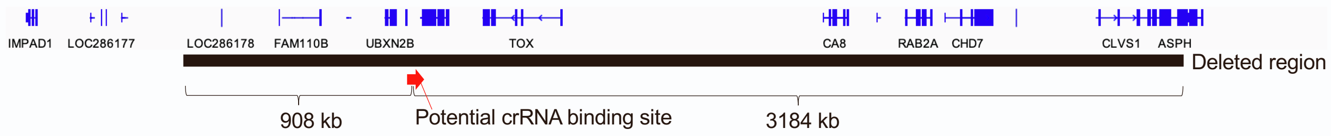

B

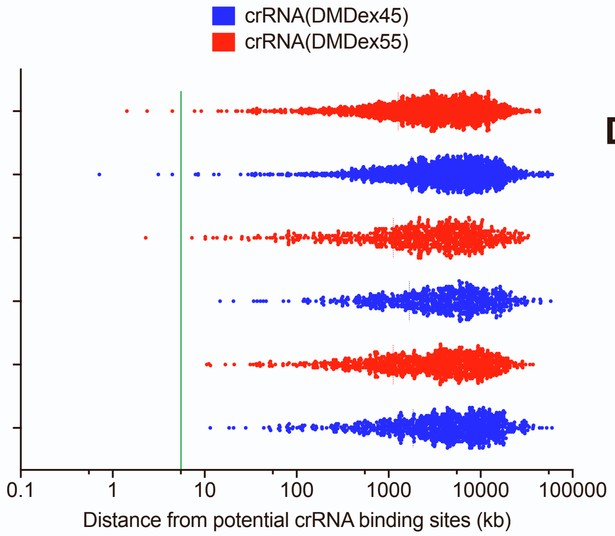

D

Sample ID	Total No. of Indels/SNVs detected	No. of Indels/SNVs detected within 5 kb from the potential crRNA binding sites	Fisher's exact test (relative to NC2)
NC2	997	0	
#7-1	717	1	$p = 0.42$
#4-3	2038	6	$p = 0.19$

C

#4-3	#7-1	Position	Ref	Alt	Nearest crRNA	crRNA binding site	Mismatch	Distance from the indels/SNVs	Exist in the Cas3 cleavage direction?
x	-	chr4:116904319	C	T	ex45	chr4:116903572_116903603	8 bp	0.716 kb	No
x	-	chr10:119402039	A	C	ex45	chr10:119398878_119398909	8 bp	3.127 kb	Yes
x	-	chr17:79314829	C	T	ex45	chr17:79310353_79310384	8 bp	4.445 kb	No
x	-	chr2:229669588	-	AG	ex55	chr2:229671019_229671050	8 bp	1.428 kb	Yes
-	x	chr:4175456696	G	T	ex55	chr4:175458986_175459017	8 bp	2.287 kb	Yes
x	-	chr15:95919559	C	A	ex55	chr15:95917160_95917191	8 bp	2.368 kb	No
x	-	chr17:36013899	T	-	ex55	chr17:36018355_36018386	7 bp	4.453 kb	No

Supplemental Figure 4. Additional information for the off-target analyses performed by whole genome sequencing.

(A) Detailed information of the 4058 kb CNV containing a potential crRNA (DMD ex45) binding site. In the putative crRNA binding site, the noncanonical PAM motif is shown underbar, and mismatch bases are shown as small letters.

(B) The overall distances between the detected indel/SNV and the closest potential crRNA binding site are plotted, where blue dots represent the distances from the potential binding sites of crRNA ex45, and red dots represent the distances from the crRNA ex55. The vertical green line represents a 5 kb window, where most of the Cas3-induced cleavage happens within 5 kb from the crRNA binding site.

(C) Indels and SNVs detected within a 5 kb window from the potential crRNA binding sites in two MES-induced clones #7-1 and #4-3.

(D) The number of SNV/indels detected in the whole genome and in the 5 kb window from potential crRNA binding sites are shown for the Cas3-untreated clone (NC2) and two MES-induced clones #7-1 and #4-3. By using NC2 as a control, Fisher's exact test was performed to calculate the odds of finding indels/SNVs in the given area (whole genome vs. ± 5 kb from crRNAs).

Supplemental Table 1. List of Cas3 crRNA/Cas9 sgRNA targets

Cas3 crRNA/ Cas9 sgRNA name	Alternative name	PAM	Target Sequence	Used in
crRNA(DMD Ex45)	crRNA(DMD#20)	AAG	ctgacagtagaccccagtacatgcttcctaaa	1-4, S1, S2, S4
crRNA(DMD Ex55)	crRNA(DMD#23)	AAG	tcttggtgagaatcatattctgtagtacaagg	1-4, S1, S2, S4
crRNA(DMD Ex45_1kb)	crRNA_DMDex45_1kb	AAG	Cctgaatctgcggtggcaggaggtctgcaaac	1, S1
crRNA(DMD Ex45_44kb)	crRNA_DMDex45_44kb	AAG	Ctcaagcagacaaatctccagtggataaaggt	1, S1
crRNA(DMD Ex45_95kb)	crRNA_DMDex45_95kb	AAG	acaaaatattacttgttaaagtgtggtaagga	1, S1
crRNA(DMD Ex45_outward)	crRNA_DMD#9	AAG	gaagacttggcctttatttaccaaatgagact	S1
crRNA(DMD Ex47)	crRNA(DMDex45_44kb_outward)	AAG	gaaactgagacaaaggggttaaataccagctc	S1, S2
crRNA(DMD Ex45_90kb_outward)	crRNA_DMDex45_95kb_out_1	AAG	ataagcaaaagtgtctgttttagaaataaaca	S1
crRNA(DMD Ex55_outward)	crRNA_DMDex55_out_1	AAG	aaaatacaaacctagaatcaaaaggaaaagac	S1
crRNA(B2M)	crRNA(B2M#1)	AAG	gccacggagcgagacatctcggcccgaatgct	2
crRNA(DMD Ex46)	crRNA(DMDexon46_1)	AAG	actccatctcaaatacatacatacatacgtac	S2
crRNA(DMD Ex48)	crRNA(DMDexon48_1)	AAG	attgtaaaaggaagtcccttcatgtacaagga	S2
crRNA(DMD Ex49)	crRNA(DMDexon49_1)	AAG	cttattatttattaatgagtcaaacaggcctt	S2
crRNA(DMD Ex50)	crRNA(DMDexon50_1)	AAG	ttcatgcctttccccaggcagccctcattcag	S2
crRNA(DMD Ex51)	crRNA_DMD#25	AAG	ttggcatttatgcaatgccatgttcaaatgaa	S2
crRNA(DMD Ex52)	crRNA(DMDexon52_1)	AAG	acccatctgactagacgctgtgcatattcttt	S2
crRNA(DMD Ex53)	crRNA(DMDexon53_1)	AAG	atcacttcaaaattggtatacgtatttatgta	S2
crRNA(DMD Ex54)	crRNA(DMDexon54_1)	AAG	ccttgtatccttattatacctctcttgatctc	S2
sgRNA(DMD Ex45#3)	sgRNA_DMDexon45#3	NGG	ttgctattgtgtcaaggagt	1, S4
sgRNA(DMD Ex55#1)	sgRNA(DMDexon55#1)	NGG	tcatattctgtagtacaagg	1, S4
sgRNA(DMD Ex45_1kb)	sgRNA_DMD1	NGG	tggtatcttacaggaactcc	1
sgRNA(DMD Ex45_44kb)	sgRNA_DMDex45_44kb	NGG	agaagcaaagacaaggtagt	1
sgRNA(DMD Ex45_95kb)	sgRNA_DMDex45_95kb	NGG	aaatattacttgttaaagtg	1
sgRNA(DMD Ex46)	sgRNA_DMDex46_1	NGG	agggtactggttatacttgg	S2
sgRNA(DMD Ex47)	sgRNA_DMDex47_2	NGG	gggatatgaagtaaaaaatg	S2
sgRNA(DMD Ex48)	sgRNA_DMDex48_2	NGG	aaacttccaaggatgatggt	S2
sgRNA(DMD Ex49)	sgRNA_DMDex49_2	NGG	tgctttaagtgtttaccctt	S2
sgRNA(DMD Ex50)	sgRNA_DMDex50_2	NGG	cagctcaggcagtccctgaa	S2
sgRNA(DMD Ex51)	sgRNA_DMDex51_2	NGG	agataaacttgggctcaggt	S2
sgRNA(DMD Ex52)	sgRNA_DMDex52_1	NGG	gagaatctctgagggaaacc	S2
sgRNA(DMD Ex53)	sgRNA_DMDex53_1	NGG	aggaaggaattaagcccgaa	S2
sgRNA(DMD Ex54)	sgRNA_DMDex54_2	NGG	tagctccctattatatcacg	S2

Supplemental Table 2. Primers used for PCR, Sanger sequencing, and RT-PCR (left column). Primers and probes used for ddPCR experiments (Right column). Related to Experimental Procedures section.

Name	Sequence
YK#87_crRNA_DMDexon45check_rev	acatctgatgtgtgcccatgc
YK#88_crRNA_DMDexon55check_dir	cgcaaatgttgaggttttcagaggc
YK#89_crRNA_DMDexon55check_rev	ggatttggctgaaaatcacattgtctcg
YK#90_crRNA_DMDexon51check_dir	gaggctataaaagccaagaactgacaatg
YK#91_crRNA_DMDexon51check_rev	catttgccagtcagcctggtgttctgc
YK#92_crRNA_DMDexon45check_rev2	ctatggtatacatctgatgtgtgcccatgc
YK#93_crRNA_DMDexon45check_rev3	cttctggcctaccatttgttgatcacc
YK#107_DMDexon45-55check_dir1	cctaccatagctgatgggtaaaatgtaaac
YK#108_DMDexon45-55check_dir2	gaagcttgcagtttcttgagattagaaatagag
YK#109_DMDexon45-55check_rev1	gactggtgtctctattactaagcaatgactgtc
YK#110_DMDexon45-55check_rev2	gcaaatgcttactgaaaccttccatgcatc
YK#113_DMDexon45(45-55)check_rev3	gactccatggtgatgatgagcc
YK#114_DMDexon45(45-55)check_rev4	ctcctccgctcatccttttcactg
YK#115_DMDexon45(45-55)check_rev5	caccacaggctttaacttctgccg
YK#116_DMDexon55(45-55)check_dir3	ggttaagccactggacaaaaacg
YK#117_DMDexon55(45-55)check_dir4	gagtagcctgatcgacactggac
YK#118_DMDexon55(45-55)check_dir5	cctcgggtacactgaaagttatgtg
YK#167_sgRNA(44C4)_check_dir	gccaacgctggtgtgcatgc
YK#168_sgRNA(44C4)_check_rev	gcctggacggagctggtttatc
YK#169_sgRNA(55C3)_check_dir	cttggcgtattgccttctcctg
YK#170_sgRNA(55C3)_check_rev	gttcctcccacatccacacgc
YK#171_sgRNA(DMDexon45)_check_dir	caggagcatcccatcaagatatccc
YK#172_sgRNA(DMDexon55)_check_dir	ctcaacacgcattttggaggag
YK#173_sgRNA(DMDexon55)_check_rev	gctctgaccaggaaaacatttgc
YK#229_crRNA(DMDexon46)_check_dir	gcagaactgctcaactgagcc
YK#230_crRNA(DMDexon46)_check_rev	gccactgagctggacacacg
YK#231_crRNA(DMDexon47)_check_dir	ccctagaacaatgaactcagtc
YK#232_crRNA(DMDexon47)_check_rev	cccactctaacctttggatgaaggg
YK#233_crRNA(DMDexon48)_check_dir	gctctaaggtatgttcagggatgacg
YK#234_crRNA(DMDexon48)_check_rev	cacacagcagtgagtggtgac
YK#235_crRNA(DMDexon49)_check_dir	gcaagagactgtactgggcag
YK#236_crRNA(DMDexon49)_check_rev	gcttgggaggtgagcgtagg
YK#237_crRNA(DMDexon50)_check_dir	ctgccctagggcgttaggac
YK#238_crRNA(DMDexon50)_check_rev	cctgcaagtcaggaagtcggg
YK#239_crRNA(DMDexon52)_check_dir	cgttactctcaccatatgcttgtg
YK#240_crRNA(DMDexon52)_check_rev	gtgcacacaccatctaatgcttatg
YK#241_crRNA(DMDexon53)_check_dir	gctgttccactacctagtggaac
YK#242_crRNA(DMDexon53)_check_rev	gagggttttgatcaagtgccc
YK#243_crRNA(DMDexon54)_check_dir	gagtgatgggtggcttccaag
YK#244_crRNA(DMDexon54)_check_rev	gagaagaaagtccaagccttgcc
YK#297_DMDexon55(45-55)check_dir6	cctgaagtacaaggacgacgg
YK#298_DMDexon55(45-55)check_dir7	cttcagaattaatccgtgcgcc
YK#299_DMDexon55(45-55)check_dir8	cacacacaaaccaccgaaccaaag
YK#300_DMDexon45(45-55)check_rev6	gtagacttagaatggaattctgggc
YK#301_DMDexon45(45-55)check_rev7	gaatgggtcctggtgctgtgcttagc
YK#302_DMDexon45(45-55)check_rev8	ccgcggtatctgttgttcgc
YK414_DMDex55_check_F	cctcccacaaaacatatgacagtg
YK415_DMDex45_check_R	catgaatgagttagaaggcgagaagg
YK416_DMDex45_check_rev10	ggagtcagtcacactaaagagaacac
YK417_DMDex55_check_dir10	gcaagaagacaatcatgtgagtcttg
YK573_DMDex45_40kb_check_F	cagcagcaagctacggtatg
YK574_DMDex45_95kb_check_F	ggtttccagagctttacctgaga
YK580_crRNA_DMDex45_95kb_check_F	ccctgctgccatgtagaatgtg
YK581_crRNA_DMDex45_95kb_check_R	ctcctacactgctccttggc
YK582_sgRNA_DMDex45_40kb_check_R	cacatgtgacggaagagatgg
YK188_DMDexon56_rev2	caggactgcatcatcggaacc
YK378_DMD_E44_cDNA_F	cagtggctaacagaagctgaac
YK379_DMD_E45_cDNA_R	ggcatctgtttttgaggattgc

Name	Sequence
DMDexon7_ddPCR_F	tgtgttttaggccagacc
DMDexon7_ddPCR_R	agcagtggtagtccagaa
DMDexon7_probe	agtcgttgtgtggctgactgctggca
DMDex45_ddPCR_F	ggaactccaggatggcattgg
DMDex45_ddPCR_R	gacagctgtttgcagacct
DMDexon45_probe	tgggaagcctgaatctgcggtggca
DMDin50_ddPCR_F	ccccaggaagggcattaag
DMDin50_ddPCR_R	agttcagatgttgtcccc
DMDin50_probe	cccccatgaccccacacttcccgt
DMDin47_ddPCR_F	ctgacttccctcttggtgac
DMDin47_ddPCR_R	caggaatgagcatgtgacct
DMDintron47_probe	tggcctgaagccgtttcagaacccaca
DMDin48_ddPCR_F	tgcagctttactccacac
DMDin48_ddPCR_R	ggcagttcaagatttgctgg
DMDintron48_probe	cccaggaccctagcggaaagagcagc
DMDin51_ddPCR_F	ctgttgctggatactttcgc
DMDin51_ddPCR_R	cgactcactctcctctcat
DMDintron51_probe	cccaggaccctagcggaaagagcagc
DMDex53_ddPCR_F	caatggctggaagctaagga
DMDex53_ddPCR_R	tgtatagggaccctccttcc
DMDexon53_probe	agcaggtcttaggacaggccagagcca
DMDex55_ddPCR_F	ggctgctttggaagaaactc
DMDex55_ddPCR_R	cagctcttttactcccttgg
DMDexon55_probe	actgcaacagttccccctggacctgga
ddPCR_ex45_16kb_fwd	cccagcttggtcaagcataa
ddPCR_ex45_16kb_rev	agactgcaactttaggccag
ddPCR_ex45_16kb_probe	tcagtaacggtggagacctaggcggca
ddPCR_ex45_38kb_fwd	tcaccacttcagcctctact
ddPCR_ex45_38kb_rev	aggacggtatgaaaagccac
ddPCR_ex45_38kb_probe	accaacaccacgtcccacaccccc
ddPCR_ex45_90kb_fwd	tggtaggaagatgagctggt
ddPCR_ex45_90kb_rev	agtatgggggatcctcttcc
ddPCR_ex45_90kb_probe	ccccacatgtcaagggtggagccagg
ddPCR_ex45_Neg1kb_fwd	acgttgcctcaagttctctg
ddPCR_ex45_Neg1kb_rev	agtgaaaaggatgagcggag
ddPCR_ex45_Neg1kb_probe	cccaggccccaatatacaccgagggc

Supplemental Table 3. List of CNVs detected in WGS. Related to Figure 4.

ID	Type	Location	Size (bp)	NC2	#4-3	#7-1
1	loss	chr1:838,617-1,030,456	191,840	-	○	-
2	gain	chr1:144,810,826-145,298,712	487,887	-	○	-
3	loss	chr2:1,357,914-1,358,836	923	○	-	○
4	loss	chr2:37,258,016-37,278,680	20,665	-	○	-
5	loss	chr2:242,790,469-242,790,961	493	-	-	○
6	loss	chr3:3,412,335-3,412,629	295	○	-	-
7	loss	chr3:4,848,820-4,848,876	57	-	-	○
8	loss	chr3:45,609,905-45,610,321	417	-	-	○
9	loss	chr3:194,384,128-194,384,520	393	-	-	○
10	gain	chr4:54,592,280-54,603,331	11,052	-	○	-
11	loss	chr4:65,719,085-65,719,575	491	-	-	○
12	gain	chr4:92,606,746-93,528,071	921,326	-	○	-
13	loss	chr5:2,748,121-2,760,235	12,115	-	○	-
14	loss	chr5:46,043,702-46,086,649	42,948	-	○	-
15	loss	chr5:46,218,649-46,333,576	114,928	-	○	-
16	loss	chr5:46,333,576-49,443,137	3,109,562	-	○	-
17	gain	chr5:178,012,401-178,012,673	273	-	○	-
18	gain	chr5:180,566,548-180,566,851	304	-	-	○
19	loss	chr6:217,664-382,715	165,052	-	○	-
20	loss	chr6:382,715-3,367,820	2,985,106	-	○	-
21	loss	chr6:29,815,110-29,815,768	659	-	-	○
22	loss	chr6:94,248,198-94,264,157	15,960	-	○	-
23	loss	chr6:141,304,606-141,318,406	13,801	-	○	-
24	gain	chr6:169,240,479-169,240,928	450	-	-	○
25	gain	chr6:169,331,272-169,331,585	314	-	-	○
26	loss	chr7:57,950,847-58,013,552	62,706	-	○	-
27	loss	chr7:61,241,479-61,511,524	270,046	-	○	-
28	loss	chr7:150,463,048-150,463,551	504	○	-	-
29	gain	chr8:20,198,662-20,203,119	4,458	-	○	-
30	loss	chr8:20,198,767-20,203,952	5,186	-	○	-
31	loss	chr8:43,597,003-43,660,505	63,503	-	○	-
32	loss	chr8:58,511,328-62,603,471	4,092,144	-	-	○
33	loss	chr8:91,254,169-91,903,496	649,328	○	-	-
34	loss	chr8:103,441,499-103,441,868	370	-	-	○
35	loss	chr8:123,165,142-123,168,985	3,844	○	-	-
36	loss	chr9:83,269,037-83,271,232	2,196	-	○	-
37	loss	chr10:8,075,816-8,108,417	32,602	-	○	-
38	loss	chr10:47,642,581-47,643,078	498	-	-	○
39	loss	chr10:55,893,688-55,894,134	447	-	-	○
40	loss	chr10:67,032,382-67,032,923	542	○	-	-
41	gain	chr10:134,879,786-134,880,359	574	-	○	-
42	gain	chr11:48,528,012-87,356,743	38,828,732	-	○	-
43	loss	chr11:50,781,651-51,163,742	382,092	-	○	-
44	loss	chr11:51,207,530-51,252,364	44,835	-	○	-
45	loss	chr11:63,527,207-63,540,475	13,269	-	○	-
46	loss	chr12:38,231,554-38,408,805	177,252	-	○	-
47	gain	chr12:132,879,982-132,880,445	464	-	○	-
48	loss	chr13:95,355,676-95,368,107	12,432	-	○	-
49	loss	chr13:110,432,870-110,446,468	13,599	-	○	-
50	loss	chr14:46,590,901-46,621,369	30,469	-	○	-
51	loss	chr14:78,827,811-78,828,585	775	-	-	○
52	loss	chr14:87,857,880-87,858,384	505	-	-	○
53	loss	chr14:104,674,521-104,675,002	482	○	-	-
54	loss	chr15:74,653,286-75,221,457	568,172	-	○	-
55	loss	chr15:74,653,679-74,709,590	55,912	-	○	-
56	gain	chr15:75,221,643-82,903,355	7,681,713	△	○	-
57	gain	chr15:82,903,355-83,167,067	263,713	△	○	-
58	gain	chr15:83,167,067-100,457,023	17,289,957	△	○	-
59	gain	chr15:100,457,023-102,445,015	1,987,993	△	○	-
60	gain	chr16:12,021,477-29,394,630	17,373,154	-	○	-
61	loss	chr16:29,815,073-29,828,813	13,741	-	○	-
62	loss	chr16:71,407,745-71,439,679	31,935	-	○	-
63	loss	chr16:85,639,482-85,652,218	12,737	-	○	-
64	loss	chr16:88,024,857-88,024,916	60	-	○	-
65	loss	chr17:77,804,119-77,821,079	16,961	-	○	-
66	gain	chr19:22,363,444-22,363,870	427	-	○	-
67	loss	chr19:27,833,052-27,870,223	37,172	-	○	-
68	loss	chr19:27,893,812-28,065,242	171,431	-	○	-
69	loss	chr20:20,337,172-20,338,425	1,254	-	○	-
70	gain	chr20:25,582,240-25,582,500	261	-	○	-
71	gain	chr20:29,836,152-30,788,715	952,564	△	○	-
72	loss	chr20:41,624,640-41,634,640	10,001	○	-	-
73	loss	chr20:50,347,622-50,348,035	414	-	○	-
74	loss	chr20:52,318,479-52,318,783	305	-	-	○
75	gain	chr20:55,062,013-55,385,592	323,580	○	-	-
76	loss	chr22:50,305,756-50,418,937	113,182	-	○	-
77	loss	chrX:31,643,677-31,989,563	345,887	-	○	○
78	loss	chrX:50,122,314-50,135,051	12,738	○	-	-
79	loss	chrX:72,358,023-72,375,780	17,758	-	○	-
80	loss	chrX:80,280,719-80,295,147	14,429	-	-	○
81	loss	chrX:112,703,477-112,716,769	13,293	○	-	-
82	loss	chrX:129,369,525-129,383,770	14,246	○	-	-
83	loss	chrX:148,700,413-148,715,061	14,649	○	-	-
84	loss	chrY:13,637,403-13,868,434	231,032	-	○	-
85	loss	chrY:22,224,764-22,486,672	261,909	-	○	-

○ : CNV is detected
— : CNV is not detected
△ : CNV is a possible mosaic gain

Supplemental Table 4. List of SNV/indels detected at exonic region in WGS. Related to Figure 4.

NC2		#4-3		#7-1									Origin		NC2		#4-3		#7-1	
Genomon	Genomon2	Genomon	Genomon2	Genomon	Genomon2	Chr	Start	End	Ref	Alt	Func.refGene	Gene.refGene	coverage	alt_ratio	coverage	alt_ratio	coverage	alt_ratio	coverage	alt_ratio
○	○	○	○	-	-	chr8	145,254,085	145,254,085	C	T	exonic	MROH1	72	0.0%	44	18.2%	34	29.4%	47	0.0%
○	○	-	-	-	-	chr13	73,301,740	73,301,740	T	G	exonic	MZT1	40	0.0%	41	41.5%	43	0.0%	36	2.8%
○	○	-	-	-	-	chr20	3,673,216	3,673,216	C	T	exonic	SIGLEC1	60	0.0%	44	29.6%	47	0.0%	42	0.0%
○	-	-	-	-	-	chr2	125,192,138	125,192,138	G	T	exonic	CNTNAP5	51	0.0%	38	21.1%	54	0.0%	38	2.6%
-	○	○	○	-	-	chr14	31,771,711	31,771,711	G	T	exonic	HEATR5A	30	0.0%	33	15.2%	50	60.0%	35	0.0%
-	-	○	○	-	-	chr3	42,916,713	42,916,713	G	T	exonic	CYP8B1	54	0.0%	41	7.3%	46	54.4%	33	0.0%
-	-	○	○	-	-	chr3	65,428,513	65,428,513	C	A	exonic	MAGI1	41	0.0%	45	0.0%	43	58.1%	24	0.0%
-	-	○	○	-	-	chr3	194,118,222	194,118,222	G	A	exonic	GP5	46	0.0%	46	13.0%	52	46.2%	36	0.0%
-	-	○	○	-	-	chr4	16,035,028	16,035,038	ACAGCAACGAC	-	exonic	PROM1	52	0.0%	32	0.0%	53	45.3%	35	0.0%
-	-	○	○	-	-	chr5	179,545,801	179,545,801	A	T	exonic	RASGEF1C	60	0.0%	49	0.0%	64	56.3%	45	0.0%
-	-	○	○	-	-	chr6	46,679,244	46,679,244	G	A	exonic	PLA2G7	52	0.0%	42	0.0%	58	48.3%	34	0.0%
-	-	○	○	-	-	chr6	99,956,659	99,956,659	C	T	splicing	USP45	64	0.0%	58	10.3%	49	34.7%	38	0.0%
-	-	○	○	-	-	chr6	150,059,867	150,059,867	G	A	exonic	NUP43	41	0.0%	40	10.0%	49	57.1%	51	0.0%
-	-	○	○	-	-	chr6	151,646,998	151,646,998	G	A	exonic	AKAP12	38	0.0%	37	10.8%	50	44.0%	38	0.0%
-	-	○	○	-	-	chr8	2,048,819	2,048,819	C	T	exonic	MYOM2	48	0.0%	46	0.0%	39	41.0%	41	0.0%
-	-	○	○	-	-	chr11	49,078,765	49,078,765	C	A	exonic	TRIM64C	37	0.0%	46	10.9%	40	32.5%	36	0.0%
-	-	○	○	-	-	chr11	114,393,013	114,393,013	G	T	exonic	NXPE1	42	0.0%	42	7.1%	44	59.1%	27	0.0%
-	-	○	○	-	-	chr17	61,417,487	61,417,487	C	A	exonic	TANC2	48	0.0%	23	0.0%	50	44.0%	36	0.0%
-	-	○	○	-	-	chr19	15,905,528	15,905,528	G	A	exonic	OR10H5	44	0.0%	50	8.0%	41	46.3%	35	0.0%
-	-	○	○	-	-	chr19	46,257,754	46,257,754	C	T	exonic	BHMG1	53	0.0%	50	0.0%	45	51.1%	41	0.0%
-	-	○	○	-	-	chr19	48,547,154	48,547,154	C	T	exonic	CABP5	43	0.0%	38	5.3%	47	61.7%	39	0.0%
-	-	-	-	○	○	chr1	10,336,388	10,336,388	C	T	exonic	KIF1B	49	0.0%	36	0.0%	54	0.0%	35	42.9%
-	-	-	-	○	○	chr5	64,887,329	64,887,329	A	G	exonic	TRIM23	32	0.0%	39	0.0%	39	0.0%	36	44.4%
-	-	-	-	○	○	chr8	43,147,743	43,147,743	G	A	exonic	POTEA	34	0.0%	28	0.0%	33	0.0%	25	36.0%
-	-	-	-	○	○	chr14	68,040,102	68,040,102	C	T	exonic	PLEKHH1	57	0.0%	36	0.0%	55	0.0%	49	40.8%
-	-	-	-	○	○	chr18	76,755,107	76,755,110	CCAA	-	exonic	SALL3	47	0.0%	54	0.0%	47	0.0%	45	57.8%
-	-	-	-	○	○	chrX	129,146,629	129,146,629	C	A	exonic	BCORL1	31	0.0%	27	0.0%	23	0.0%	22	100.0%

○ : SNV/indel is detected
ー : SNV/indel is not detected
Genomon : a sequence analyzing tool to detect genomic variants
Genomon2 : an updated version of Genomon
Coverage: the depth of coverage at the target site
Alt ratio: a percentage of detected SNVs

Supplemental Experimental procedures

Vector construction

The polycistronic CRISPR-Cas3 vectors pPV-Dual_promoter-EF1α-2xNLS-Cascade+Cas3-iP (RD) (Addgene ID: 204619) were constructed in the previous study (Morisaka *et al*, 2019). To construct Cas3 all-in-one vector with mCherry (pPV-Dual_promoter-EF1α-2xNLS-Cascade+Cas3-iCA (RD)), the 2xNLS-Cas7-Cas5-Cas8-IRES-mCherry region was PCR amplified from the pPV-EF1α-2xNLS-Cas7-Cas5-Cas8-iCA vector (Addgene ID: 134922) by KOD ONE (TOYOBO) and purified with Wizard SV Gel and PCR Clean-Up System (Promega). The PCR product was inserted into the HindIII site of the pPV-EF1α-2xNLS-Cas11-Cas6-Cas3-A vector (Addgene ID: 134923) by In-Fusion reaction (Clontech).

To construct Cas3-crRNA and Cas9-sgRNA expression vectors, the synthesized crRNA/sgRNA sequences (Hokkaido System Science) were inserted into the two BbsI sites immediately downstream of the U6 promoter (pBSIIKS-U6v2-BbsI-C1, pBSIIKS-U6-BbsI-Cas9 or pPV-U6-crRNA-cloning-EF1α-BA (Addgene ID: 204623)). As for the crRNA/sgRNA sequences, Annealed oligonucleotides (Sense: "ACCG" 4 nt overhang + 32 nt target sequence (top strand), Antisense: "ACAC" 4 nt overhang + 32 nt target sequence (bottom strand) for crRNA, Sense: "ACCG" 4 nt overhang + 32 nt target sequence (top strand), Antisense: AAAC+ 32 nt target sequence (bottom strand) for sgRNA) were inserted between BbsI sites into the backbone vectors for ligation reaction. To design Cas9 sgRNA target sites, CRISPick (https://portals.broadinstitute.org/gppx/crispick/public) was used. To design Cas3 crRNA target sites, AAG (PAM) + 32 nt sequences (Cas3 induces deletions toward PAM direction) were extracted, and GGGenome (https://gggenome.dbcls.jp) was used to check their specificity to the human genome. Examples of designed crRNAs are shown in Figure S1A. Cas3-crRNA targeting DMD exon 45 and 55 are deposited into Addgene (pPV-C1-crRNA (DMD#20_DMD#23)-EF1a-BA (Addgene ID: 204620)). All the target sequences are shown in Supplemental Table 1.

To construct single-strand annealing (SSA) vectors, the genomic sequences around the *dystrophin* exon 45 or exon 55 region containing crRNA or sgRNA target site were amplified by PCR. The amplified fragments were inserted into the AfeI site of the pPV-EF1α-eGxxFP-iBA or pPV-EF1α-mRxxFP-iPA (Addgene ID: 204625) vector by In-Fusion reaction. SSA vectors with 1.0 kb spacer were deposited into Addgene (pPV-EF1α-EGxxFP(DMDex45_10)-iP-A(Addgene ID: 204621) and pPV-EF1α-mRxxFP1(DMDex55_10)-iP-A (Addgene ID: 204622)).

To construct double-nick SSA vectors, single strand oligos containing two CRISPR target sites and a spacer (35 bp, 25 bp, or 15 bp) were annealed and amplified into double-strand DNA by PCR. The PCR fragments were inserted into the AfeI site of the pPV-EF1a-eGxxFP-iPA (Addgene ID: 204624) vector by an In-Fusion reaction.

To construct a double-nick EGFP SSA vector (EGxxFP) with 0 bp spacer, the pPV-EF1a-eGxxFP-iPA vector backbone region was amplified by PCR, and the target region oligos were cloned by In-Fusion reaction.

To construct a double-nick mRFP SSA vector (mRxxFP) with 0 bp spacer, pPV-EF1a-mRxxFP-iPA vector was digested with AfeI and the target region oligos were cloned by In-Fusion reaction.

Cell culture

HEK293T cells (CRL-3216, ATCC) were cultured in Dulbecco’s modified Eagle’s medium (DMEM) high glucose (Nacalai Tesque, Kyoto, Japan) supplemented with 10% FBS (BioSera North America, Kansas City, MO, USA). All iPSCs were cultured in

StemFit AK02N media (Ajinomoto, Tokyo, Japan) on iMatrix-511 silk (Matrixome, Osaka, Japan) coated cell culture dishes.

Establishment of human iPSCs

Healthy donor iPS cell line FF409B2 (Okita *et al*, 2011) and DMD patient-derived iPS cell line FF12020 lacking exon 46 and 47 (Uchimura *et al*, 2021) were established previously. To establish other DMD patient-derived iPSC lines, fibroblasts (exon 51-53 deletion) or the PBMCs (exon 48-52 deletion) were electroporated with the three episomal DNA vectors encoding OCT3/4, SOX2, KLF4, L-MYC, LIN28, and p53 carboxy-terminal dominant-negative fragment (mp53DD) cDNAs, as previously described (Hong *et al*, 2009; Okita *et al.*, 2011). Each patient-derived iPSC line was registered as CiRA00458 (exon 51-53 deletion) and CiRA00646 (exon 48-52 deletion), respectively.

PCR-based genotyping and TA cloning

To examine Cas3's deletion patterns, PCR was performed using QuickTaq (TOYOBO). In this PCR, the forward primer was designed at the dystrophin exon 45, and the reverse primer was designed at the exon 55. Specific bands appeared only when a large deletion was induced from exon 45 to exon 55. To check the size of PCR amplicons, we run agarose gel electrophoresis or TapeStation analysis with High Sensitivity D5000 ScreenTape (Agilent) according to the manufacturer's protocol.

For TA cloning, the ladder bands were cut from the gel and purified by Wizard® SV Gel and PCR Clean-Up System (Promega). Then, obtained DNA fragments were TA-cloned into pGEM-T Easy Vector (Promega). After transformation into DH5α, blue-white selection and miniprep by Wizard® SV Minipreps DNA Purification Systems (Promega), inserted sequences in those plasmids were Sanger sequenced. All PCR primers are shown in Supplemental Table 2.

Skeletal muscle differentiation by doxycycline-inducible MYOD1 expression

Skeletal muscle differentiation of iPSCs was performed as described previously (Shoji *et al*, 2015; Tanaka *et al*, 2013; Uchimura *et al*, 2017). Briefly, the doxycycline-inducible human *MYOD1 piggyBac* vector (containing neomycin or puromycin resistance cassette) was integrated into the genome by electroporation together with transposase expression vector pHL-EF1a-hcPBase-A. Then, stable cells were selected with neomycin (FF12020) or puromycin (CiRA00458, CiRA00646) for 7 days. Subsequently, 1×10^5 cells/well were seeded into a 6-well plate coated with Matrigel in AK02N StemFit media with 10 μM Y-27632 (TOCRIS, MN, USA). The next day, the media was changed to Primate ES Cell Media (ReproCELL Inc., Kanagawa, Japan). On day 3, the media was changed to Primate ES Cell Media containing 2 μM doxycycline to induce *MYOD1* expression. On day 4, media was changed to skeletal muscle differentiation media composed of alpha Minimal Essential Medium (α-MEM; Nacalai Tesque) with 5% KSR (Thermo Fisher SCIENTIFIC), 100 μM 2-Mercaptoethanol and 2 μM doxycycline. After this, medium change was performed every day with the skeletal muscle differentiation media until Day 7.

Detection of proteins by Wes western blotting system

Skeletal muscle cells differentiated from iPSCs were lysed with RIPA buffer (Thermo Fisher Scientific) containing cOmplete Protease Inhibitor Cocktail (Roche). The protein concentration was quantified by the Pierce BCA Protein Assay Kit (Thermo Fisher Scientific). One microgram of samples was loaded on a Wes Simple Western system (ProteinSimple) with a 66-440 kDa Separation Module (ProteinSimple SM-W006) and the Anti-Mouse Detection Module (ProteinSimple DM-002). For detecting Dystrophin, a mouse anti-dystrophin (Rod domain) monoclonal antibody (DYS1; 1:100, Leica) and anti-mouse IgG HRP-linked antibody (ProteinSimple, 042-205) were used. For MHC

detection, the primary antibody was anti-myosin heavy chain (MAB4470, Mouse monoclonal, 1:400, R&D systems, Inc.) and the second antibody was anti-mouse IgG HRP-linked antibody (ProteinSimple, 042-205).

Assessment of exon skipping by RT-PCR

Total RNA was extracted from skeletal muscle cells differentiated from iPSCs using a NucleoSpin RNA Kit (Mecherey-Nagel GmbH & Co. KG), and cDNA was synthesized using ReverTra Ace qPCR RT Master Mix (Toyobo). Then, the exon 45-55 region was amplified by PrimeSTAR GXL DNA Polymerase (TaKaRa Bio). Sequences of primer pairs were listed in Supplemental Table 2. Multi-exon skipping was assessed by Agilent 4200 Tape Station (Agilent Technologies). Obtained PCR products were also Sangar sequenced to check the Ex44-56 junction.

Immunocytochemical staining

Differentiated cells were fixed with PBS containing 2% paraformaldehyde for 10 min at room temperature. After washing with PBS for two rounds, the cells were blocked with Blocking One PBS solution (Nacalai Tesque) for 45 minutes. Following the blocking, the cells were incubated with the primary antibodies diluted with 10% Blocking One in PBS-T (0.2% Triton X100 in PBS) overnight at 4ºC. Then, the cells were washed with PBS-T for two rounds and incubated with the secondary antibodies for one hour at room temperature. For the primary antibodies, we used anti-dystrophin (ab15277, Rabbit polyclonal, 1:100, Abcam) and anti-myosin heavy chain (MAB4470, Mouse monoclonal, 1:400, R&D systems, Inc.). For the secondary antibodies, goat anti-rabbit IgG, Alexa Fluor 546 (A-11010, Invitrogen), and goat anti-mouse IgG, Alexa Fluor 488 (Invitrogen, A-11001), were used.

Whole genome sequencing and off-target analysis

To perform whole genome sequencing, DMD-iPSC line FF12020 with two different passage numbers (passage 32 and 50) and MES-induced subclones from each parental iPSC subclones (#7-1 and #4-3) were prepared. Genomic DNA was extracted from iPSCs by MonoFas cultured cell genome DNA extraction kit VI (ANIMOS). After preparing the DNA library using KAPA Hyper Prep Kit PCR-Free Kit and IDT for Illumina-TruSeq DNA UD Indexes, genomic sequence analysis was performed by NovaSeq 6000. The obtained FASTQ files from each sample were mapped to the human genome (hg19) by BWA-MEM (0.7.15), then duplicated reads were removed by novosort (1.03.0.9). Then, SNV/Indel call was performed using Genomon (1.0.1) and Genomon2 (2.3.0), CNV call was performed using Delly (0.7.3) and VarScan (2.4.2), with the original iPSC sample with the lowest passage numbers (P32) as a reference.

To search for potential Cas3-crRNA binding sites, we used GGGenome (https://gggenome.dbcls.jp/) with up to 8 bp mismatch allowance (25% of the total length of crRNA). Any mismatches at positions 6, 12, 18, 24, and 30 were accepted because these bases are not involved in target recognition. Then, the distances between the CNV (or SNV/indel) and the potential crRNA binding site were calculated for crRNA (DMD exon 45) and crRNA (DMD exon 55), respectively. The shortest distance was collected.

Supplementary References

Hong H, Takahashi K, Ichisaka T, Aoi T, Kanagawa O, Nakagawa M, Okita K, Yamanaka S (2009) Suppression of induced pluripotent stem cell generation by the p53-p21 pathway. *Nature* 460: 1132-1135

Morisaka H, Yoshimi K, Okuzaki Y, Gee P, Kunihiro Y, Sonpho E, Xu H, Sasakawa N, Naito Y, Nakada S *et al* (2019) CRISPR-Cas3 induces broad and unidirectional genome editing in human cells. *Nat Commun* 10: 5302

Okita K, Matsumura Y, Sato Y, Okada A, Morizane A, Okamoto S, Hong H, Nakagawa M, Tanabe K, Tezuka K *et al* (2011) A more efficient method to generate integration-free human iPS cells. *Nat Methods* 8: 409-412

Shoji E, Sakurai H, Nishino T, Nakahata T, Heike T, Awaya T, Fujii N, Manabe Y, Matsuo M, Sehara-Fujisawa A (2015) Early pathogenesis of Duchenne muscular dystrophy modelled in patient-derived human induced pluripotent stem cells. *Sci Rep* 5: 12831

Tanaka A, Woltjen K, Miyake K, Hotta A, Ikeya M, Yamamoto T, Nishino T, Shoji E, Sehara-Fujisawa A, Manabe Y *et al* (2013) Efficient and reproducible myogenic differentiation from human iPS cells: prospects for modeling Miyoshi Myopathy in vitro. *PLoS One* 8: e61540

Uchimura T, Asano T, Nakata T, Hotta A, Sakurai H (2021) A muscle fatigue-like contractile decline was recapitulated using skeletal myotubes from Duchenne muscular dystrophy patient-derived iPSCs. *Cell Rep Med* 2: 100298

Uchimura T, Otomo J, Sato M, Sakurai H (2017) A human iPS cell myogenic differentiation system permitting high-throughput drug screening. *Stem Cell Res* 25: 98-106
