# Supplementary material for: Dual CRISPR-Cas3 system for inducing multi-exon skipping in DMD patient-derived iPSCs
Source: Stem Cell Reports. 2023 Aug 24;18(9):1753–65. doi: 10.1016/j.stemcr.2023.07.007 (PMC10545483; doi:10.1016/j.stemcr.2023.07.007)
Supplement: Document S2. Article plus supplemental information [file mmc2.pdf]

Stem Cell Reports
Report
OPEN ACCESS

Dual CRISPR-Cas3 system for inducing multi-exon skipping in DMD patient-derived iPSCs

Yuto Kita,[1] Yuya Okuzaki,[2] Youichi Naoe,[1] Joseph Lee,[1] Uikyu Bang,[1] Natsumi Okawa,[1] Akane Ichiki,[1] Tatsuya Jonouchi,[1] Hidetoshi Sakurai,[1] Yusuke Kojima,[1] and Akitsu Hotta[1,3,*]
[1]Center for iPS Cell Research and Application, Kyoto University, 53 Shogoin-Kawahara-cho, Sakyo-ku, Kyoto 606-8507, Japan
[2]Nagoya University Graduate School of Bioagricultural Sciences, Avian Bioscience Research Center, Furo-cho, Chikusa-ku, Nagoya, Aishi 464-8601, Japan
[3]Takeda-CiRA Joint Program (T-CiRA), Fujisawa, Kanagawa 251-8555, Japan
*Correspondence: akitsu.hotta@cira.kyoto-u.ac.jp
https://doi.org/10.1016/j.stemcr.2023.07.007

SUMMARY

To restore dystrophin protein in various mutation patterns of Duchenne muscular dystrophy (DMD), the multi-exon skipping (MES) approach has been investigated. However, only limited techniques are available to induce a large deletion to cover the target exons spread over several hundred kilobases. Here, we utilized the CRISPR-Cas3 system for MES induction and showed that dual crRNAs could induce a large deletion at the dystrophin exon 45–55 region (∼340 kb), which can be applied to various types of DMD patients. We developed a two-color SSA-based reporter system for Cas3 to enrich the genome-edited cell population and demonstrated that MES induction restored dystrophin protein in DMD-iPSCs with three distinct mutations. Whole-genome sequencing and distance analysis detected no significant off-target deletion near the putative crRNA binding sites. Altogether, dual CRISPR-Cas3 is a promising tool to induce a gigantic genomic deletion and restore dystrophin protein via MES induction.

INTRODUCTION

Duchenne muscular dystrophy (DMD) is a severe muscle degeneration disorder caused by genomic mutations that result in a frameshift in the *dystrophin* gene (Koenig et al., 1988). DMD is known to be the most frequent and severe type of muscular dystrophy (Mercuri et al., 2019); however, there is no curative treatment so far. The *dystrophin* gene is one of the largest protein-coding genes (∼2 Mb) in the human genome, in which the skeletal muscle isoform of Dp427m consists of 79 exons translated to a 427-kDa dystrophin protein (Muntoni et al., 2003). The dystrophin protein stabilizes muscle cells or myofibers by binding to the actin cytoskeleton on the N terminus and the muscle cell membrane on the C terminus (Fairclough et al., 2013). So far, more than 7,000 kinds of mutations in the *dystrophin* gene have been identified in DMD patients (Bladen et al., 2015), and such mutations disrupt the open reading frame and lead to the loss of functional dystrophin protein.

To restore dystrophin protein, exon skipping is a promising approach to correct the open reading frame. There are already a couple of antisense oligonucleotide (ASO)-based exon skipping drugs clinically approved for DMD treatment (Roshmi and Yokota, 2021). However, the ASO strategy skips exons at the RNA level and doesn't correct causal genomic mutations; hence, DMD patients require repeated weekly injections throughout their life.

Recently, the induction of exon skipping at the genomic DNA level has been expected to be an emerging approach that can have a longer-lasting effect than ASO. Especially the development of the CRISPR-Cas9 system has revolutionized the field of genome editing (Doudna, 2020). Many groups have utilized the CRISPR-Cas9 system to induce genomic mono-exon skipping *in vitro* (Kagita et al., 2021; Li et al., 2015; Ousterout et al., 2015) and *in vivo* (Amoasii et al., 2017; El Refaey et al., 2017; Gee et al., 2020; Kenjo et al., 2021; Koo et al., 2018; Long et al., 2016; Min et al., 2019; Nelson et al., 2016; Tabebordbar et al., 2016; Xu et al., 2019; Zhang et al., 2020).

However, due to the significant variations of the mutation patterns in the *dystrophin* gene, the mono-exon skipping approach can only be used for a limited number of DMD patients. For example, the most common mono-exon skipping of exons 51, 53, and 45 can be applied to 13%, 8%, and 8% of DMD patients, respectively (Saifullah et al., 2022). To broaden the patient applicability, the multi-exon skipping (MES) approach by targeting the exons from 45 to 55 has been proposed (Béroud et al., 2007). By targeting the mutation hotspots in the *dystrophin*, MES from exon 45 to 55 was estimated to restore the *dystrophin* open reading frame in more than 60% of DMD patients (Echigoya et al., 2018). Although this approach produces a shorter dystrophin protein, multiple clinical studies have reported that an in-frame deletion of exon 45–55 results in a very mild phenotype or sometimes asymptomatic even in their 60s (Ferreiro et al., 2009; Nakamura et al., 2008). To induce MES at the RNA level, a cocktail of 11 ASOs has been demonstrated to restore dystrophin protein, therefore improving muscle function in a mouse model (Aoki et al., 2012). To develop a long-lasting drug for inducing MES at the genomic DNA level, it is

This is an open access article under the CC BY license (http://creativecommons.org/licenses/by/4.0/).

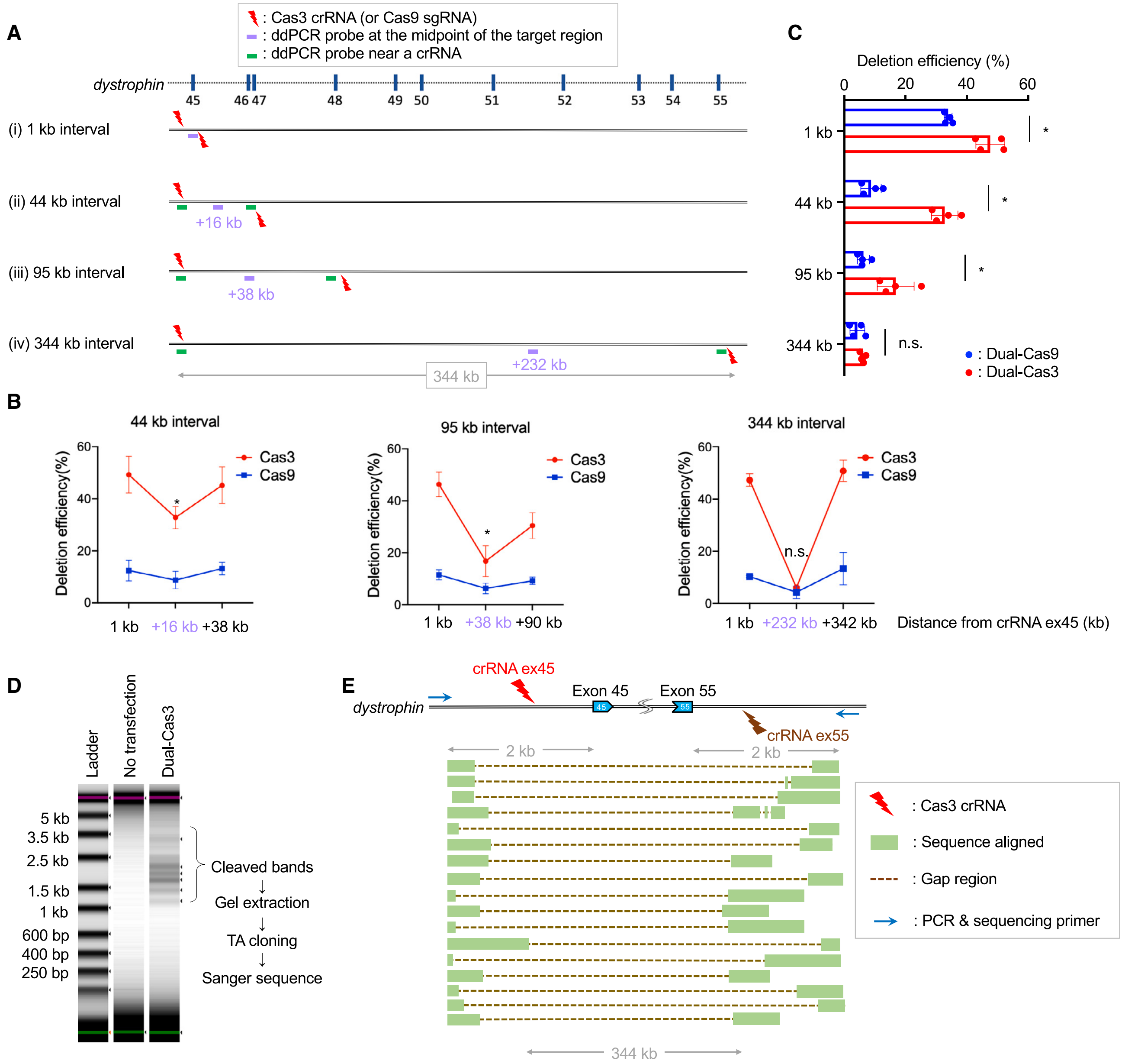

Figure 1. Induction of a large deletion by the dual-Cas3 system

(A) Schematic overview of the experiments to compare the deletion efficiency between Cas3 and Cas9 at various distances. Multiple pairs of Cas3-crRNAs or Cas9-sgRNAs were designed upstream and downstream of the target region, as indicated by red thunder marks. The orientation of the edge of the thunder mark indicates the PAM sequence, and Cas3 induces deletion in this direction. Each target region is (i) 1 kb, (ii) 44 kb, (iii) 95 kb, and (iv) 344 kb distal between two crRNA (or sgRNA) targeting sites at the *dystrophin* gene locus. To quantify deletion efficiencies, ddPCR probes were designed at the approximate midpoint of the target region, shown as a purple horizontal bar, and near the crRNA binding site, shown as a green horizontal bar.

(B) The deletion efficiency is calculated as DNA copy number loss by ddPCR at the indicated position in HEK293T cells. The activity of each crRNA was examined by the ddPCR probes indicated in (A) as green bars. Copy number loss at the approximate midpoint of the target region (+16 kb, +38 kb, and +232 kb probes for the 44-kb, 95-kb, and 344-kb intervals, respectively) is considered as the induction of large deletion. Data were represented as means ± SD from independent experiments (n = 4). Two-tailed unpaired t test was used to calculate p values. *$p < 0.05$.

(C) The summary of the deletion induction efficiency at the midpoint from (B). Data were represented as means ± SD from independent experiments (n = 4). A two-tailed unpaired t test was used to calculate p values. *$p < 0.05$.

(legend continued on next page)

required to remove the exon 45–55 region, which spans at least 344 kb in the human chromosome (Young et al., 2016). However, the induction method to induce several hundred kilobases genomic deletions has not been thoroughly investigated.

Recently, we and others have shown that class 1 type I-E CRISPR-Cas3 can be utilized as a genome editing tool in mammalian cells (Cameron et al., 2019; Dolan et al., 2019; Morisaka et al., 2019). Unlike the class 2 type II CRISPR-Cas9 system, the type I-E CRISPR-Cas3 system consists of the multi-subunit complex, Cascade/crRNA, to bind the target sequence, and Cas3 enzyme with the DNA helicase and nickase activity to shred the target DNA processively toward PAM proximal side (Loeff et al., 2018). Thus, CRISPR-Cas3 induces a long range of deletions from a few hundred base pairs to a hundred kilobase pairs from the crRNA target site in a unidirectional manner. By utilizing this property, we previously demonstrated that a single Cas3-crRNA induced genomic mono-exon skipping in the *dystrophin* gene (Morisaka et al., 2019); however, targeted genomic deletion of several hundred kilobases and the feasibility of MES were unexplored.

In this study, we sought to induce genomic MES by multiplexing Cas3-crRNAs. We found that dual-Cas3 can cause a large deletion of up to 340 kb. We investigated the optimal crRNA combinations for MES induction in DMD-iPSCs and developed a reporter system to enrich genome-edited cells. Finally, to examine the risk of off-target mutagenesis, we performed whole-genome sequencing of the genome-edited subclones and investigated the deletions associated with putative crRNA binding sites. These results suggest the potential of the dual CRISPR-Cas3 system to induce large genomic deletions for MES induction in DMD patients with a wide variety of mutation patterns.

RESULTS

Dual CRISPR-Cas3 to induce several hundred kilobases of deletions

Since it was rare to observe a deletion of more than a hundred kilobases using a single Cas3-crRNA, we sought to use a pair of crRNAs inwardly sandwiching the target genomic region. To assess how this dual-Cas3 approach could feasibly induce large deletions, we designed one crRNA fixed near the exon 45 of the *dystrophin* gene and its counterpart crRNAs at 1 kb, 44 kb, 95 kb, and 344 kb positions from the fixed crRNA (Figures 1A and S1A and Table S1). As a comparison, we also designed Cas9-sgRNAs in similar positions. After transfection of plasmid DNA expression vectors of Cas3/Cascade/crRNAs or Cas9/sgRNAs, we measured genomic copy numbers at the approximately halfway points of the crRNA pairs using droplet digital PCR (ddPCR) to quantify deletion efficiency (Figure 1A, purple bars). The deletion efficiencies at proximal positions to designed crRNA (within 6 kb) were also monitored to validate the activities of crRNAs (Figure 1A, green bars). In HEK293T cells, the losses of DNA copy number at the exon 45 (+1 kb probe) and exon 55 (+342 kb probe) were more evident with dual-Cas3 than dual-Cas9 (Figure 1B). In addition, the copy number losses at the half-points were significantly higher with 44-kb and 95-kb intervals. The apparent copy number loss with the 344-kb interval was also clearly detectable using dual-Cas3, though there was no statistical difference between Cas3 and Cas9 (Figure 1C). In addition to the inward dual-Cas3 system, we also tested parallel and outward orientations for the dual-Cas3 (Figure S1B). Unexpectedly, we found that the parallel and outward dual-Cas3 could also induce large deletions at similar efficiency to the inward manner. To avoid unnecessary deletions outside of the crRNA target sites, we decided to use the inward dual-crRNA method in the following experiments.

Next, we examined the deletion patterns induced by the inward dual-Cas3 with the 344-kb interval in HEK293T cells. For this, the target genomic region was amplified using the PCR primers upstream of exon 45 and downstream of exon 55. Due to the long distance between the two primers, amplification occurs only when large deletions are induced. Indeed, PCR results showed multiple bands in the genome-edited cells but not in the untreated cells (Figure 1D). These bands were gel-extracted and sequenced after TA cloning. As a result, we observed various patterns of deletions around 344 kb in size (Figure 1E). Interestingly, in many cases, the deletion extended beyond the crRNA target sites; therefore, both crRNA binding sites were lost. This contrasts with the single Cas3-crRNA, as crRNA binding sites are usually maintained (Morisaka et al., 2019).

In addition to HEK293T cells, we also performed genome editing with the dual-Cas3 in the DMD patient-derived iPSC line FF12020, which lacks exons 46 and 47 (Δexon 46, 47). PCR analysis of the target region similarly showed ladder band patterns (Figure S1C), and Sanger sequencing of the multiple PCR products following TA cloning showed

(D) PCR analysis to investigate the deletion patterns in HEK293T. After transfection of a pair of Cas3-crRNA, the *dystrophin* exon 45–55 region was PCR amplified using indicated primers and visualized by TapeStation with high-sensitivity D5000 DNA tape.
(E) Sanger sequencing results of PCR amplicons from (D). Ladder PCR amplicons were subjected to TA cloning, and each clone was analyzed by Sanger sequencing (17 clones). Matching regions to the *dystrophin* gene are shown in green boxed lines, and gap regions are shown as broken lines.

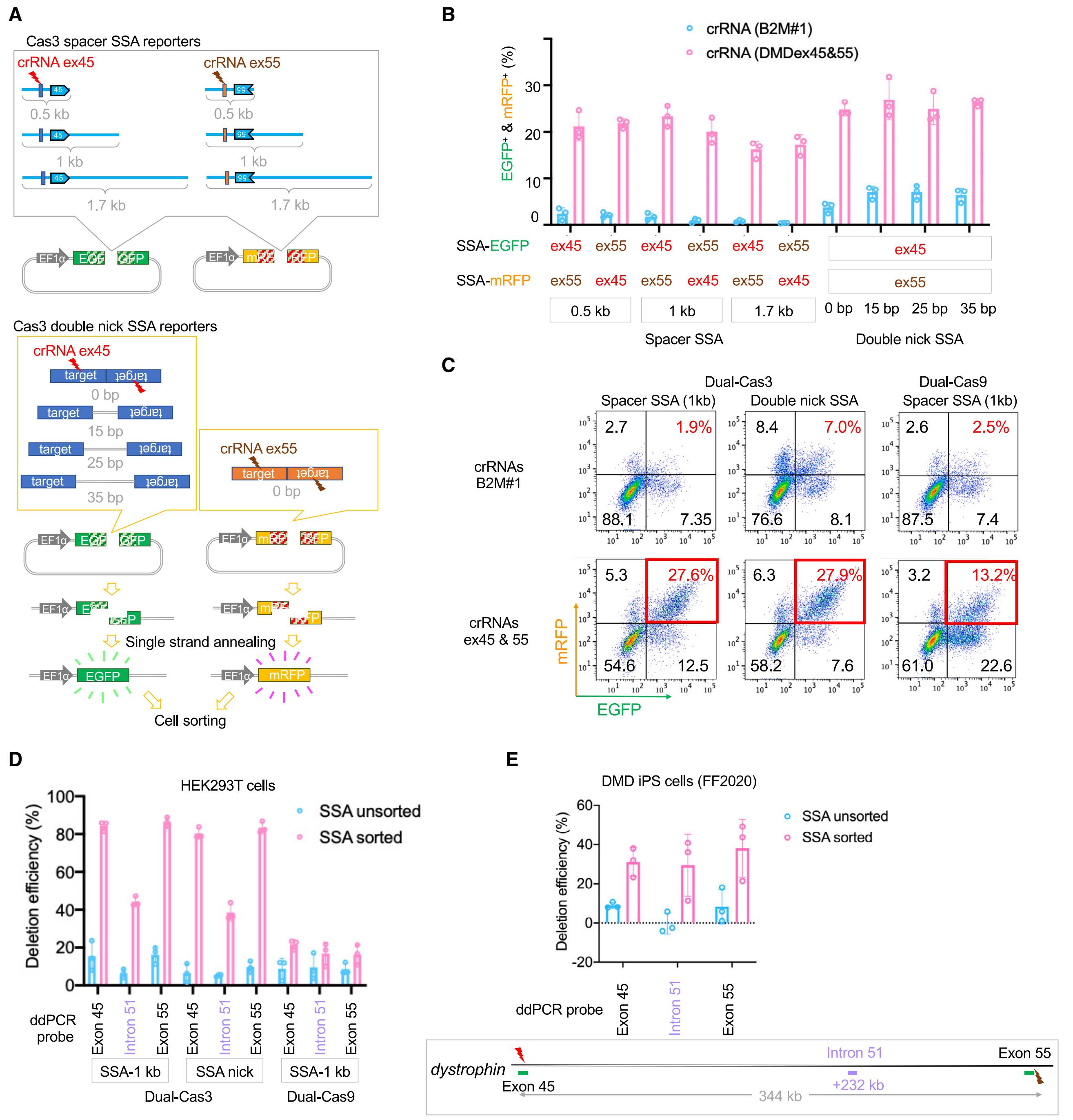

Figure 2. Enrichment of Cas3-active cells by single-strand annealing (SSA) reporter

(A) Schematic of various SSA reporter vectors for monitoring dual-Cas3's activity. Structures of SSA vectors with long inserts with various insert lengths (0.5, 1.0, and 1.7 kb) or double-nick inserts containing a spacer (0, 15, 25, and 35 bp) are constructed. When Cas3 or Cas9 induces a DNA break at the target sequence (blue boxed bar), the single-strand annealing (SSA) DNA repair pathway restores the functional cDNA sequence of EGFP or mRFP through the two homology sequences (indicated by the slashed box).

(B) Evaluation of the SSA vectors by the dual-Cas3 system. Two-color SSA vectors (EGFP or mRFP) containing a crRNA target sequence (DMD ex45 or ex55) were transfected into HEK293T cells together with the expression vectors of Cas3, Cascade, and two crRNAs that target DMD ex45 and ex55, as "targeting crRNA." As negative control to evaluate the background fluorescence activity of the SSA reporters, we used a

(legend continued on next page)

large deletions spanning the exons 45 and 55 at the *dystrophin* gene (Figure S1D). Altogether, we demonstrated that the inward dual-Cas3 strategy successfully induced up to 344-kb large deletions not only in HEK293T cells but also in DMD patient-derived iPSCs.

Enrichment of the dual-Cas3-edited cells using single-strand annealing reporter

Although we successfully induced 344-kb large deletions by Cas3, the efficiency in generating such a gigantic deletion was not high enough for subcloning in DMD patient-derived iPSCs (Figure S3G). In previous studies, genome-edited cell populations by Cas9 could be enriched using single-strand annealing (SSA) reporter vector followed by cell sorting (Mashiko et al., 2013). Therefore, we sought to enrich the genome-edited cell population using a similar system adapted for our dual-Cas3.

In the SSA reporter system, a spacer sequence containing a crRNA target site was inserted into a split fluorescent protein gene with homology sequences for SSA repair after DNA damage. First, we considered the optimal sequence length of the target insert for Cas3 because Cas3 induces large deletions rather than small deletions. The short target sequences typically used for Cas9 (e.g., just the crRNA recognition site) might not be suitable for Cas3. Therefore, we inserted relatively long spacer sequences (0.5–1.7 kb) containing the crRNA recognition site and extended the target sequence in the direction of Cas3 deletion (Figure 2A). In addition to the spacer SSA reporter, we also constructed double-nick-based SSA reporters, considering that Cas3 is a nickase. We hypothesized that a palindromic crRNA recognition site with a spacer might induce DNA double-strand break by induction of double nicking at each DNA strand (Figure 2A). Lastly, to monitor the activity of both sides of the crRNAs in the dual-Cas3 system, we constructed both EGFP (enhanced green fluorescence protein) and mRFP (monomeric red fluorescence protein) versions of the SSA vectors. We used these vectors and two Cas3-crRNAs to perform two-color SSA reporter assays in HEK293T cells. Our results showed significantly higher EGFP and mRFP signals only when using targeting crRNAs (DMD ex45 and ex55) but not with a non-targeting crRNA (B2M #1) (Figure 2B). Among the spacer SSA vectors, the background signals with the non-targeting crRNA were decreased when the insert spacer was elongated from 0.5 to 1.7 kb. Although the double-positive signals were detected with the three spacer SSA reporters, a slight decrease was observed with the 1.7 kb spacer. All double-nick SSA reporters showed higher on-target and background signals than long target SSA vectors, regardless of a spacer length. Therefore, we chose the target insert with 1 kb long for the spacer SSA reporters and no spacer sequence for double-nick SSA reporters because of their relatively high signal-to-noise ratio.

Next, we tested whether the genome-edited population can be enriched by cell sorting utilizing the two-color SSA reporter vectors in HEK293T cells. After transfection of the Cas3/Cascade/crRNA expression vectors and the two-color SSA vectors, EGFP and mRFP double-positive population was sorted by a cell sorter (Figure 2C). Genome editing efficiency of ~340 kb deletion in HEK293T cells was estimated by ddPCR measuring copy number at the midpoint of 340 kb (intron 51, +232 kb). Compared with the unsorted population, the sorted population showed significantly higher deletion efficiency, up to 40% (Figure 2D). Similar enrichment was also observed in DMD patient-derived iPSC line FF12020 using 1-kb-long target SSA reporters, and around 30% of copy number loss was seen at the midpoint (Figure 2E). Taken together, we demonstrated that the two-color SSA reporter system can enrich the genome-edited population by dual-Cas3 to facilitate the isolation of cells with a large deletion.

MES induction with multiplexed Cas3-crRNAs

In addition to the dual-Cas3 approach (2× crRNAs), we also tested whether using more than two crRNAs enhances the efficiency of ~340-kb large deletion. For this, we designed four crRNAs (4× crRNAs) with about 110-kb intervals spanning the 340-kb region and 11 crRNAs (11× crRNAs) targeting all 11 exons between 45 and 55 exons (Figure S2A). In

crRNA that targets the B2M gene, as "non-targeting crRNA." 3 days after transfection, the percentages of EGFP and mRFP double-positive cells were analyzed by flow cytometry. Data were represented as means ± SD from independent experiments (n = 3).

(C) Representative FACS plots and gating for cell sorting. After transfection of the SSA vectors (1-kb-long spacer or double nick with 0-bp spacer) as described in (B), cell sorting of the EGFP and mRFP double-positive population (red rectangle area) was performed. As a control, we used Cas9 and two sgRNA-expression vectors targeting exons 45 and 55.

(D) The deletion efficiency in the sorted cells with the two-color SSA vectors. Genomic DNAs of sorted HEK293T cells were subjected to ddPCR analysis to check the deletion efficiency with the ddPCR probes at exons 45 (+1 kb), 55 (+342 kb), and the midpoint (+232 kb). Data were represented as means ± SD from independent experiments (n = 3).

(E) The deletion efficiency in DMD patient-derived iPSC FF12020 line sorted by the two-color SSA reporter system. Two SSA vectors (EGFP for exon 45 target, mRFP for exon 55 target) with 1-kb spacer were transfected into FF12020 iPSCs, and cell sorting was performed 3 days post-transfection. The sorted cells were similarly analyzed by ddPCR as described in (D). Data were represented as means ± SD from independent experiments (n = 3).

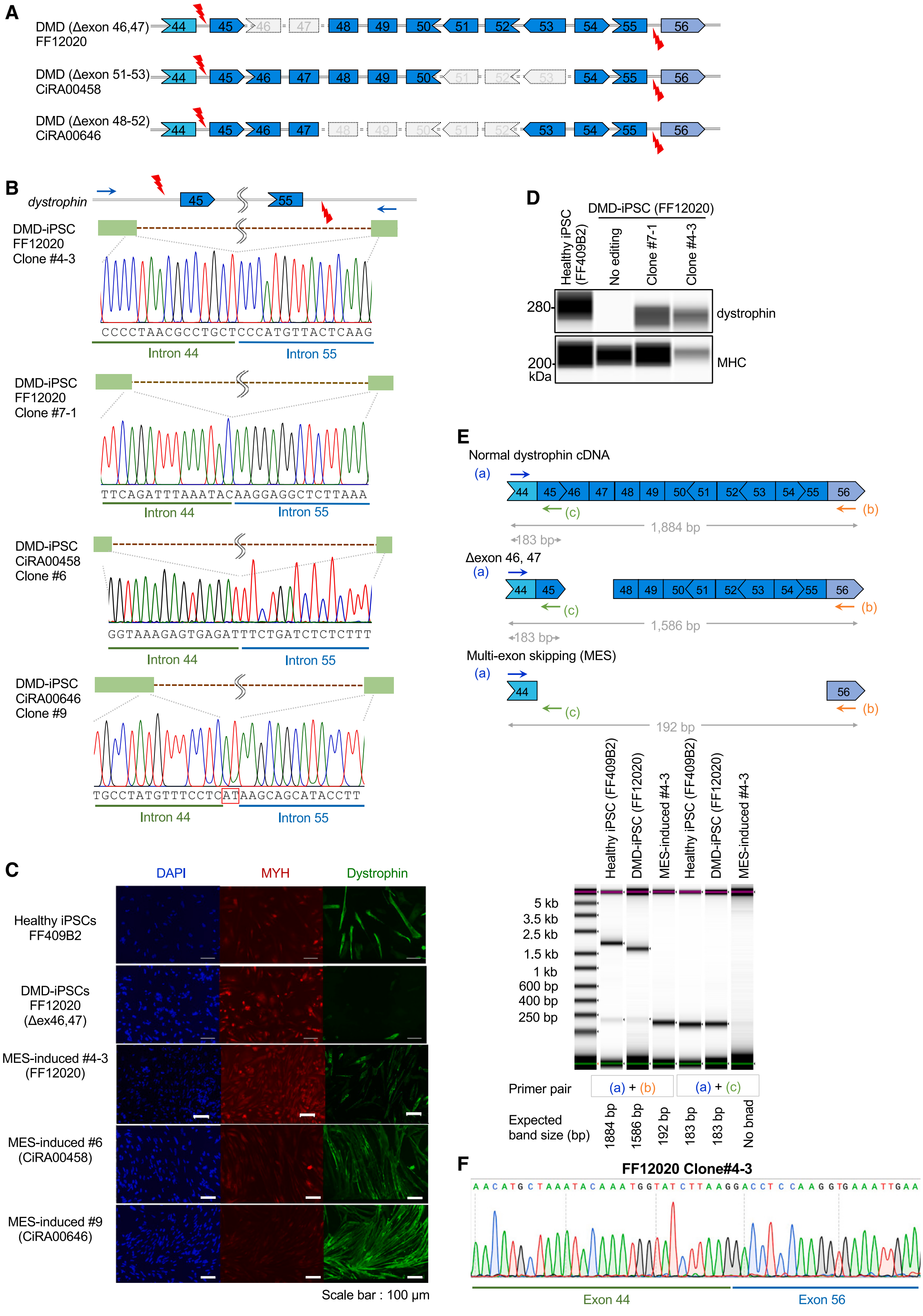

(legend on next page)

HEK293T cells, 4× and 11× crRNAs showed greater deletion efficiencies at the middle five probes when assessed by ddPCR (Figure S2B). However, when similar genome editing using 2×, 4×, and 11× crRNAs were performed in DMD-iPSC line FF12020, the recovery rate of dystrophin protein after muscle differentiation in 4× and 11× crRNA samples did not increase compared with the 2× crRNA sample by western blotting (Figures S2C and S2D). In addition, we performed immunostaining to detect dystrophin protein recovery. However, the trend of the dystrophin recovery rate was not consistent among 2×, 4×, and 11× crRNA samples (Figures S2E and S2F). We speculate that a variety of deletion patterns caused by multiple crRNAs would lead to suboptimal in-frame deletions; therefore dystrophin protein recovery did not match with the ddPCR results. Because of these results and the complexity of using multiple crRNAs, we decided to use the dual-Cas3 approach for downstream experiments.

MES induction in various DMD patient-derived iPSCs

The advantage of the MES strategy is its broad applicability to various DMD mutation types located within exon 45–55. To demonstrate this, in addition to the FF12020 iPSC line (Δexon 46, 47), we established two additional iPSC lines, CiRA00458 (Δexon 51–53) and CiRA00646 (Δexon 48–52), from DMD patients (Figures S3A and S3B). With a total of three iPSC lines, we performed dual-Cas3-based genome editing (Figure 3A), followed by enrichment with the two-color SSA reporters and single-cell sorting. After subcloning, PCR genotyping (Figures S3C–S3F), and Sanger sequencing (Figures 3B and S3E), we successfully obtained subclones with exon 45–55 skipping from all the parental iPSC lines (Figure S3G and S3H: 21 clones out of 98 clones screened for FF12020, one clone out of three clones for CiRA00458, and one clone out of six clones for CiRA00646). Next, we confirmed the dystrophin protein recovery of the subclones (FF12020 #4–3, #7–1, CiRA00458 #6, CiRA00646 #9) by skeletal muscle differentiation. Immunocytochemical staining results showed the apparent restoration of dystrophin protein by the dual-Cas3-mediated deletion in the subclones (Figure 3C). Furthermore, we also confirmed the dystrophin protein recovery by the western blotting system using FF12020 #4–3 and #7–1 (Figure 3D). In this analysis, a band shift of the dystrophin protein due to the truncation of the exon 45–55 region was observed in these subclone samples. RT-PCR also confirmed the induction of MES at the mRNA level in the FF12020 #4-3 clone (Figure 3E). Sanger sequencing of the cDNA from #4-3 clone clearly showed the in-frame junction of exons 44 and 56 (Figure 3F).

Overall, these results demonstrated that the dual-Cas3 system was able to induce the genomic MES in DMD-iPSCs with various mutations and restored dystrophin protein, emphasizing the broad applicability of our dual-Cas3 strategy.

Off-target analysis of the dual-Cas3 by whole-genome sequencing

Finally, we assessed the potential off-target risks caused by the dual-Cas3 in the genomic MES-induced subclones. To distinguish off-target mutations from spontaneous mutations induced by cell culturing and subcloning, we prepared two sets of samples (Figure 4A). MES-induced subclone #7–1 was established from unedited parental DMD patient-derived iPSC line FF12020 at passage 32 (NC1), and another MES-induced subclone #4–3 was established

Figure 3. The dual-Cas3 genomic MES induction in multiple patient-derived iPSCs

(A) Schematic illustrations of MES induction in three DMD patients. Three DMD-iPSC clones—FF12020 iPSC clone (Δexon 46, 47), CiRA00458 clone (Δexon 51–53), and CiRA00646 clone (Δexon 48–52)—were subject to dual-Cas3 genome editing using two crRNAs indicated by thunder marks. The shapes of exons illustrate codon frames.

(B) Deletion patterns of genomic MES-induced DMD-iPSC subclones. Genomic MES-induced iPSC subclones (FF12020 #4–3, #7–1, CiRA00458 #6, and CiRA00646 #9) were established by the dual-Cas3 genome editing followed by the two-color SSA-based single-cell sorting. Genomic DNA samples were PCR amplified using indicated primers (arrows), and deletion patterns were analyzed by Sanger sequencing. A red square indicates the insertion sequence.

(C) Confirmation of dystrophin protein recovery by immunocytochemical staining. Genomic MES-induced subclones in (B) were differentiated into skeletal muscle cells by MYOD1 expression, and immunocytochemical staining was performed for MYH and dystrophin. Nuclear staining was performed with DAPI. Healthy donor-derived iPSCs (FF409B2) and unedited FF12020 iPSC are shown as controls. Scale bars indicate 100 μm.

(D) Confirmation of dystrophin protein recovery by Wes automated western blotting system. Healthy iPSC (FF409B2), DMD-iPSC (FF12020: Δexon 46, 47), and genomic MES-induced clones #7–1 and #4–3 were differentiated into skeletal muscle cells, and dystrophin and MHC proteins were detected by the Wes system.

(E) Confirmation of multi-exon skipping at mRNA level by RT-PCR. Primers to distinguish MES-induced mRNA from uninduced ones are indicated (top). RNA samples from healthy donor iPSC (FF409B2), DMD-iPSC (FF12020: Δexon 46, 47), and MES-induced FF12020 subclone #4–3 were reverse-transcribed and PCR amplified using indicated primer pairs. TapeStation result is shown, and expected band sizes are shown below.

(F) Direct sequencing of cDNA from genomic MES-induced clone #4–3. PCR product from #4–3 in (D) amplified by primer (a)+(b) was Sanger sequenced. In-frame junction of exon 44 and exon 56 is shown.

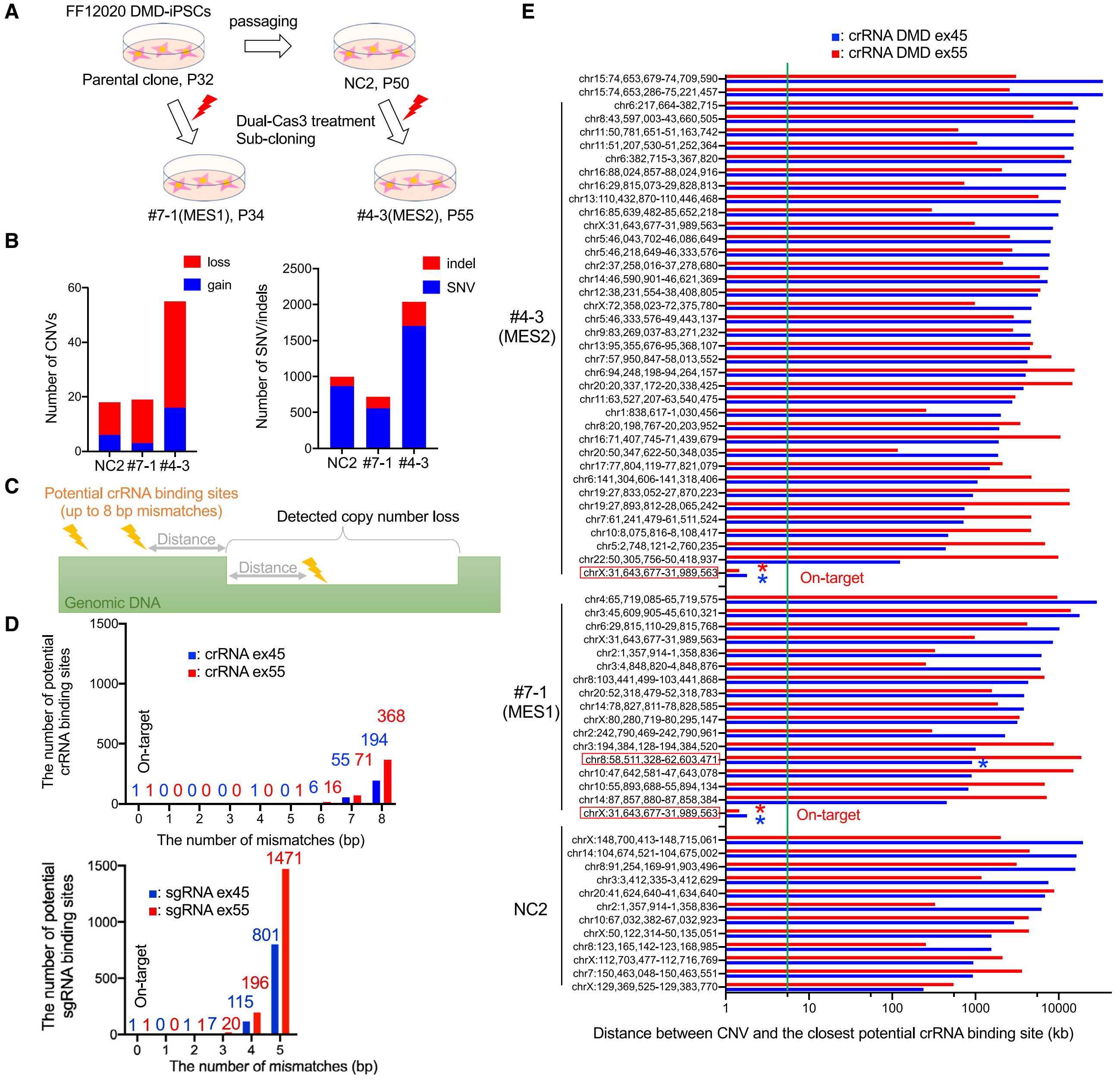

Figure 4. Whole-genome sequencing for detecting off-target cleavages by dual-Cas3

(A) Schematic diagram of the clones assessed by whole-genome sequencing. Two genomic MES-induced FF12020 subclones (#7–1 and #4–3) were established from their parental FF12020 cells at different passage numbers (NC1 at p32, NC2 at p50). These four samples were subject to whole-genome sequencing.

(B) Summary of detected CNVs, indels, and SNVs. The number of *de novo* CNVs (left) and indel/SNVs (right) were counted using NC1 data as a reference.

(C) Schematic of distance calculation between potential crRNA binding sites and the edge of detected CNVs. To estimate bona fide off-target CNVs induced by Cas3, the distance between the closest crRNA binding site and a detected CNV was calculated.

(D) The number of potential Cas3 crRNA/Cas9-sgRNA binding sites. Potential crRNA binding sites of two crRNAs targeting exon 45 and exon 55 were searched by GGGenome software with an 8-base mismatches allowance. A total of 256 potential off-target sites for ex45 and 456 sites for crRNA ex55 were found in the human genome (top). Potential sgRNA binding sites of two sgRNAs targeting exon 45 and exon 55 were searched by GGGenome with a 5-base mismatches allowance. A total of 924 and 1,688 off-target sites for ex45 and ex55 were found, respectively (bottom).

(legend continued on next page)

from the FF12020 at passage 50 (NC2). Whole-genome sequencing was performed using these four samples with an average sequencing depth of 36.9. CNVs (copy number variations), indels (insertions and deletions), and SNVs (single nucleotide variations) were detected by using the NC1 sample as a reference (Tables S3 and S4). We confirmed that the on-target 344-kb deletion induced by dual-Cas3 was clearly detected as a CNV in the #7–1 and #4-3 clones (Table S3, CNV ID: 77).

As previously reported (Martins-Taylor et al., 2011), some *de novo* CNVs were detected in the NC2 sample, in which the only difference is the passage number of 18 (Figure 4B). Next, to distinguish Cas3-mediated CNVs from spontaneous ones, we hypothesized that Cas3-induced mutations should accompany a putative crRNA binding site nearby. For this, we evaluated the distance of each detected CNV from the nearest potential crRNA binding sites (Figure 4C). In our previous experiences, 99% of the Cas3-mediated deletion events start within 5 kb from a crRNA binding site (Morisaka et al., 2019). Therefore, if detected CNVs are outside of this region, those are most likely not due to the Cas3-crRNA activity.

Firstly, we extracted the list of potential crRNA binding sites for both crRNAs targeting *dystrophin* exon 45 and 55 using GGGenome software, allowing up to 8-base mismatches out of 27-base target sequence recognition, considering that every six base positions are not involved in target sequence recognition. As a result, we identified 257 sites for crRNA ex45 and 457 sites for crRNA ex55 in total (Figure 4D). Of note, the number of potential off-target sites is much less than that of Cas9-sgRNA as many more potential off-target sites were already predicted for Cas9-sgRNAs with up to five mismatches (Figure 4D). This is due to the longer target recognition ability of the Cas3-crRNA (27 nt) compared with Cas9-sgRNA (20 nt). Next, we calculated the distance between a CNV and the nearest potential crRNA binding site (Figure 4E). Among the total 67 CNV losses detected (13 CNVs from NC2, 16 from #7–1, and 38 from #4–3), only the on-target CNVs have the crRNA binding site within the 5-kb window, but all other CNV losses were outside, suggesting that those CNVs were unlikely to be generated by the Cas3/crRNA. Of note, among 85 CNVs identified, one 4,058-kb CNV was found to have a potential crRNA (ex45) binding site inside of the CNV at 908 kb and 3184 kb from upstream and downstream edges, respectively (Figure S4A). This is far beyond the 5-kb window from the potential crRNA binding site. Furthermore, this potential crRNA site has 8-bp mismatches, among which three mismatches are located within the crRNA seed sequence (within 8-bp region from PAM). Considering even one mismatch in the seed sequence almost abolished the activity of Cas3 (Morisaka et al., 2019), it is unlikely the CNV is due to the off-target cleavage.

Although the Cas3 tends to induce a CNV loss (large deletion), we also evaluated the association of Cas3-crRNA with the detected indels and SNVs. Like CNVs, a greater number of SNV/indels were detected in NC2, #7–1, and #4-3 samples compared with the parental NC1. A similar distance calculation of SNV/indels from the closest potential crRNA binding sites revealed that most (99.9%) of the detected SNV/indels were located more than 5 kb away from the potential crRNA binding sites (Figure S4B). Notably, we found one SNV out of 717 SNV/indels in the #7-1 clone, and six SNV/indels out of 2,038 SNV/indels in the #4-3 clone were located within 5 kb from a potential crRNA binding site (Figure S4C). However, all the nearest crRNAs have mismatches of 7 or 8 bp, and half of the crRNAs pointed to the opposite direction of those SNV/indels. Considering that we searched for relatively large 7.14-Mb regions (714 crRNAs, ±5 kb) out of 3.117-Gb genome, we asked whether the odds of finding a few SNV/indels were within the range of chance occurrence. As a result, there was no significant difference in the frequency of SNV/indels appearance between the NC2 and #7–1 or between NC2 and #4–3 (Figure S4D).

Taken together, these whole-genome sequencing analyses demonstrated no apparent off-target cleavage caused by dual-Cas3 system, suggesting the high specificity of the dual-Cas3 system.

DISCUSSION

MES induction is one of the promising approaches for DMD treatment because of its broad applicability to various mutation patterns. In this study, we took advantage of the unique feature of CRISPR-Cas3 to induce unidirectional deletion and developed a dual Cas3 system for genomic MES induction.

First, we assessed the potential of the dual-Cas3 approach to induce large deletions and showed that dual-Cas3 is available to generate up to ∼340-kb deletion not only in HEK293T but also in iPSCs (Figures 1 and S1).

Second, to facilitate the isolation of cells edited with dual-Cas3 system, we established an SSA reporter-based

(E) Distances between the detected CNVs and the closest potential crRNA off-target sites. Blue bars indicate the distance from the potential binding sites for crRNA ex45, and red bars indicate the distance from the potential binding sites for crRNA ex55. As a threshold window, the 5-kb line (within which 99% of the Cas3 cleavage starts) is indicated as a green vertical line. The asterisk indicates the potential crRNA binding site detected within the CNV.

two-color cell enrichment technique. Using this, we successfully enriched ~340-kb-deleted cell populations up to 40% in HEK293T cells and 30% in iPSC, respectively (Figures 2D and 2E). Of note, we also tested whether the multiplexed Cas3 system (4× and 11× crRNAs) could further improve the editing efficiency or not. However, it was difficult to draw a conclusion about the efficiency of 4× and 11× crRNA methods because of the discrepancy among ddPCR, western blotting, and immunofluorescence results and no obvious improvement in the recovery efficiency (Figure S2). Therefore, we decided to focus on the dual-Cas3 system for the downstream experiments.

Third, to show the broad applicability of dual-Cas3 system, we induced genomic MES in three different DMD patient-derived iPSCs (Figure 3A). By utilizing the SSA reporters, we successfully isolated genome-edited subclones from those iPSCs, and the restoration of dystrophin protein was confirmed after skeletal muscle differentiation.

Finally, we performed whole-genome sequencing and assessed the potential association of the detected CNVs/indels/SNVs with off-target activity. We searched for the nearest potential crRNA binding site from the breakpoint of the detected CNVs, and all the detected CNVs were distal more than 5 kb (Figure 4E). Consistent with our previous study (Morisaka et al., 2019), highly specific target recognition by 27-base-long crRNA may result in the high specificity of the Cas3 system.

For inducing extremely large deletions, many techniques have been developed previously. For example, the classical approach uses the Cre-loxP system. Although two exogenous *loxP* sequences must be inserted in advance, this method allows efficient and precise large genome editing. In DMD studies, for example, ~2.4-Mb whole *dystrophin* gene was deleted in mice (Kudoh et al., 2005), and our group also successfully introduced a 342-kb deletion at the *dystrophin* gene (exon 45–55) in iPSCs by Cre-loxP recombination (Kagita et al., 2021).

After the discovery of Cas9, dual-Cas9 system has been reported to induce a large deletion in murine erythroleukemia cells (Canver et al., 2014). In fact, dual-Cas9 has been used in iPSCs for MES induction in the *dystrophin* gene, although the editing efficiency was not shown (Young et al., 2016). Alternatively, several groups reported that dual-prime editors introduced up to 10-kb deletions in HEK293T cells, although the efficiency of 10-kb deletion was still 1%–7% (Choi et al., 2022; Jiang et al., 2022). In addition, large knockin or gene substitution techniques can be potentially applied to induce a large deletion. The ssODN-mediated method was used for the gene substitution up to 58 kb by combining multiple Cas9-sgRNAs and ssODNs in rat embryos (Yoshimi et al., 2016). Furthermore, the two-step Universal Knock-in System was shown to induce more than 200-kb deletion in HCT116 cells (Ohno et al., 2022). These methods use homology-directed repair mechanisms and can potentially generate large deletions, although their availability for MES induction remains to be investigated.

Of note, there are potential limitations of the dual- or multiplexed-Cas3 systems. First, there is a variation in the deletion pattern, and the precise start and endpoints of the deletion cannot be fully controlled (Figure 1E). This could be a drawback when a large but precise deletion is required. However, in the case of DMD, the disparity of the deletion size should be acceptable because the target exons are separated by large intron sequences averaging 28 kb in length.

Another consideration is the MES induction efficiency. In this study, we used plasmid DNA vectors for transfecting the Cas3/Cascade/crRNA expression, together with the SSA reporter vectors. We anticipate investigating other methods to deliver Cas3 into cells that might facilitate the overall genome editing efficiency of the Cas3 system.

Finally, we could not show the functionality of the recovered dystrophin protein, such as interaction with the dystrophin-associated protein complex, in the study. We tried immunostaining with β-dystroglycan but failed to detect the signal (data not shown), presumably because of the immature nature of our MYOD1-overexpression-based skeletal muscle differentiation, compared with primary adult skeletal muscle. In the future, more advanced phenotypic assays will be necessary to show that our dual-Cas3-mediated MES approach is still efficient in myotubes after the differentiation of iPSCs.

Nevertheless, we believe the genomic MES-induced iPSCs would be a cell source for future autologous cell therapy applications by differentiating the iPSCs into muscle cells or muscle progenitors to supplement the damaged muscle tissues in DMD patients (Zhao et al., 2020).

In summary, this study demonstrated that the dual CRISPR-Cas3 system is a powerful tool to induce genomic MES by deleting several hundred kilobases in patient-derived iPSCs. This is the first study to show the feasibility of Cas3 as a potential tool for multi-exon skipping in DMD. We expect this will enlighten new ways to treat DMD patients and other genetic disorders that require extensive deletions.

EXPERIMENTAL PROCEDURES

Resource availability

Corresponding author

Further information and requests for resources and reagents should be directed to and will be fulfilled by the corresponding author, Akitsu Hotta (akitsu.hotta@cira.kyoto-u.ac.jp).

Materials availability

Plasmid DNA vectors are deposited in Addgene (https://www.addgene.org/): Cas3/Cascade expression vector pPV-Dual_promoter-EF1α-2xNLS-Cascade+Cas3-P (RD) (Addgene ID: 204619),

crRNA expression vector pPV-C1-crRNA(DMD#20_DMD#23)-EF1a-BA (Addgene ID: 204620), SSA reporter vector pPV-EF1a-EGxxFP(DMDex45_10)-iP-A (Addgene ID: 204621), pPV-EF1α-mRxxFP1(DMDex55_10)-iP-A (Addgene ID: 204622), crRNA cloning vector pPV-C1-crRNA-cloning-EF1a-BA (Addgene ID: 204623), SSA cloning vectors pPV-EF1a-EGxxFP-iP-A (Addgene ID: 204624), and pPV-EF1a-mRxxFP1(AfeI)-iP-A (Addgene ID: 204625).

DMD patient derived iPSC clones CiRA00646 (HPS3945) and CiRA00458 (HPS4305) are deposited in RIKEN BRC. MES-induced iPSC clones (CiRA00458 Δ45-55 #6–10, CiRA00646 Δ45-55 #9) will be deposited in RIKEN BRC https://cell.brc.riken.jp/.

Data and code availability

Data can be requested from the corresponding author. The raw human sequencing data or patient data may be restricted due to the legal restrictions in Japan and the consent of the patients.

Ethical approval

The establishment and use of patient-derived iPSCs were approved by the Ethics Committee of the Graduate School of Medicine, Kyoto University, and Kyoto University Hospital (approval numbers R0091 and G259). All patient information was kept anonymous, and written informed consent was obtained.

Transduction of CRISPR-Cas3 and CRISPR-Cas9

For transduction of iPSCs, 3×10^5 cells were seeded in a well of a 12-well plate coated with iMatrix-511 silk. After 24 h, the cells were transfected with 1,000 ng of CRISPR-Cas3 expression plasmid (pPV-Dual_promoter-EF1α-2xNLS-Cascade+Cas3-iP [RD]) and 500 ng of crRNA expression plasmids (pBSIIKS-U6 or pPV-U6 vectors) by 4 μL of Lipofectamine Stem (Thermo Fisher Scientific). When applying the SSA reporter selection, we added 500 ng of SSA plasmid vectors. 1 day after the transfection, the culture medium was replaced with a fresh medium containing 1 μg/mL of puromycin. After selection with puromycin for 2 days, the cells were harvested for genomic DNA extraction or FACS (fluorescence-activated cell sorting) analysis.

For transduction of HEK293T cells, 2×10^5 cells were seeded in a well of a 12-well plate. The next day, the cells were transfected with plasmid vectors using Lipofectamine 2000 (Thermo Fisher Scientific).

SSA reporter-mediated cell sorting

The iPSCs transfected with the SSA reporter vectors were detached from a culture plate by incubating with 0.5 × TrypLE Select for 10 min at 37°C and then dissociated with PBS containing 2% FBS and 10 μM Y-27632. Similarly, HEK293T cells were detached from a plate by 0.25% Trypsin for 1 min at 37°C and dissociated with PBS containing 2% FBS. The cell suspension was passed through a 45-μm-pore cell strainer (Becton, Dickinson and Company) to remove cell clumps. Then, the fluorescence signals of EGFP and mRFP were detected by a BD FACSAria II, and the data were analyzed by FlowJo software (Becton, Dickinson and Company).

To isolate subclones of iPSCs with successful genome editing, we performed single-cell sorting into a well of a 96-well plate (precoated with iMatrix-511 silk) by using BD FACSAria II (BD) cell sorter. The sorted cells were cultured in StemFit AK02N medium containing Y-27632 for approximately 2 weeks. Then, single iPSC colonies were transferred into a new 24-well plate coated with iMatrix-511 silk. After reaching semi-confluency, half of the cells were cryo-preserved, and the other half were harvested for genomic DNA extraction. The target region was PCR amplified for genotyping using primers in Table S3. Sanger sequencing was performed to analyze deletion patterns.

Quantification of genomic deletion by ddPCR assays

To detect the copy number loss of the genomic DNA, we designed ddPCR probes and primer pairs using Primer3Plus (https://www.bioinformatics.nl/cgi-bin/primer3plus/primer3plus.cgi) software. To detect the target genomic region (i.e., *dystrophin* exon 44–55 locus), we used TaqMan hydrolysis probes labeled with FAM fluorophores. Seven target probes were designed to be approximately equally spaced (every 57 kb) in the exon 45–55 region. Reference probes labeled with HEX fluorophores and primers were designed at the *dystrophin* exon 7 region, which is 160 kb distal from the nearest Cas3/Cas9 target site. All the primer pairs and probes are shown in Table S2.

Isolated genomic DNA (1 μg) was first digested by the EcoRI restriction enzyme (TaKaRa) with H buffer at 37°C overnight. Next, ddPCR reaction mixes (total 22 μL) were prepared by mixing 11 μL of Supermix for the Probes (no dUTP) (Bio-Rad Laboratories), 1 μL of 20 μM target forward and reverse primer pair each, and 0.6 μL of 10 μM target probe, 1 μL of 20 μM reference forward and reverse primer pair each, and 0.6 μL of 10 μM reference probe and 132 ng of EcoRI-digested genomic DNA, and the total volume was adjusted to 22 μL by ultrapure water. Oil droplets were generated using QX200 Automated Droplet Generator (Bio-Rad Laboratories) and run on a C1000 Thermal Cycler with a deep-well block (Bio-Rad Laboratories). All ddPCR reactions were run under the following three-step thermal conditions: step 1, 95°C for 10 min; step 2, 94°C for 30 s; step 3, 60°C for 2 min; repeat steps 2 and 3 for 40 times; step 4, 98°C for 10 min; and step 5, store at 4°C. After PCR reactions, droplets were analyzed by the Bio-Rad QX200 Droplet Reader and QuantaSoft Pro Software.

Statistical analysis

To compare between two groups, two-tailed, unpaired Student's t tests were performed using Prism 8 software. To compare among three groups or more, multiple t tests and Tukey's tests were performed by using Prism 8 software. Fisher's exact test was performed by using Prism 8 software.

SUPPLEMENTAL INFORMATION

Supplemental information can be found online at https://doi.org/10.1016/j.stemcr.2023.07.007.

ACKNOWLEDGMENTS

We thank Dr. Tomoji Mashimo for providing the original CRISPR-Cas3 expression vectors and Dr. Knut Woltjen for providing the *MYOD1* expression vector. We also thank Dr. Kanae Mitsunaga for supporting the FACS experiments. We are grateful to Jun Otomo and Tomoya Uchimura for their advice on skeletal muscle differentiation and protein analysis. We thank Dr. Matthew

Beucler for his critical reading of the manuscript. We thank Drs. Noriyuki Tsumaki and Koji Eto for providing scientific advice. This study was partly supported by the following funds: Japan Agency for Medical Research and Development (AMED) grants (JP17bm0804005, JP13bm0104001, JP17ek0109293, and JP21am0401016), Japan Society for the Promotion of Science (JSPS) KAKENHI grants (21J13593, 22H02601, 21K15361), iPS Cell Research Fund, National Center of Neurology and Psychiatry (NCNP) grant (no. 28-6), and JSPS WISE program "The Graduate Program for Medical Innovation (MIP)".

AUTHOR CONTRIBUTIONS

Conceptualization: Y.Kita and A.H.; methodology: Y.O., and Y.Kojima; investigation: Y.Kita, Y.O., Y.N., J.L., U.B., N.O., and A.I.; supervision: Y.Kojima and A.H.; writing—original draft: Y.Kita, Y.Kojima, and A.H.; writing—review & editing: Y.Kita, Y.Kojima, and A.H.

CONFLICT OF INTERESTS

A.H. is a scientific adviser of C4U. Y.Kita, Y.Kojima, and A.H. have submitted a patent regarding this study.

Received: October 3, 2022
Revised: July 30, 2023
Accepted: July 31, 2023
Published: August 24, 2023

REFERENCES

Amoasii, L., Long, C., Li, H., Mireault, A.A., Shelton, J.M., Sanchez-Ortiz, E., McAnally, J.R., Bhattacharyya, S., Schmidt, F., Grimm, D., et al. (2017). Single-cut genome editing restores dystrophin expression in a new mouse model of muscular dystrophy. Sci. Transl. Med. *9*, eaan8081.

Aoki, Y., Yokota, T., Nagata, T., Nakamura, A., Tanihata, J., Saito, T., Duguez, S.M.R., Nagaraju, K., Hoffman, E.P., Partridge, T., and Takeda, S. (2012). Bodywide skipping of exons 45-55 in dystrophic mdx52 mice by systemic antisense delivery. Proc. Natl. Acad. Sci. USA *109*, 13763–13768.

Bladen, C.L., Salgado, D., Monges, S., Foncuberta, M.E., Kekou, K., Kosma, K., Dawkins, H., Lamont, L., Roy, A.J., Chamova, T., et al. (2015). The TREAT-NMD DMD Global Database: analysis of more than 7,000 Duchenne muscular dystrophy mutations. Hum. Mutat. *36*, 395–402.

Béroud, C., Tuffery-Giraud, S., Matsuo, M., Hamroun, D., Humbertclaude, V., Monnier, N., Moizard, M.P., Voelckel, M.A., Calemard, L.M., Boisseau, P., et al. (2007). Multiexon skipping leading to an artificial DMD protein lacking amino acids from exons 45 through 55 could rescue up to 63% of patients with Duchenne muscular dystrophy. Hum. Mutat. *28*, 196–202.

Cameron, P., Coons, M.M., Klompe, S.E., Lied, A.M., Smith, S.C., Vidal, B., Donohoue, P.D., Rotstein, T., Kohrs, B.W., Nyer, D.B., et al. (2019). Harnessing type I CRISPR-Cas systems for genome engineering in human cells. Nat. Biotechnol. *37*, 1471–1477.

Canver, M.C., Bauer, D.E., Dass, A., Yien, Y.Y., Chung, J., Masuda, T., Maeda, T., Paw, B.H., and Orkin, S.H. (2014). Characterization of genomic deletion efficiency mediated by clustered regularly interspaced short palindromic repeats (CRISPR)/Cas9 nuclease system in mammalian cells. J. Biol. Chem. *289*, 21312–21324.

Choi, J., Chen, W., Suiter, C.C., Lee, C., Chardon, F.M., Yang, W., Leith, A., Daza, R.M., Martin, B., and Shendure, J. (2022). Precise genomic deletions using paired prime editing. Nat. Biotechnol. *40*, 218–226.

Dolan, A.E., Hou, Z., Xiao, Y., Gramelspacher, M.J., Heo, J., Howden, S.E., Freddolino, P.L., Ke, A., and Zhang, Y. (2019). Introducing a Spectrum of Long-Range Genomic Deletions in Human Embryonic Stem Cells Using Type I CRISPR-Cas. Mol. Cell *74*, 936–950.e5.

Doudna, J.A. (2020). The promise and challenge of therapeutic genome editing. Nature *578*, 229–236.

Echigoya, Y., Lim, K.R.Q., Nakamura, A., and Yokota, T. (2018). Multiple Exon Skipping in the Duchenne Muscular Dystrophy Hot Spots: Prospects and Challenges. J. Pers. Med. *8*, 41.

El Refaey, M., Xu, L., Gao, Y., Canan, B.D., Adesanya, T.M.A., Warner, S.C., Akagi, K., Symer, D.E., Mohler, P.J., Ma, J., et al. (2017). In Vivo Genome Editing Restores Dystrophin Expression and Cardiac Function in Dystrophic Mice. Circ. Res. *121*, 923–929.

Fairclough, R.J., Wood, M.J., and Davies, K.E. (2013). Therapy for Duchenne muscular dystrophy: renewed optimism from genetic approaches. Nat. Rev. Genet. *14*, 373–378.

Ferreiro, V., Giliberto, F., Muñiz, G.M.N., Francipane, L., Marzese, D.M., Mampel, A., Roqué, M., Frechtel, G.D., and Szijan, I. (2009). Asymptomatic Becker muscular dystrophy in a family with a multiexon deletion. Muscle Nerve *39*, 239–243.

Gee, P., Lung, M.S.Y., Okuzaki, Y., Sasakawa, N., Iguchi, T., Makita, Y., Hozumi, H., Miura, Y., Yang, L.F., Iwasaki, M., et al. (2020). Extracellular nanovesicles for packaging of CRISPR-Cas9 protein and sgRNA to induce therapeutic exon skipping. Nat. Commun. *11*, 1334.

Jiang, T., Zhang, X.O., Weng, Z., and Xue, W. (2022). Deletion and replacement of long genomic sequences using prime editing. Nat. Biotechnol. *40*, 227–234.

Kagita, A., Lung, M.S.Y., Xu, H., Kita, Y., Sasakawa, N., Iguchi, T., Ono, M., Wang, X.H., Gee, P., and Hotta, A. (2021). Efficient ssODN-Mediated Targeting by Avoiding Cellular Inhibitory RNAs through Precomplexed CRISPR-Cas9/sgRNA Ribonucleoprotein. Stem Cell Rep. *16*, 985–996.

Kenjo, E., Hozumi, H., Makita, Y., Iwabuchi, K.A., Fujimoto, N., Matsumoto, S., Kimura, M., Amano, Y., Ifuku, M., Naoe, Y., et al. (2021). Low immunogenicity of LNP allows repeated administrations of CRISPR-Cas9 mRNA into skeletal muscle in mice. Nat. Commun. *12*, 7101.

Koenig, M., Monaco, A.P., and Kunkel, L.M. (1988). The complete sequence of dystrophin predicts a rod-shaped cytoskeletal protein. Cell *53*, 219–228.

Koo, T., Lu-Nguyen, N.B., Malerba, A., Kim, E., Kim, D., Cappellari, O., Cho, H.Y., Dickson, G., Popplewell, L., and Kim, J.S. (2018). Functional Rescue of Dystrophin Deficiency in Mice Caused by Frameshift Mutations Using Campylobacter jejuni Cas9. Mol. Ther. *26*, 1529–1538.

Kudoh, H., Ikeda, H., Kakitani, M., Ueda, A., Hayasaka, M., Tomizuka, K., and Hanaoka, K. (2005). A new model mouse for Duchenne muscular dystrophy produced by 2.4 Mb deletion of dystrophin gene using Cre-loxP recombination system. Biochem. Biophys. Res. Commun. *328*, 507–516.

Li, H.L., Fujimoto, N., Sasakawa, N., Shirai, S., Ohkame, T., Sakuma, T., Tanaka, M., Amano, N., Watanabe, A., Sakurai, H., et al. (2015). Precise correction of the dystrophin gene in duchenne muscular dystrophy patient induced pluripotent stem cells by TALEN and CRISPR-Cas9. Stem Cell Rep. *4*, 143–154.

Loeff, L., Brouns, S.J.J., and Joo, C. (2018). Repetitive DNA Reeling by the Cascade-Cas3 Complex in Nucleotide Unwinding Steps. Mol. Cell *70*, 385–394.e3.

Long, C., Amoasii, L., Mireault, A.A., McAnally, J.R., Li, H., Sanchez-Ortiz, E., Bhattacharyya, S., Shelton, J.M., Bassel-Duby, R., and Olson, E.N. (2016). Postnatal genome editing partially restores dystrophin expression in a mouse model of muscular dystrophy. Science *351*, 400–403.

Martins-Taylor, K., Nisler, B.S., Taapken, S.M., Compton, T., Crandall, L., Montgomery, K.D., Lalande, M., and Xu, R.H. (2011). Recurrent copy number variations in human induced pluripotent stem cells. Nat. Biotechnol. *29*, 488–491.

Mashiko, D., Fujihara, Y., Satouh, Y., Miyata, H., Isotani, A., and Ikawa, M. (2013). Generation of mutant mice by pronuclear injection of circular plasmid expressing Cas9 and single guided RNA. Sci. Rep. *3*, 3355.

Mercuri, E., Bönnemann, C.G., and Muntoni, F. (2019). Muscular dystrophies. Lancet *394*, 2025–2038.

Min, Y.L., Li, H., Rodriguez-Caycedo, C., Mireault, A.A., Huang, J., Shelton, J.M., McAnally, J.R., Amoasii, L., Mammen, P.P.A., Bassel-Duby, R., and Olson, E.N. (2019). CRISPR-Cas9 corrects Duchenne muscular dystrophy exon 44 deletion mutations in mice and human cells. Sci. Adv. *5*, eaav4324.

Morisaka, H., Yoshimi, K., Okuzaki, Y., Gee, P., Kunihiro, Y., Sonpho, E., Xu, H., Sasakawa, N., Naito, Y., Nakada, S., et al. (2019). CRISPR-Cas3 induces broad and unidirectional genome editing in human cells. Nat. Commun. *10*, 5302.

Muntoni, F., Torelli, S., and Ferlini, A. (2003). Dystrophin and mutations: one gene, several proteins, multiple phenotypes. Lancet Neurol. *2*, 731–740.

Nakamura, A., Yoshida, K., Fukushima, K., Ueda, H., Urasawa, N., Koyama, J., Yazaki, Y., Yazaki, M., Sakai, T., Haruta, S., et al. (2008). Follow-up of three patients with a large in-frame deletion of exons 45-55 in the Duchenne muscular dystrophy (DMD) gene. J. Clin. Neurosci. *15*, 757–763.

Nelson, C.E., Hakim, C.H., Ousterout, D.G., Thakore, P.I., Moreb, E.A., Castellanos Rivera, R.M., Madhavan, S., Pan, X., Ran, F.A., Yan, W.X., et al. (2016). In vivo genome editing improves muscle function in a mouse model of Duchenne muscular dystrophy. Science *351*, 403–407.

Ohno, T., Akase, T., Kono, S., Kurasawa, H., Takashima, T., Kaneko, S., and Aizawa, Y. (2022). Biallelic and gene-wide genomic substitution for endogenous intron and retroelement mutagenesis in human cells. Nat. Commun. *13*, 4219.

Ousterout, D.G., Kabadi, A.M., Thakore, P.I., Majoros, W.H., Reddy, T.E., and Gersbach, C.A. (2015). Multiplex CRISPR/Cas9-based genome editing for correction of dystrophin mutations that cause Duchenne muscular dystrophy. Nat. Commun. *6*, 6244.

Roshmi, R.R., and Yokota, T. (2021). Pharmacological Profile of Viltolarsen for the Treatment of Duchenne Muscular Dystrophy: A Japanese Experience. Clin. Pharmacol. *13*, 235–242.

Saifullah, Motohashi, N., Tsukahara, T., and Aoki, Y. (2022). Development of Therapeutic RNA Manipulation for Muscular Dystrophy. Front. Genome Ed. *4*, 863651.

Tabebordbar, M., Zhu, K., Cheng, J.K.W., Chew, W.L., Widrick, J.J., Yan, W.X., Maesner, C., Wu, E.Y., Xiao, R., Ran, F.A., et al. (2016). In vivo gene editing in dystrophic mouse muscle and muscle stem cells. Science *351*, 407–411.

Xu, L., Lau, Y.S., Gao, Y., Li, H., and Han, R. (2019). Life-Long AAV-Mediated CRISPR Genome Editing in Dystrophic Heart Improves Cardiomyopathy without Causing Serious Lesions in mdx Mice. Mol. Ther. *27*, 1407–1414.

Yoshimi, K., Kunihiro, Y., Kaneko, T., Nagahora, H., Voigt, B., and Mashimo, T. (2016). ssODN-mediated knock-in with CRISPR-Cas for large genomic regions in zygotes. Nat. Commun. *7*, 10431.

Young, C.S., Hicks, M.R., Ermolova, N.V., Nakano, H., Jan, M., Younesi, S., Karumbayaram, S., Kumagai-Cresse, C., Wang, D., Zack, J.A., et al. (2016). A Single CRISPR-Cas9 Deletion Strategy that Targets the Majority of DMD Patients Restores Dystrophin Function in hiPSC-Derived Muscle Cells. Cell Stem Cell *18*, 533–540.

Zhang, Y., Li, H., Min, Y.L., Sanchez-Ortiz, E., Huang, J., Mireault, A.A., Shelton, J.M., Kim, J., Mammen, P.P.A., Bassel-Duby, R., and Olson, E.N. (2020). Enhanced CRISPR-Cas9 correction of Duchenne muscular dystrophy in mice by a self-complementary AAV delivery system. Sci. Adv. *6*, eaay6812.

Zhao, M., Tazumi, A., Takayama, S., Takenaka-Ninagawa, N., Nalbandian, M., Nagai, M., Nakamura, Y., Nakasa, M., Watanabe, A., Ikeya, M., et al. (2020). Induced Fetal Human Muscle Stem Cells with High Therapeutic Potential in a Mouse Muscular Dystrophy Model. Stem Cell Rep. *15*, 80–94.

Stem Cell Reports, Volume *18*

Supplemental Information

Dual CRISPR-Cas3 system for inducing multi-exon skipping in DMD patient-derived iPSCs

Yuto Kita, Yuya Okuzaki, Youichi Naoe, Joseph Lee, Uikyu Bang, Natsumi Okawa, Akane Ichiki, Tatsuya Jonouchi, Hidetoshi Sakurai, Yusuke Kojima, and Akitsu Hotta

Supplemental information

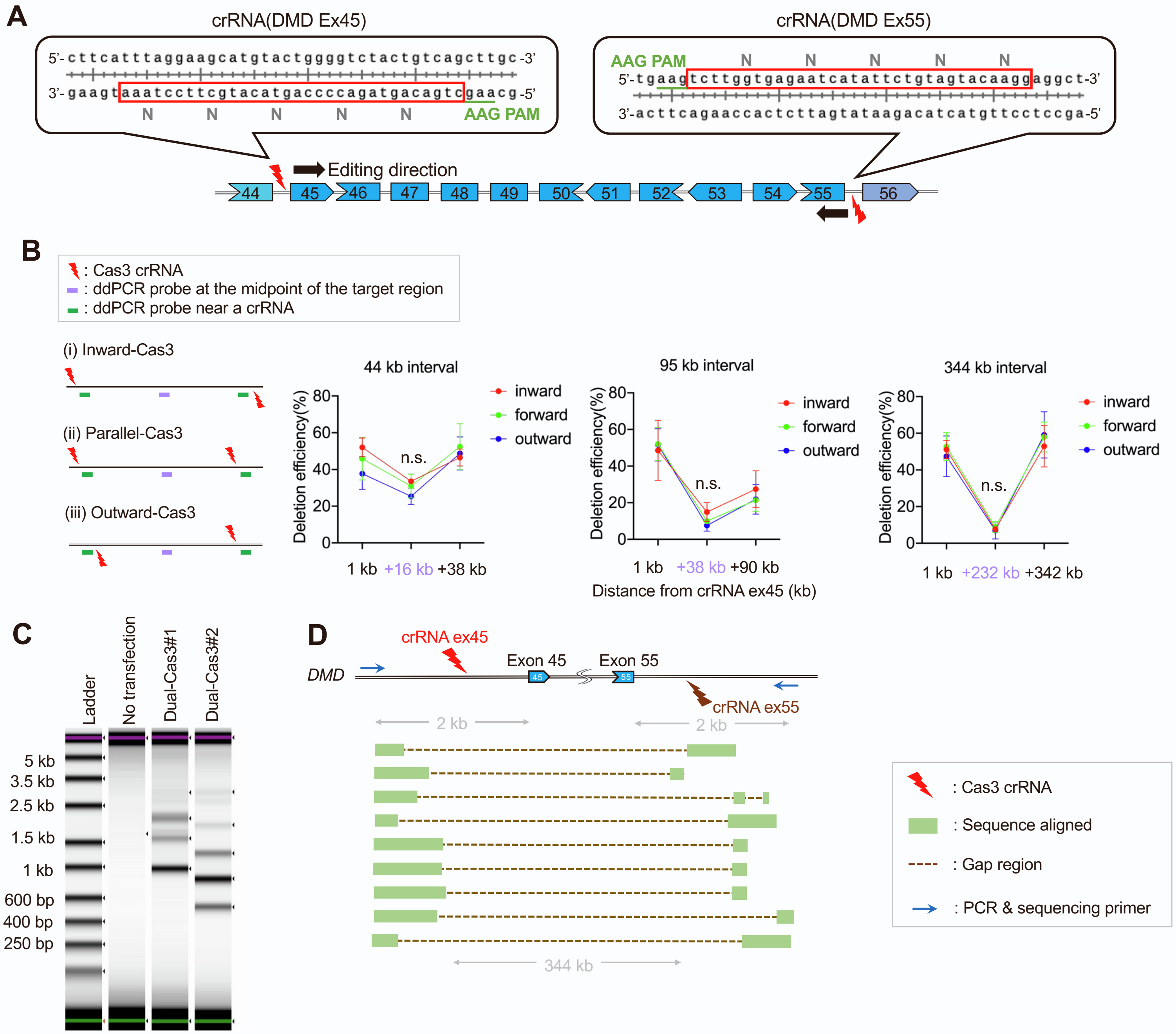

Supplemental Figure 1. Analyses and application of a large deletion by the dual-Cas3 system.
(A) The target sequences of crRNAs (DMD Ex45 and Ex55) used for the dual-Cas3 approach in Figure 1A (iv). CRISPR-Cas3 crRNA recognizes AAG (canonical PAM) + 32 nt target sequences, in which every 6 nt position is not involved in sequence recognition (indicated by “N”).
(B) Comparison of the deletion efficiency among the inward, parallel, and outward orientation of dual crRNAs. Similar to Figure 1A (ii), (iii) and (iv), genome editing was performed in HEK293T cells using pairs of inward, parallel, and outward crRNAs. DNA copy number losses were measured by ddPCR at indicated positions. Data were represented as means ± SD from independent experiments (n = 3). ANOVA with the Tukey test was used to calculate *p*-values. n.s.: not significant.
(C) CRISPR-Cas3/Cascade expression vector with puromycin resistance cassette and two crRNA expression vectors targeting dystrophin exons 45 and 55 were transfected into DMD patient-derived iPSCs (FF12020). After one day of puromycin selection, the dystrophin exon 45-55 region was amplified by PCR and analyzed by TapeStation D5000 DNA tape.
(D) PCR products from (C) were subjected to Sanger sequencing after TA cloning. Sequencing results were aligned to the dystrophin gene locus shown as the green box. Identified deletion patterns with defined breakpoint(s) are indicated as the dotted line.

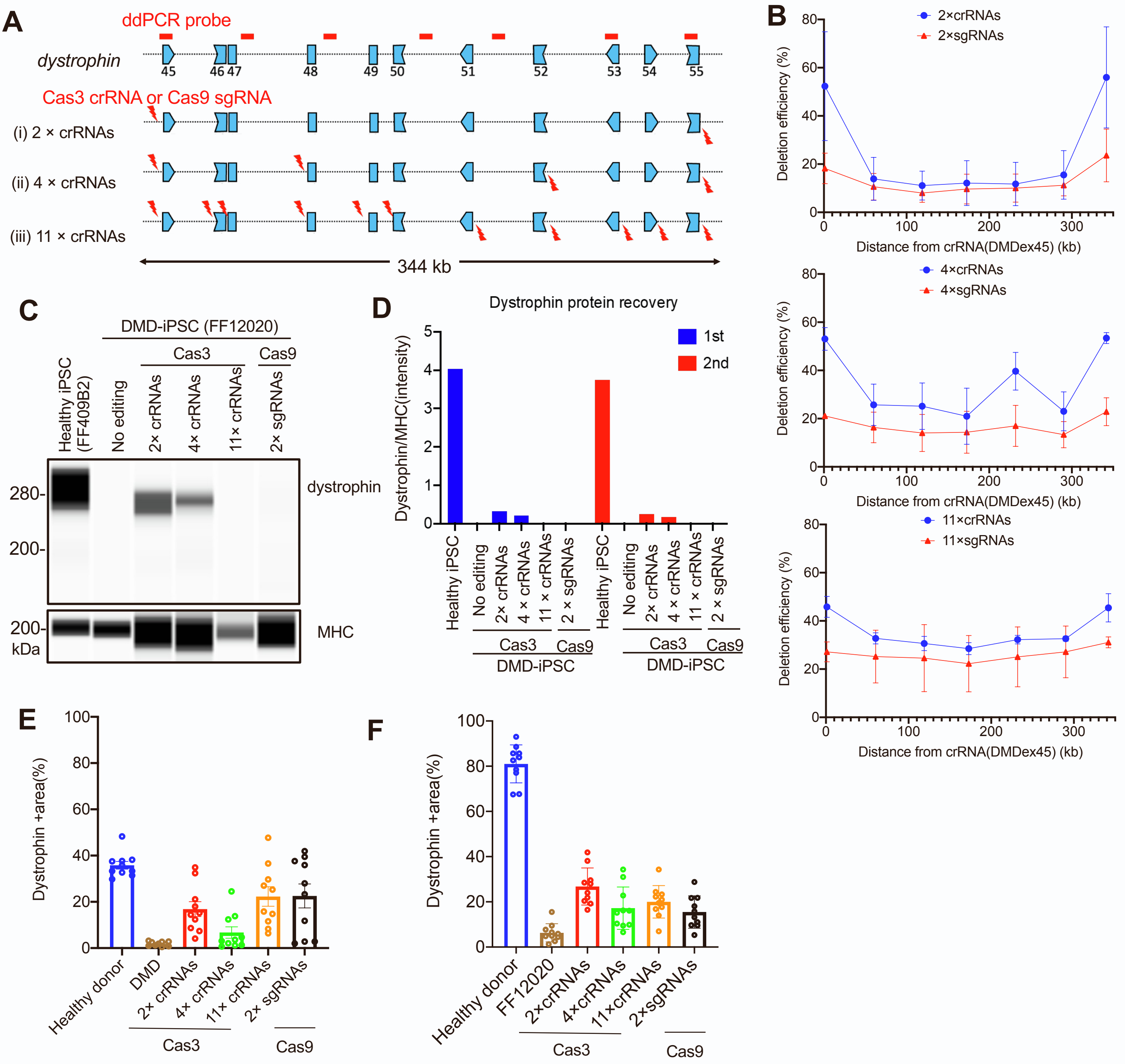

Supplemental Figure 2. Induction of multi-exon skipping by multiplexed crRNAs

(A) Schematic of designed Cas3 crRNAs/Cas9 sgRNAs and ddPCR probes for the DMD multi-exon skipping using multiplexed crRNAs. Positions of Cas3 crRNAs/Cas9 sgRNAs are shown as red thunder marks where the edge of the thunder mark indicates the PAM side. Positions of ddPCR probes are indicated as red horizontal bars.

(B) ddPCR analyses after multiplexed Cas3/Cas9 genome editing in HEK293T cells. DNA copy number losses were measured by ddPCR using probes indicated in (A). Data were represented as means ± S.D. from independent experiments (n = 3).

(C) Recovery of dystrophin protein after multiplexed Cas3 genome editing. Multiplexed Cas3 editing followed by two-color SSA vector enrichment for crRNA(Ex45) and crRNA(Ex55) was performed in FF12020 DMD-iPSC line. After skeletal muscle differentiation of the bulk cell samples, dystrophin and MHC (myosin heavy chain) proteins were detected by the Wes system. Data from two independent experiments are shown.

(D) Quantification of dystrophin protein amount normalized with MHC protein signal was calculated from the two independent Wes experiments.

(E, F) Immunocytochemical staining to assess recovery of dystrophin protein in FF12020 DMD-iPSC line. Genome-edited cells by multiplexed Cas3 were enriched with the two-color SSA vectors and subjected to skeletal muscle differentiation similar to (C). Immunocytochemical staining experiments for myosin heavy chain (MYH) and dystrophin proteins were performed and the results from two independent experiments are shown. For controls, healthy donor-derived iPSCs (FF409B2) and unedited parental FF12020 DMD-iPSC were used. Quantification of dystrophin-positive area within MHC-positive skeletal muscle cells. The area of the dystrophin-positive and the MHC-positive region were analyzed by ImageJ software. Data are represented as means ± S.D. from 10 images.

A

iPSC clone ID	Age at biopsy	Gender	DMD deletion analysis	Deletion pattern	Cell source
FF12020	8 y.o.	Male	MLPA	Exon 46, 47 deletion	Skin fibroblasts
CiRA00458	13 y.o.	Male	MLPA	Exon 51-53 deletion	Peripheral blood mononuclear cells
CiRA00646	20 y.o.	Male	MLPA	Exon 48-52 deletion	Skin fibroblasts

B

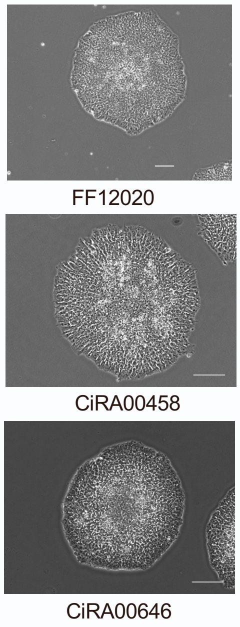

C

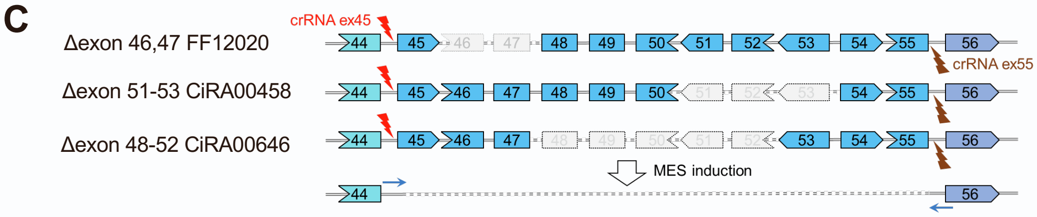

D

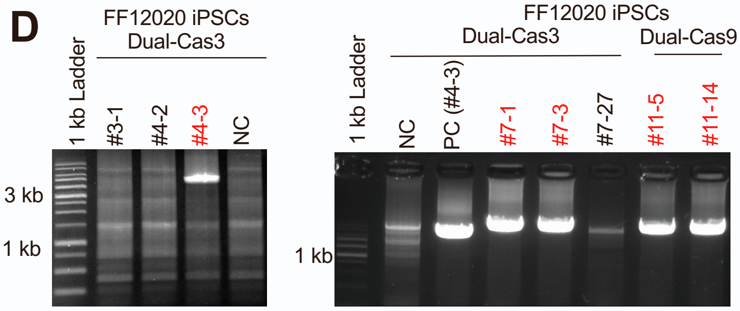

E

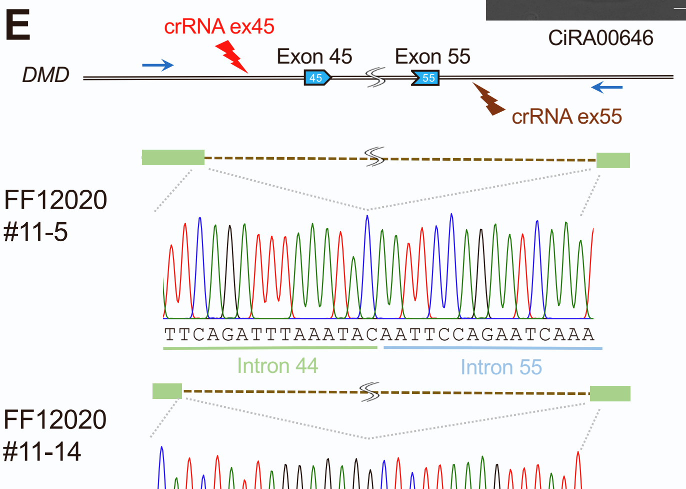

F

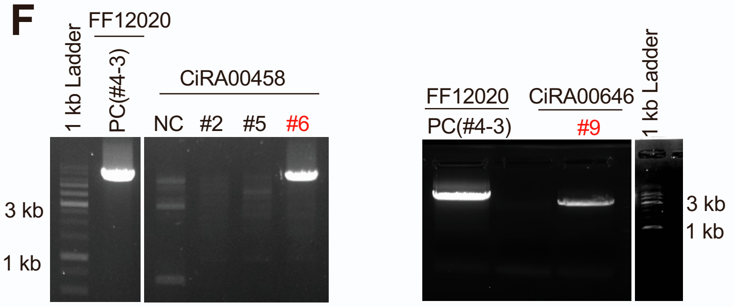

G

Summary of genotyping results

Cell line	SSA enrichment	Trial	Exon 45-55 large deletion	Total clone#
FF12020	No	1st trial	0 (0%)	55
		2nd trial	0 (0%)	65
	Yes	1st trial	1 (16.7%)	6
		2nd trial	2 (6.5%)	33
		3rd trial	18 (30.5%)	59
CiRA00458	Yes	1st trial	1 (33.3%)	3
CiRA00646	Yes	1st trial	1 (16.7%)	6

H

Summary of detailed genotyping for FF12020 3rd trial with SSA enrichment

Cell line	SSA enrichment	Trial	Exon 45-55 large deletion	Small deletions at Exon 45 & 55	Small deletion at only Exon 45	Small deletion at only Exon 55	Other	Unedited	Total clone#
FF12020	Yes	3rd trial	18 (30.5%)	4 (6.8%)	16 (27.1%)	1 (1.7%)	1 (1.7%)	19 (32.2%)	59

Supplemental Figure 3. Establishment of DMD patient-derived iPSCs and genotyping of subclones after MES induction by the dual-Cas3 system.

(A) DMD patients' information related to establishing the patient-derived iPSCs.
(B) Morphology of iPSC colony cultured on feeder-free and xeno-free culture condition. Scale bar : 100 μm.
(C) Exon deletion patterns of the three DMD patient iPSCs. For genotyping after dual-Cas3 treatment, we used the PCR primers that flank the 340 kb target region. PCR amplification happens only when a large deletion is induced.
(D) Genotyping was performed after dual-Cas3 treatment and subcloning in three DMD patient-derived iPS cell lines (FF12020, CiRA00458, and CiRA00646). As a result, we identified three subclones #4-3, #7-1, and #7-3 after dual-Cas3 treatment. and 2 subclones (#11-5, #11-14) after dual-Cas9 treatment.
(E) The deletion pattern of subclones #11-5, and #11-14 was analyzed by Sanger sequencing.
(F) Subcloning and genotyping were also performed in CiRA00458 and CiRA00646 DMD-iPSC lines and subclones #6 and #9 were established, respectively.
(G) Summary of single-cell cloning and genotyping. Dual-Cas3 genome editing was performed in FF12020, CiRA00458, and CiRA00646 iPSCs. Isolated subclones were genotyped by the amplification size of junction PCR to detect large Ex45-55 deletions indicated in (C) and (D).
(H) Detailed genotyping results in the 3rd subcloning trial of the FF12020 DMD-iPSCs with the SSA vector enrichment. PCR was performed on exon 45, exon 55, and intron 50 regions separately to various genome editing patterns.

A

CNV_ID	Position	crRNA binding site (PAM)	Mismatch (nt)	Strand	Distance (kb)
32	chr8:58,511,328-62,603,471	GTGCTGgCCtgAGAAgaCAaGACcaGCTTCCTCAA	8	-	908 & 3184

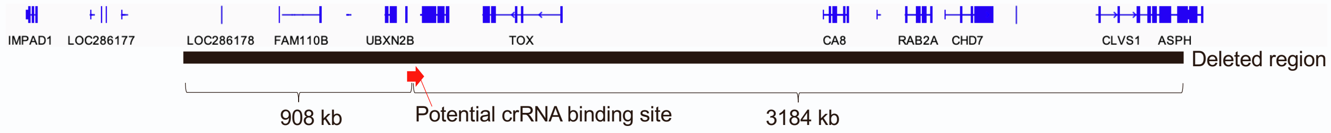

B

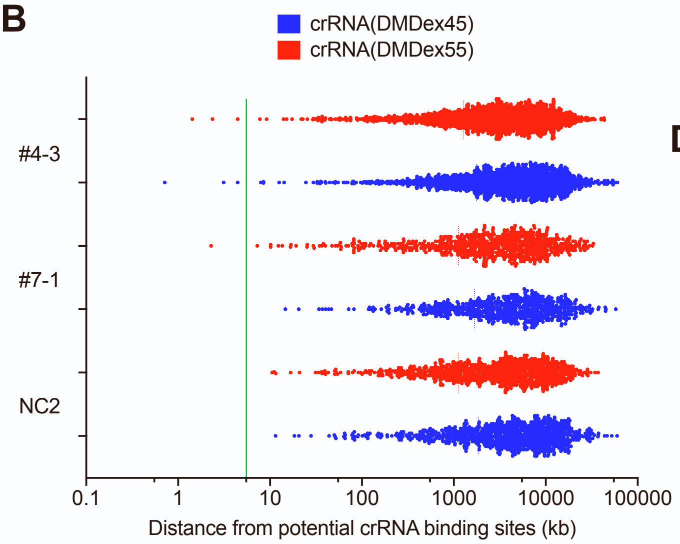

C

#4-3	#7-1	Position	Ref	Alt	Nearest crRNA	crRNA binding site	Mismatch	Distance from the indels/SNVs	Exist in the Cas3 cleavage direction?
x	-	chr4:116904319	C	T	ex45	chr4:116903572_116903603	8 bp	0.716 kb	No
x	-	chr10:119402039	A	C	ex45	chr10:119398878_119398909	8 bp	3.127 kb	Yes
x	-	chr17:79314829	C	T	ex45	chr17:79310353_79310384	8 bp	4.445 kb	No
x	-	chr2:229669588	-	AG	ex55	chr2:229671019_229671050	8 bp	1.428 kb	Yes
-	x	chr:4175456696	G	T	ex55	chr4:175458986_175459017	8 bp	2.287 kb	Yes
x	-	chr15:95919559	C	A	ex55	chr15:95917160_95917191	8 bp	2.368 kb	No
x	-	chr17:36013899	T	-	ex55	chr17:36018355_36018386	7 bp	4.453 kb	No

D

Sample ID	Total No. of Indels/SNVs detected	No. of Indels/SNVs detected within 5 kb from the potential crRNA binding sites	Fisher's exact test (relative to NC2)
NC2	997	0	
#7-1	717	1	$p = 0.42$
#4-3	2038	6	$p = 0.19$

Supplemental Figure 4. Additional information for the off-target analyses performed by whole genome sequencing.
(A) Detailed information of the 4058 kb CNV containing a potential crRNA (DMD ex45) binding site. In the putative crRNA binding site, the noncanonical PAM motif is shown underbar, and mismatch bases are shown as small letters.
(B) The overall distances between the detected indel/SNV and the closest potential crRNA binding site are plotted, where blue dots represent the distances from the potential binding sites of crRNA ex45, and red dots represent the distances from the crRNA ex55. The vertical green line represents a 5 kb window, where most of the Cas3-induced cleavage happens within 5 kb from the crRNA binding site.
(C) Indels and SNVs detected within a 5 kb window from the potential crRNA binding sites in two MES-induced clones #7-1 and #4-3.
(D) The number of SNV/indels detected in the whole genome and in the 5 kb window from potential crRNA binding sites are shown for the Cas3-untreated clone (NC2) and two MES-induced clones #7-1 and #4-3. By using NC2 as a control, Fisher's exact test was performed to calculate the odds of finding indels/SNVs in the given area (whole genome vs. ± 5 kb from crRNAs).

Supplemental Table 1. List of Cas3 crRNA/Cas9 sgRNA targets

Cas3 crRNA/ Cas9 sgRNA name	Alternative name	PAM	Target Sequence	Used in
crRNA(DMD Ex45)	crRNA(DMD#20)	AAG	ctgacagtagaccccagtacatgcttcctaaa	1-4, S1, S2, S4
crRNA(DMD Ex55)	crRNA(DMD#23)	AAG	tcttggtgagaatcatattctgtagtacaagg	1-4, S1, S2, S4
crRNA(DMD Ex45_1kb)	crRNA_DMDex45_1kb	AAG	Cctgaatctgcggtggcaggaggtctgcaaac	1, S1
crRNA(DMD Ex45_44kb)	crRNA_DMDex45_44kb	AAG	Ctcaagcagacaaatctccagtggataaaggt	1, S1
crRNA(DMD Ex45_95kb)	crRNA_DMDex45_95kb	AAG	acaaaatattacttgttaaagtgtggtaagga	1, S1
crRNA(DMD Ex45_outward)	crRNA_DMD#9	AAG	gaagacttggcctttatttaccaaatgagact	S1
crRNA(DMD Ex47)	crRNA(DMDex45_44kb_outward)	AAG	gaaactgagacaaaggggttaaataccagctc	S1, S2
crRNA(DMD Ex45_90kb_outward)	crRNA_DMDex45_95kb_out_1	AAG	ataagcaaaagtgtctgttttagaaataaaca	S1
crRNA(DMD Ex55_outward)	crRNA_DMDex55_out_1	AAG	aaaatacaaacctagaatcaaaaggaaaagac	S1
crRNA(B2M)	crRNA(B2M#1)	AAG	gccacggagcgagacatctcggcccgaatgct	2
crRNA(DMD Ex46)	crRNA(DMDexon46_1)	AAG	actccatctcaaatacatacatacatacgtac	S2
crRNA(DMD Ex48)	crRNA(DMDexon48_1)	AAG	attgtaaaaggaagtcccttcatgtacaagga	S2
crRNA(DMD Ex49)	crRNA(DMDexon49_1)	AAG	cttattatttattaatgagtcaaacaggcctt	S2
crRNA(DMD Ex50)	crRNA(DMDexon50_1)	AAG	ttcatgcctttccccaggcagccctcattcag	S2
crRNA(DMD Ex51)	crRNA_DMD#25	AAG	ttggcatttatgcaatgccatgttcaaatgaa	S2
crRNA(DMD Ex52)	crRNA(DMDexon52_1)	AAG	acccatctgactagacgctgtgcatattcttt	S2
crRNA(DMD Ex53)	crRNA(DMDexon53_1)	AAG	atcacttcaaaattggtatacgtatttatgta	S2
crRNA(DMD Ex54)	crRNA(DMDexon54_1)	AAG	ccttgtatccttattatacctctcttgatctc	S2
sgRNA(DMD Ex45#3)	sgRNA_DMDexon45#3	NGG	ttgctattgtgtcaaggagt	1, S4
sgRNA(DMD Ex55#1)	sgRNA(DMDexon55#1)	NGG	tcatattctgtagtacaagg	1, S4
sgRNA(DMD Ex45_1kb)	sgRNA_DMD1	NGG	tggtatcttacaggaactcc	1
sgRNA(DMD Ex45_44kb)	sgRNA_DMDex45_44kb	NGG	agaagcaaagacaaggtagt	1
sgRNA(DMD Ex45_95kb)	sgRNA_DMDex45_95kb	NGG	aaatattacttgttaaagtg	1
sgRNA(DMD Ex46)	sgRNA_DMDex46_1	NGG	agggtactggttatacttgg	S2
sgRNA(DMD Ex47)	sgRNA_DMDex47_2	NGG	gggatatgaagtaaaaaatg	S2
sgRNA(DMD Ex48)	sgRNA_DMDex48_2	NGG	aaacttccaaggatgatggt	S2
sgRNA(DMD Ex49)	sgRNA_DMDex49_2	NGG	tgctttaagtgtttaccctt	S2
sgRNA(DMD Ex50)	sgRNA_DMDex50_2	NGG	cagctcaggcagtccctgaa	S2
sgRNA(DMD Ex51)	sgRNA_DMDex51_2	NGG	agataaacttgggctcaggt	S2
sgRNA(DMD Ex52)	sgRNA_DMDex52_1	NGG	gagaatctctgagggaaacc	S2
sgRNA(DMD Ex53)	sgRNA_DMDex53_1	NGG	aggaaggaattaagcccgaa	S2
sgRNA(DMD Ex54)	sgRNA_DMDex54_2	NGG	tagctccctattatatcacg	S2

Supplemental Table 2. Primers used for PCR, Sanger sequencing, and RT-PCR (left column). Primers and probes used for ddPCR experiments (Right column). Related to Experimental Procedures section.

Name	Sequence
YK#87_crRNA_DMDexon45check_rev	acatctgatgtgtgcccatgc
YK#88_crRNA_DMDexon55check_dir	cgcaaatgttgaggttttcagaggc
YK#89_crRNA_DMDexon55check_rev	ggatttggctgaaaatcacattgtctcg
YK#90_crRNA_DMDexon51check_dir	gaggctataaaagccaagaactgacaatg
YK#91_crRNA_DMDexon51check_rev	catttgccagtcagcctggtgttctgc
YK#92_crRNA_DMDexon45check_rev2	ctatggtatacatctgatgtgtgcccatgc
YK#93_crRNA_DMDexon45check_rev3	cttctggcctaccatttgttgatcacc
YK#107_DMDexon45-55check_dir1	cctaccatagctgatgggtaaaatgtaaac
YK#108_DMDexon45-55check_dir2	gaagcttgcagtttcttgagattagaaatagag
YK#109_DMDexon45-55check_rev1	gactggtgtctctattactaagcaatgactgtc
YK#110_DMDexon45-55check_rev2	gcaaatgcttactgaaaccttccatgcatc
YK#113_DMDexon45(45-55)check_rev3	gactccatggtgatgatgagcc
YK#114_DMDexon45(45-55)check_rev4	ctcctccgctcatccttttcactg
YK#115_DMDexon45(45-55)check_rev5	caccacaggctttaacttctgccg
YK#116_DMDexon55(45-55)check_dir3	ggttaagccactggacaaaaacg
YK#117_DMDexon55(45-55)check_dir4	gagtagcctgatcgacactggac
YK#118_DMDexon55(45-55)check_dir5	cctcgggtacactgaaagttatgtg
YK#167_sgRNA(44C4)_check_dir	gccaacgctggtgtgcatgc
YK#168_sgRNA(44C4)_check_rev	gcctggacggagctggtttatc
YK#169_sgRNA(55C3)_check_dir	cttggcgtattgccttctcctg
YK#170_sgRNA(55C3)_check_rev	gttcctcccacatccacacgc
YK#171_sgRNA(DMDexon45)_check_dir	caggagcatcccatcaagatatccc
YK#172_sgRNA(DMDexon55)_check_dir	ctcaacacgcattttggaggag
YK#173_sgRNA(DMDexon55)_check_rev	gctctgaccaggaaaacatttgc
YK#229_crRNA(DMDexon46)_check_dir	gcagaactgctcaactgagcc
YK#230_crRNA(DMDexon46)_check_rev	gccactgagctggacacacg
YK#231_crRNA(DMDexon47)_check_dir	ccctagaacaatgaactcagtc
YK#232_crRNA(DMDexon47)_check_rev	cccactctaacctttggatgaaggg
YK#233_crRNA(DMDexon48)_check_dir	gctctaaggtatgttcagggatgacg
YK#234_crRNA(DMDexon48)_check_rev	cacacagcagtgagtggtgac
YK#235_crRNA(DMDexon49)_check_dir	gcaagagactgtactgggcag
YK#236_crRNA(DMDexon49)_check_rev	gcttgggaggtgagcgtagg
YK#237_crRNA(DMDexon50)_check_dir	ctgccctagggcgttaggac
YK#238_crRNA(DMDexon50)_check_rev	cctgcaagtcaggaagtcggg
YK#239_crRNA(DMDexon52)_check_dir	cgttactctcaccatatgcttgtg
YK#240_crRNA(DMDexon52)_check_rev	gtgcacacaccatctaatgcttatg
YK#241_crRNA(DMDexon53)_check_dir	gctgttccactacctagtggaac
YK#242_crRNA(DMDexon53)_check_rev	gagggttttgatcaagtgccc
YK#243_crRNA(DMDexon54)_check_dir	gagtgatgggtggcttccaag
YK#244_crRNA(DMDexon54)_check_rev	gagaagaaagtccaagccttgcc
YK#297_DMDexon55(45-55)check_dir6	cctgaagtacaaggacgacgg
YK#298_DMDexon55(45-55)check_dir7	cttcagaattaatccgtgcgcc
YK#299_DMDexon55(45-55)check_dir8	cacacacaaaccaccgaaccaaag
YK#300_DMDexon45(45-55)check_rev6	gtagacttagaatggaattctgggc
YK#301_DMDexon45(45-55)check_rev7	gaatgggtcctggtgctgtgcttagc
YK#302_DMDexon45(45-55)check_rev8	ccgcggtatctgttgttcgc
YK414_DMDex55_check_F	cctcccacaaaacatatgacagtg
YK415_DMDex45_check_R	catgaatgagttagaaggcgagaagg
YK416_DMDex45_check_rev10	ggagtcagtcacactaaagagaacac
YK417_DMDex55_check_dir10	gcaagaagacaatcatgtgagtcttg
YK573_DMDex45_40kb_check_F	cagcagcaagctacggtatg
YK574_DMDex45_95kb_check_F	ggtttccagagctttacctgaga
YK580_crRNA_DMDex45_95kb_check_F	ccctgctgccatgtagaatgtg
YK581_crRNA_DMDex45_95kb_check_R	ctcctacactgctccttggc
YK582_sgRNA_DMDex45_40kb_check_R	cacatgtgacggaagagatgg
YK188_DMDexon56_rev2	caggactgcatcatcggaacc
YK378_DMD_E44_cDNA_F	cagtggctaacagaagctgaac
YK379_DMD_E45_cDNA_R	ggcatctgtttttgaggattgc

Name	Sequence
DMDexon7_ddPCR_F	tgtgttttaggccagacc
DMDexon7_ddPCR_R	agcagtggtagtccagaa
DMDexon7_probe	agtcgttgtgtggctgactgctggca
DMDex45_ddPCR_F	ggaactccaggatggcattgg
DMDex45_ddPCR_R	gacagctgtttgcagacct
DMDexon45_probe	tgggaagcctgaatctgcggtggca
DMDin50_ddPCR_F	ccccaggaagggcattaag
DMDin50_ddPCR_R	agttcagatgttgtcccc
DMDin50_probe	cccccatgaccccacacttcccgt
DMDin47_ddPCR_F	ctgacttccctcttggtgac
DMDin47_ddPCR_R	caggaatgagcatgtgacct
DMDintron47_probe	tggcctgaagccgtttcagaacccaca
DMDin48_ddPCR_F	tgcagctttactccacac
DMDin48_ddPCR_R	ggcagttcaagatttgctgg
DMDintron48_probe	cccaggaccctagcggaaagagcagc
DMDin51_ddPCR_F	ctgttgctggatactttcgc
DMDin51_ddPCR_R	cgactcactctcctctcat
DMDintron51_probe	cccaggaccctagcggaaagagcagc
DMDex53_ddPCR_F	caatggctggaagctaagga
DMDex53_ddPCR_R	tgtatagggaccctccttcc
DMDexon53_probe	agcaggtcttaggacaggccagagcca
DMDex55_ddPCR_F	ggctgctttggaagaaactc
DMDex55_ddPCR_R	cagctcttttactcccttgg
DMDexon55_probe	actgcaacagttccccctggacctgga
ddPCR_ex45_16kb_fwd	cccagcttggtcaagcataa
ddPCR_ex45_16kb_rev	agactgcaactttaggccag
ddPCR_ex45_16kb_probe	tcagtaacggtggagacctaggcggca
ddPCR_ex45_38kb_fwd	tcaccacttcagcctctact
ddPCR_ex45_38kb_rev	aggacggtatgaaaagccac
ddPCR_ex45_38kb_probe	accaacaccacgtcccacaccccc
ddPCR_ex45_90kb_fwd	tggtaggaagatgagctggt
ddPCR_ex45_90kb_rev	agtatgggggatcctcttcc
ddPCR_ex45_90kb_probe	ccccacatgtcaagggtggagccagg
ddPCR_ex45_Neg1kb_fwd	acgttgcctcaagttctctg
ddPCR_ex45_Neg1kb_rev	agtgaaaaggatgagcggag
ddPCR_ex45_Neg1kb_probe	cccaggccccaatatacaccgagggc

Supplemental Table 3. List of CNVs detected in WGS. Related to Figure 4.

ID	Type	Location	Size (bp)	NC2	#4-3	#7-1
1	loss	chr1:838,617-1,030,456	191,840	-	○	-
2	gain	chr1:144,810,826-145,298,712	487,887	-	○	-
3	loss	chr2:1,357,914-1,358,836	923	○	-	○
4	loss	chr2:37,258,016-37,278,680	20,665	-	○	-
5	loss	chr2:242,790,469-242,790,961	493	-	-	○
6	loss	chr3:3,412,335-3,412,629	295	○	-	-
7	loss	chr3:4,848,820-4,848,876	57	-	-	○
8	loss	chr3:45,609,905-45,610,321	417	-	-	○
9	loss	chr3:194,384,128-194,384,520	393	-	-	○
10	gain	chr4:54,592,280-54,603,331	11,052	-	○	-
11	loss	chr4:65,719,085-65,719,575	491	-	-	○
12	gain	chr4:92,606,746-93,528,071	921,326	-	○	-
13	loss	chr5:2,748,121-2,760,235	12,115	-	○	-
14	loss	chr5:46,043,702-46,086,649	42,948	-	○	-
15	loss	chr5:46,218,649-46,333,576	114,928	-	○	-
16	loss	chr5:46,333,576-49,443,137	3,109,562	-	○	-
17	gain	chr5:178,012,401-178,012,673	273	-	○	-
18	gain	chr5:180,566,548-180,566,851	304	-	-	○
19	loss	chr6:217,664-382,715	165,052	-	○	-
20	loss	chr6:382,715-3,367,820	2,985,106	-	○	-
21	loss	chr6:29,815,110-29,815,768	659	-	-	○
22	loss	chr6:94,248,198-94,264,157	15,960	-	○	-
23	loss	chr6:141,304,606-141,318,406	13,801	-	○	-
24	gain	chr6:169,240,479-169,240,928	450	-	-	○
25	gain	chr6:169,331,272-169,331,585	314	-	-	○
26	loss	chr7:57,950,847-58,013,552	62,706	-	○	-
27	loss	chr7:61,241,479-61,511,524	270,046	-	○	-
28	loss	chr7:150,463,048-150,463,551	504	○	-	-
29	gain	chr8:20,198,662-20,203,119	4,458	-	○	-
30	loss	chr8:20,198,767-20,203,952	5,186	-	○	-
31	loss	chr8:43,597,003-43,660,505	63,503	-	○	-
32	loss	chr8:58,511,328-62,603,471	4,092,144	-	-	○
33	loss	chr8:91,254,169-91,903,496	649,328	○	-	-
34	loss	chr8:103,441,499-103,441,868	370	-	-	○
35	loss	chr8:123,165,142-123,168,985	3,844	○	-	-
36	loss	chr9:83,269,037-83,271,232	2,196	-	○	-
37	loss	chr10:8,075,816-8,108,417	32,602	-	○	-
38	loss	chr10:47,642,581-47,643,078	498	-	-	○
39	loss	chr10:55,893,688-55,894,134	447	-	-	○
40	loss	chr10:67,032,382-67,032,923	542	○	-	-
41	gain	chr10:134,879,786-134,880,359	574	-	○	-
42	gain	chr11:48,528,012-87,356,743	38,828,732	-	○	-
43	loss	chr11:50,781,651-51,163,742	382,092	-	○	-
44	loss	chr11:51,207,530-51,252,364	44,835	-	○	-
45	loss	chr11:63,527,207-63,540,475	13,269	-	○	-
46	loss	chr12:38,231,554-38,408,805	177,252	-	○	-
47	gain	chr12:132,879,982-132,880,445	464	-	○	-
48	loss	chr13:95,355,676-95,368,107	12,432	-	○	-
49	loss	chr13:110,432,870-110,446,468	13,599	-	○	-
50	loss	chr14:46,590,901-46,621,369	30,469	-	○	-
51	loss	chr14:78,827,811-78,828,585	775	-	-	○
52	loss	chr14:87,857,880-87,858,384	505	-	-	○
53	loss	chr14:104,674,521-104,675,002	482	○	-	-
54	loss	chr15:74,653,286-75,221,457	568,172	-	○	-
55	loss	chr15:74,653,679-74,709,590	55,912	-	○	-
56	gain	chr15:75,221,643-82,903,355	7,681,713	△	○	-
57	gain	chr15:82,903,355-83,167,067	263,713	△	○	-
58	gain	chr15:83,167,067-100,457,023	17,289,957	△	○	-
59	gain	chr15:100,457,023-102,445,015	1,987,993	△	○	-
60	gain	chr16:12,021,477-29,394,630	17,373,154	-	○	-
61	loss	chr16:29,815,073-29,828,813	13,741	-	○	-
62	loss	chr16:71,407,745-71,439,679	31,935	-	○	-
63	loss	chr16:85,639,482-85,652,218	12,737	-	○	-
64	loss	chr16:88,024,857-88,024,916	60	-	○	-
65	loss	chr17:77,804,119-77,821,079	16,961	-	○	-
66	gain	chr19:22,363,444-22,363,870	427	-	○	-
67	loss	chr19:27,833,052-27,870,223	37,172	-	○	-
68	loss	chr19:27,893,812-28,065,242	171,431	-	○	-
69	loss	chr20:20,337,172-20,338,425	1,254	-	○	-
70	gain	chr20:25,582,240-25,582,500	261	-	○	-
71	gain	chr20:29,836,152-30,788,715	952,564	△	○	-
72	loss	chr20:41,624,640-41,634,640	10,001	○	-	-
73	loss	chr20:50,347,622-50,348,035	414	-	○	-
74	loss	chr20:52,318,479-52,318,783	305	-	-	○
75	gain	chr20:55,062,013-55,385,592	323,580	○	-	-
76	loss	chr22:50,305,756-50,418,937	113,182	-	○	-
77	loss	chrX:31,643,677-31,989,563	345,887	-	○	○
78	loss	chrX:50,122,314-50,135,051	12,738	○	-	-
79	loss	chrX:72,358,023-72,375,780	17,758	-	○	-
80	loss	chrX:80,280,719-80,295,147	14,429	-	-	○
81	loss	chrX:112,703,477-112,716,769	13,293	○	-	-
82	loss	chrX:129,369,525-129,383,770	14,246	○	-	-
83	loss	chrX:148,700,413-148,715,061	14,649	○	-	-
84	loss	chrY:13,637,403-13,868,434	231,032	-	○	-
85	loss	chrY:22,224,764-22,486,672	261,909	-	○	-

○ : CNV is detected
— : CNV is not detected
△ : CNV is a possible mosaic gain

Supplemental Table 4. List of SNV/indels detected at exonic region in WGS. Related to Figure 4.

NC2		#4-3		#7-1									Origin		NC2		#4-3		#7-1	
Genomon	Genomon2	Genomon	Genomon2	Genomon	Genomon2	Chr	Start	End	Ref	Alt	Func.refGene	Gene.refGene	coverage	alt_ratio	coverage	alt_ratio	coverage	alt_ratio	coverage	alt_ratio
○	○	○	○	-	-	chr8	145,254,085	145,254,085	C	T	exonic	MROH1	72	0.0%	44	18.2%	34	29.4%	47	0.0%
○	○	-	-	-	-	chr13	73,301,740	73,301,740	T	G	exonic	MZT1	40	0.0%	41	41.5%	43	0.0%	36	2.8%
○	○	-	-	-	-	chr20	3,673,216	3,673,216	C	T	exonic	SIGLEC1	60	0.0%	44	29.6%	47	0.0%	42	0.0%
○	-	-	-	-	-	chr2	125,192,138	125,192,138	G	T	exonic	CNTNAP5	51	0.0%	38	21.1%	54	0.0%	38	2.6%
-	○	○	○	-	-	chr14	31,771,711	31,771,711	G	T	exonic	HEATR5A	30	0.0%	33	15.2%	50	60.0%	35	0.0%
-	-	○	○	-	-	chr3	42,916,713	42,916,713	G	T	exonic	CYP8B1	54	0.0%	41	7.3%	46	54.4%	33	0.0%
-	-	○	○	-	-	chr3	65,428,513	65,428,513	C	A	exonic	MAGI1	41	0.0%	45	0.0%	43	58.1%	24	0.0%
-	-	○	○	-	-	chr3	194,118,222	194,118,222	G	A	exonic	GP5	46	0.0%	46	13.0%	52	46.2%	36	0.0%
-	-	○	○	-	-	chr4	16,035,028	16,035,038	ACAGCAACGAC	-	exonic	PROM1	52	0.0%	32	0.0%	53	45.3%	35	0.0%
-	-	○	○	-	-	chr5	179,545,801	179,545,801	A	T	exonic	RASGEF1C	60	0.0%	49	0.0%	64	56.3%	45	0.0%
-	-	○	○	-	-	chr6	46,679,244	46,679,244	G	A	exonic	PLA2G7	52	0.0%	42	0.0%	58	48.3%	34	0.0%
-	-	○	○	-	-	chr6	99,956,659	99,956,659	C	T	splicing	USP45	64	0.0%	58	10.3%	49	34.7%	38	0.0%
-	-	○	○	-	-	chr6	150,059,867	150,059,867	G	A	exonic	NUP43	41	0.0%	40	10.0%	49	57.1%	51	0.0%
-	-	○	○	-	-	chr6	151,646,998	151,646,998	G	A	exonic	AKAP12	38	0.0%	37	10.8%	50	44.0%	38	0.0%
-	-	○	○	-	-	chr8	2,048,819	2,048,819	C	T	exonic	MYOM2	48	0.0%	46	0.0%	39	41.0%	41	0.0%
-	-	○	○	-	-	chr11	49,078,765	49,078,765	C	A	exonic	TRIM64C	37	0.0%	46	10.9%	40	32.5%	36	0.0%
-	-	○	○	-	-	chr11	114,393,013	114,393,013	G	T	exonic	NXPE1	42	0.0%	42	7.1%	44	59.1%	27	0.0%
-	-	○	○	-	-	chr17	61,417,487	61,417,487	C	A	exonic	TANC2	48	0.0%	23	0.0%	50	44.0%	36	0.0%
-	-	○	○	-	-	chr19	15,905,528	15,905,528	G	A	exonic	OR10H5	44	0.0%	50	8.0%	41	46.3%	35	0.0%
-	-	○	○	-	-	chr19	46,257,754	46,257,754	C	T	exonic	BHMG1	53	0.0%	50	0.0%	45	51.1%	41	0.0%
-	-	○	○	-	-	chr19	48,547,154	48,547,154	C	T	exonic	CABP5	43	0.0%	38	5.3%	47	61.7%	39	0.0%
-	-	-	-	○	○	chr1	10,336,388	10,336,388	C	T	exonic	KIF1B	49	0.0%	36	0.0%	54	0.0%	35	42.9%
-	-	-	-	○	○	chr5	64,887,329	64,887,329	A	G	exonic	TRIM23	32	0.0%	39	0.0%	39	0.0%	36	44.4%
-	-	-	-	○	○	chr8	43,147,743	43,147,743	G	A	exonic	POTEA	34	0.0%	28	0.0%	33	0.0%	25	36.0%
-	-	-	-	○	○	chr14	68,040,102	68,040,102	C	T	exonic	PLEKHH1	57	0.0%	36	0.0%	55	0.0%	49	40.8%
-	-	-	-	○	○	chr18	76,755,107	76,755,110	CCAA	-	exonic	SALL3	47	0.0%	54	0.0%	47	0.0%	45	57.8%
-	-	-	-	○	○	chrX	129,146,629	129,146,629	C	A	exonic	BCORL1	31	0.0%	27	0.0%	23	0.0%	22	100.0%

○ : SNV/indel is detected
ー : SNV/indel is not detected
Genomon : a sequence analyzing tool to detect genomic variants
Genomon2 : an updated version of Genomon
Coverage: the depth of coverage at the target site
Alt ratio: a percentage of detected SNVs

Supplemental Experimental procedures

Vector construction

The polycistronic CRISPR-Cas3 vectors pPV-Dual_promoter-EF1α-2xNLS-Cascade+Cas3-iP (RD) (Addgene ID: 204619) were constructed in the previous study (Morisaka *et al*, 2019). To construct Cas3 all-in-one vector with mCherry (pPV-Dual_promoter-EF1α-2xNLS-Cascade+Cas3-iCA (RD)), the 2xNLS-Cas7-Cas5-Cas8-IRES-mCherry region was PCR amplified from the pPV-EF1α-2xNLS-Cas7-Cas5-Cas8-iCA vector (Addgene ID: 134922) by KOD ONE (TOYOBO) and purified with Wizard SV Gel and PCR Clean-Up System (Promega). The PCR product was inserted into the HindIII site of the pPV-EF1α-2xNLS-Cas11-Cas6-Cas3-A vector (Addgene ID: 134923) by In-Fusion reaction (Clontech).

To construct Cas3-crRNA and Cas9-sgRNA expression vectors, the synthesized crRNA/sgRNA sequences (Hokkaido System Science) were inserted into the two BbsI sites immediately downstream of the U6 promoter (pBSIIKS-U6v2-BbsI-C1, pBSIIKS-U6-BbsI-Cas9 or pPV-U6-crRNA-cloning-EF1α-BA (Addgene ID: 204623)). As for the crRNA/sgRNA sequences, Annealed oligonucleotides (Sense: "ACCG" 4 nt overhang + 32 nt target sequence (top strand), Antisense: "ACAC" 4 nt overhang + 32 nt target sequence (bottom strand) for crRNA, Sense: "ACCG" 4 nt overhang + 32 nt target sequence (top strand), Antisense: AAAC+ 32 nt target sequence (bottom strand) for sgRNA) were inserted between BbsI sites into the backbone vectors for ligation reaction. To design Cas9 sgRNA target sites, CRISPick (https://portals.broadinstitute.org/gppx/crispick/public) was used. To design Cas3 crRNA target sites, AAG (PAM) + 32 nt sequences (Cas3 induces deletions toward PAM direction) were extracted, and GGGenome (https://gggenome.dbcls.jp) was used to check their specificity to the human genome. Examples of designed crRNAs are shown in Figure S1A. Cas3-crRNA targeting DMD exon 45 and 55 are deposited into Addgene (pPV-C1-crRNA (DMD#20_DMD#23)-EF1a-BA (Addgene ID: 204620)). All the target sequences are shown in Supplemental Table 1.

To construct single-strand annealing (SSA) vectors, the genomic sequences around the *dystrophin* exon 45 or exon 55 region containing crRNA or sgRNA target site were amplified by PCR. The amplified fragments were inserted into the AfeI site of the pPV-EF1α-eGxxFP-iBA or pPV-EF1α-mRxxFP-iPA (Addgene ID: 204625) vector by In-Fusion reaction. SSA vectors with 1.0 kb spacer were deposited into Addgene (pPV-EF1α-EGxxFP(DMDex45_10)-iP-A(Addgene ID: 204621) and pPV-EF1α-mRxxFP1(DMDex55_10)-iP-A (Addgene ID: 204622)).

To construct double-nick SSA vectors, single strand oligos containing two CRISPR target sites and a spacer (35 bp, 25 bp, or 15 bp) were annealed and amplified into double-strand DNA by PCR. The PCR fragments were inserted into the AfeI site of the pPV-EF1a-eGxxFP-iPA (Addgene ID: 204624) vector by an In-Fusion reaction.

To construct a double-nick EGFP SSA vector (EGxxFP) with 0 bp spacer, the pPV-EF1a-eGxxFP-iPA vector backbone region was amplified by PCR, and the target region oligos were cloned by In-Fusion reaction.

To construct a double-nick mRFP SSA vector (mRxxFP) with 0 bp spacer, pPV-EF1a-mRxxFP-iPA vector was digested with AfeI and the target region oligos were cloned by In-Fusion reaction.

Cell culture

HEK293T cells (CRL-3216, ATCC) were cultured in Dulbecco's modified Eagle's medium (DMEM) high glucose (Nacalai Tesque, Kyoto, Japan) supplemented with 10% FBS (BioSera North America, Kansas City, MO, USA). All iPSCs were cultured in

StemFit AK02N media (Ajinomoto, Tokyo, Japan) on iMatrix-511 silk (Matrixome, Osaka, Japan) coated cell culture dishes.

Establishment of human iPSCs

Healthy donor iPS cell line FF409B2 (Okita *et al*, 2011) and DMD patient-derived iPS cell line FF12020 lacking exon 46 and 47 (Uchimura *et al*, 2021) were established previously. To establish other DMD patient-derived iPSC lines, fibroblasts (exon 51-53 deletion) or the PBMCs (exon 48-52 deletion) were electroporated with the three episomal DNA vectors encoding OCT3/4, SOX2, KLF4, L-MYC, LIN28, and p53 carboxy-terminal dominant-negative fragment (mp53DD) cDNAs, as previously described (Hong *et al*, 2009; Okita *et al.*, 2011). Each patient-derived iPSC line was registered as CiRA00458 (exon 51-53 deletion) and CiRA00646 (exon 48-52 deletion), respectively.

PCR-based genotyping and TA cloning

To examine Cas3's deletion patterns, PCR was performed using QuickTaq (TOYOBO). In this PCR, the forward primer was designed at the dystrophin exon 45, and the reverse primer was designed at the exon 55. Specific bands appeared only when a large deletion was induced from exon 45 to exon 55. To check the size of PCR amplicons, we run agarose gel electrophoresis or TapeStation analysis with High Sensitivity D5000 ScreenTape (Agilent) according to the manufacturer's protocol.

For TA cloning, the ladder bands were cut from the gel and purified by Wizard® SV Gel and PCR Clean-Up System (Promega). Then, obtained DNA fragments were TA-cloned into pGEM-T Easy Vector (Promega). After transformation into DH5α, blue-white selection and miniprep by Wizard® SV Minipreps DNA Purification Systems (Promega), inserted sequences in those plasmids were Sanger sequenced. All PCR primers are shown in Supplemental Table 2.

Skeletal muscle differentiation by doxycycline-inducible MYOD1 expression

Skeletal muscle differentiation of iPSCs was performed as described previously (Shoji *et al*, 2015; Tanaka *et al*, 2013; Uchimura *et al*, 2017). Briefly, the doxycycline-inducible human *MYOD1 piggyBac* vector (containing neomycin or puromycin resistance cassette) was integrated into the genome by electroporation together with transposase expression vector pHL-EF1a-hcPBase-A. Then, stable cells were selected with neomycin (FF12020) or puromycin (CiRA00458, CiRA00646) for 7 days. Subsequently, 1×10^5 cells/well were seeded into a 6-well plate coated with Matrigel in AK02N StemFit media with 10 μM Y-27632 (TOCRIS, MN, USA). The next day, the media was changed to Primate ES Cell Media (ReproCELL Inc., Kanagawa, Japan). On day 3, the media was changed to Primate ES Cell Media containing 2 μM doxycycline to induce *MYOD1* expression. On day 4, media was changed to skeletal muscle differentiation media composed of alpha Minimal Essential Medium (α-MEM; Nacalai Tesque) with 5% KSR (Thermo Fisher SCIENTIFIC), 100 μM 2-Mercaptoethanol and 2 μM doxycycline. After this, medium change was performed every day with the skeletal muscle differentiation media until Day 7.

Detection of proteins by Wes western blotting system

Skeletal muscle cells differentiated from iPSCs were lysed with RIPA buffer (Thermo Fisher Scientific) containing cOmplete Protease Inhibitor Cocktail (Roche). The protein concentration was quantified by the Pierce BCA Protein Assay Kit (Thermo Fisher Scientific). One microgram of samples was loaded on a Wes Simple Western system (ProteinSimple) with a 66-440 kDa Separation Module (ProteinSimple SM-W006) and the Anti-Mouse Detection Module (ProteinSimple DM-002). For detecting Dystrophin, a mouse anti-dystrophin (Rod domain) monoclonal antibody (DYS1; 1:100, Leica) and anti-mouse IgG HRP-linked antibody (ProteinSimple, 042-205) were used. For MHC

detection, the primary antibody was anti-myosin heavy chain (MAB4470, Mouse monoclonal, 1:400, R&D systems, Inc.) and the second antibody was anti-mouse IgG HRP-linked antibody (ProteinSimple, 042-205).

Assessment of exon skipping by RT-PCR

Total RNA was extracted from skeletal muscle cells differentiated from iPSCs using a NucleoSpin RNA Kit (Mecherey-Nagel GmbH & Co. KG), and cDNA was synthesized using ReverTra Ace qPCR RT Master Mix (Toyobo). Then, the exon 45-55 region was amplified by PrimeSTAR GXL DNA Polymerase (TaKaRa Bio). Sequences of primer pairs were listed in Supplemental Table 2. Multi-exon skipping was assessed by Agilent 4200 Tape Station (Agilent Technologies). Obtained PCR products were also Sangar sequenced to check the Ex44-56 junction.

Immunocytochemical staining

Differentiated cells were fixed with PBS containing 2% paraformaldehyde for 10 min at room temperature. After washing with PBS for two rounds, the cells were blocked with Blocking One PBS solution (Nacalai Tesque) for 45 minutes. Following the blocking, the cells were incubated with the primary antibodies diluted with 10% Blocking One in PBS-T (0.2% Triton X100 in PBS) overnight at 4ºC. Then, the cells were washed with PBS-T for two rounds and incubated with the secondary antibodies for one hour at room temperature. For the primary antibodies, we used anti-dystrophin (ab15277, Rabbit polyclonal, 1:100, Abcam) and anti-myosin heavy chain (MAB4470, Mouse monoclonal, 1:400, R&D systems, Inc.). For the secondary antibodies, goat anti-rabbit IgG, Alexa Fluor 546 (A-11010, Invitrogen), and goat anti-mouse IgG, Alexa Fluor 488 (Invitrogen, A-11001), were used.

Whole genome sequencing and off-target analysis

To perform whole genome sequencing, DMD-iPSC line FF12020 with two different passage numbers (passage 32 and 50) and MES-induced subclones from each parental iPSC subclones (#7-1 and #4-3) were prepared. Genomic DNA was extracted from iPSCs by MonoFas cultured cell genome DNA extraction kit VI (ANIMOS). After preparing the DNA library using KAPA Hyper Prep Kit PCR-Free Kit and IDT for Illumina-TruSeq DNA UD Indexes, genomic sequence analysis was performed by NovaSeq 6000. The obtained FASTQ files from each sample were mapped to the human genome (hg19) by BWA-MEM (0.7.15), then duplicated reads were removed by novosort (1.03.0.9). Then, SNV/Indel call was performed using Genomon (1.0.1) and Genomon2 (2.3.0), CNV call was performed using Delly (0.7.3) and VarScan (2.4.2), with the original iPSC sample with the lowest passage numbers (P32) as a reference.

To search for potential Cas3-crRNA binding sites, we used GGGenome (https://gggenome.dbcls.jp/) with up to 8 bp mismatch allowance (25% of the total length of crRNA). Any mismatches at positions 6, 12, 18, 24, and 30 were accepted because these bases are not involved in target recognition. Then, the distances between the CNV (or SNV/indel) and the potential crRNA binding site were calculated for crRNA (DMD exon 45) and crRNA (DMD exon 55), respectively. The shortest distance was collected.

Supplementary References

Hong H, Takahashi K, Ichisaka T, Aoi T, Kanagawa O, Nakagawa M, Okita K, Yamanaka S (2009) Suppression of induced pluripotent stem cell generation by the p53-p21 pathway. *Nature* 460: 1132-1135

Morisaka H, Yoshimi K, Okuzaki Y, Gee P, Kunihiro Y, Sonpho E, Xu H, Sasakawa N, Naito Y, Nakada S *et al* (2019) CRISPR-Cas3 induces broad and unidirectional genome editing in human cells. *Nat Commun* 10: 5302

Okita K, Matsumura Y, Sato Y, Okada A, Morizane A, Okamoto S, Hong H, Nakagawa M, Tanabe K, Tezuka K *et al* (2011) A more efficient method to generate integration-free human iPS cells. *Nat Methods* 8: 409-412

Shoji E, Sakurai H, Nishino T, Nakahata T, Heike T, Awaya T, Fujii N, Manabe Y, Matsuo M, Sehara-Fujisawa A (2015) Early pathogenesis of Duchenne muscular dystrophy modelled in patient-derived human induced pluripotent stem cells. *Sci Rep* 5: 12831

Tanaka A, Woltjen K, Miyake K, Hotta A, Ikeya M, Yamamoto T, Nishino T, Shoji E, Sehara-Fujisawa A, Manabe Y *et al* (2013) Efficient and reproducible myogenic differentiation from human iPS cells: prospects for modeling Miyoshi Myopathy in vitro. *PLoS One* 8: e61540

Uchimura T, Asano T, Nakata T, Hotta A, Sakurai H (2021) A muscle fatigue-like contractile decline was recapitulated using skeletal myotubes from Duchenne muscular dystrophy patient-derived iPSCs. *Cell Rep Med* 2: 100298

Uchimura T, Otomo J, Sato M, Sakurai H (2017) A human iPS cell myogenic differentiation system permitting high-throughput drug screening. *Stem Cell Res* 25: 98-106
